# Single-Cell Expression Profiling Reveals a Dynamic State of Cardiac Precursor Cells in the Early Mouse Embryo

**DOI:** 10.1371/journal.pone.0140831

**Published:** 2015-10-15

**Authors:** Ioannis Kokkinopoulos, Hidekazu Ishida, Rie Saba, Prashant Ruchaya, Claudia Cabrera, Monika Struebig, Michael Barnes, Anna Terry, Masahiro Kaneko, Yasunori Shintani, Steven Coppen, Hidetaka Shiratori, Torath Ameen, Charles Mein, Hiroshi Hamada, Ken Suzuki, Kenta Yashiro

**Affiliations:** 1 Translational Medicine and Therapeutics, William Harvey Research Institute, Barts and The London School of Medicine and Dentistry, Queen Mary University of London, London, United Kingdom; 2 Physiology and Pathology, University of São Paulo State – UNESP, Araraquara School of Dentistry, Araraquara, São Paulo, Brazil; 3 Clinical Pharmacology, William Harvey Research Institute, Barts and The London School of Medicine and Dentistry, Queen Mary University of London, London, United Kingdom; 4 Genome Centre, William Harvey Research Institute, Barts and The London School of Medicine and Dentistry, Queen Mary University of London, London, United Kingdom; 5 NIHR Barts Cardiovascular Biomedical Research Unit, Barts and The London School of Medicine and Dentistry, Queen Mary University of London, London, United Kingdom; 6 Department of Developmental Genetics, Graduate School of Frontier Biosciences, Osaka University, Suita, Osaka, Japan; University of Houston, UNITED STATES

## Abstract

In the early vertebrate embryo, cardiac progenitor/precursor cells (CPs) give rise to cardiac structures. Better understanding their biological character is critical to understand the heart development and to apply CPs for the clinical arena. However, our knowledge remains incomplete. With the use of single-cell expression profiling, we have now revealed rapid and dynamic changes in gene expression profiles of the embryonic CPs during the early phase after their segregation from the cardiac mesoderm. Progressively, the nascent mesodermal gene *Mesp1* terminated, and *Nkx2-5* ^+^/*Tbx5* ^+^ population rapidly replaced the *Tbx5* ^low+^ population as the expression of the cardiac genes *Tbx5* and *Nkx2-5* increased. At the Early Headfold stage, *Tbx5*-expressing CPs gradually showed a unique molecular signature with signs of cardiomyocyte differentiation. Lineage-tracing revealed a developmentally distinct characteristic of this population. They underwent progressive differentiation only towards the cardiomyocyte lineage corresponding to the first heart field rather than being maintained as a progenitor pool. More importantly, *Tbx5* likely plays an important role in a transcriptional network to regulate the distinct character of the FHF via a positive feedback loop to activate the robust expression of *Tbx5* in CPs. These data expands our knowledge on the behavior of CPs during the early phase of cardiac development, subsequently providing a platform for further study.

## Introduction

The heart is one of the first organs formed during vertebrate embryogenesis. Cardiac mesoderm cells emerge from the anterior portion of the primitive streak between the Early and Mid—Primitive Streak stages in the mouse embryo [1–4]. These cells migrate to the most anterior part of the lateral plate mesoderm (LPM), where cardiac progenitor/precursor cells (CPs) populate the heart field that will form the heart tube upon the Neural Plate stage [3, 5]. Subsequent morphogenetic events include the formation and looping of the heart tube, expansion of the ventricular and atrial chambers, and septation of the ventricles, atria, and outflow tract.

Lineage tracing experiments have led to the identification of the first heart field (FHF) and second heart field (SHF), from which the SHF CPs have been well characterised to date [1, 2, 6–8]. The SHF derives from cells of the subpharyngeal mesoderm [6, 9]. This population is localized initially in the mediodorsal region neighboring the FHF at E7.5 in the mouse embryo. Continuous addition of cells from CPs of the SHF to the arterial and venous poles of the heart tube as well as to the atrial septum occur until the separated systemic and pulmonary circulation is completed, underling their contribution to the right ventricle, outflow tract, and parts of the atria. The multipotency of SHF CPs gives rise to cardiomyocytes, electric conduction system, smooth muscle and endocardial/endothelial cells [10]. In contrast, the FHF gives rise to the first differentiated cardiomyocytes in the anterior splanchnopleuric layer of the LPM and directly contributes to the linear primitive heart tube [3, 11–15]. Although the detailed mechanisms regulating the segregation of the two heart fields remain unknown, it has been indicated that the FHF’s specification precedes that of the SHF in the primitive streak at Primitive Streak stage [4, 13, 14, 16]. The expression of the transcription factor *Tbx5* and potassium ion channel *Hcn4* at E7.5 were shown to be specific to the FHF, although the expression pattern of both genes are dynamically shifted in later stages of embryo development [11, 12, 17, 18]. *Tbx5* expression is also suggested to start at the Primitive Streak stage [14], whereas *Hcn4* likely starts after the Late Headfold stage [4, 11, 12]. Recent lineage tracing experiments indicate that the FHF contributes mainly to the left ventricle and portions of the atria [12–14]. In addition, different from the SHF, the FHF CPs marked by *Hcn4* and the FHF progenitor derived from the bHLH transcription factor *Mesp1* ^*+*^ cardiac mesoderm cells were shown to be unipotent [12, 13]. *Hcn4-*expressing FHF CPs contribute to the cardiomyocyte lineage, including the electric conduction system, whereas *Mesp1*-expressing FHF progenitors develop into cardiomyocytes or endocardium cells. Although the outline of the segregation and lineage tree of CPs including the FHF have been uncovered, the segregation from where the cardiac mesoderm terminates and what molecular mechanism underlies the segregation of CPs from the cardiac mesoderm remains largely unknown. Thus, further detailed elucidation of CPs at the early developmental stages will provide a better understanding of this aspect of embryonic CPs.

In order to intricately characterize the mouse embryonic CPs from the Neural Plate to the Headfold stage where CPs markers *Nkx2-5* and *Tbx5* are activated, we studied single-cell expression profiles from these stages. We demonstrate here; 1) a dynamic shift of CPs within a short period of time, underscoring the distinct expression profiles of the FHF and SHF at a single-cell resolution, 2) the unipotent character of *Tbx5* expressing CPs, which has not yet been clearly indicated, and 3) the existence of a positive feedback loop to fully activate the early *Tbx5* expression, suggested to be essential for cardiomyocyte differentiation unipotency of the FHF.

## Material and Methods

### Animals

The BAC transgene *Tbx5* ^CreERT2^ was constructed from the BAC clone RP23-267B15 [19] by replacement of exon 2 of *Tbx5* with a *CreERT2* cassette at the first methionine of the open reading frame in EL250 cells as previously described [20]. To perform recombination of BAC, PCR products for left-arm (5A SalI-EcoRV fragment) and right-arm (3A EcoRV-NotI fragment) fragments were amplified with the primer sets as follows; *Tbx5*-5A-F primer 5’ SalI site-caaaaataccgacgcctta-3’, *Tbx5*-5A-R primer 5’-EcoRV site-tgcgcaggggttcctg-3’, *Tbx5*-3A-F primer 5’-EcoRV site-cgatacagatgagggcttt-3’, and *Tbx5*-3A-R primer 5’-NotI site-ttatctggcccgttgttagc-3’. [5A SalI-EcoRV] fragment and [3A EcoRV-NotI] fragment were simultaneously cloned into pBluescript as [5A+3A SalI-EcoRV] fragment. After digestion via EcoRV, blunt-ended *CreERT2-FRT-neo* ^*R*^-*FRT* cassette was inserted between 5A left- and 3A right-arms. The EL250 cells transformed with RP23-267B15 BAC clone were subjected to electroporation with [5A-*CreERT2-FRT-neo* ^*R*^ *-FRT-*3A] fragment and selection using kanamycin brought BAC transgene with knock-in of [5A-*CreERT2-FRT-neo* ^*R*^ *-FRT-*3A] into *Tbx5* gene. Following the removal of the *neo* ^*R*^ cassette from this transgene via arabinose treatment (Flp induction), this genetically modified BAC clone (BAC transgene) was prepared and used for microinjection. BAC transgenic mice via microinjection were also generated as described previously [20]. The transgene recapitulated the expression pattern of endogenous *Tbx5* from the Neural Plate stage to the Headfold stage in embryos of five independent transgenic lines. Two of these lines (#3 and #28) that were the most efficient with regard to recombination at the *ROSA26* ^lacZ^ *Cre* reporter allele after tamoxifen administration in pregnant female mice at E7.5 [21, 22], were used for the present study. For staging the embryos, dissected embryos were classified according to their morphological features to identify the precise developmental stage instead of the embryonic day staging because of the frequent stage variation among litters [4]. Vaginal plug detection was set as E0.5. For lineage tracing *in vivo*, tamoxifen (0.1 mg per g of body weight) was administered by oral gavage to pregnant *ROSA26 Cre* reporter mice as previously described [21–23]. For teratoma formation assay, ES cells were injected subcutaneously together with Matrigel (BD Biosciences) into CD1 Nude/Nude mice (Charles River). The resulting tumours were dissected, embedded in paraffin, serially sectioned, and stained with hematoxylin-eosin (Sigma). All animals were kept as SPF grade. All animal procedures in this project were carried out under the project licenses (70/7254 and 70/7449) approved by the Home Office according to the Animals (Scientific Procedures) Act 1986 in the UK or under the approval from the Osaka University Animal Experimentation Committee (license Number: FBS-12-019) in Japan.

### Single-Cell cDNA Expression Profiling

Embryos of Early allantoic Bud (EB), Late allantoic Bud (LB), Early Head Fold (EHF), and the Early Somite stages were dissected, and the yolk sac and posterior portion of the embryo were removed as much as possible. The tissue was then dissociated into single cells by incubation with 0.05% trypsin/EDTA (Gibco) for 7 min at 37°C. The single cells were suspended in Hepes-buffered DMEM (phenol red free, Gibco) containing 0.4% polyvinylpyrrolidone (Sigma) and transferred to a non-coated petri dish. Each single cell was subsequently transferred to a reaction tube with a capillary pipette for cDNA preparation as previously described [24, 25]. PCR analysis of marker gene expression was performed with the primers listed in S1 Table to validate the cell of origin for each single-cell cDNA preparation. The Taqman assay was performed with an ABI7900HT system (Applied Biosystems). The primers and 6-fluorescein amidite (FAM)–conjugated probes are listed in S2 Table. Deep sequencing of single-cell cDNAs was performed with an Illumina GA IIx as previously described [25]. Aligned reads were annotated, normalized as RPM (reads per million), and subjected to statistical analysis, including one-way ANOVA and PCA, with Partek Genomic Suite 6.6 as previously described (S3 Table) [25]. Gene Ontology enrichment analysis was performed by Panther (http://www.pantherdb.org/) [26] and KEGG Annotation (http://www.genome.jp/kegg/annotation/) [27]. The sequence data have been submitted to NCBI Gene Expression Omnibus (GEO, http://www.ncbi.nlm.nih.gov/geo) under the accession number GSE63796.

### *In situ* hybridization

Whole mount *in situ* hybridization and *in situ* hybridization on sections were performed as previously described [20, 28, 29]. A probe for *Tbx5* was kindly provided by B. Bruneau, for *Nkx2-5* by R. Harvey, for *Isl1* by S. Evans, for *Mesp1* by Y. Saga, for *Myl7* by M. Shirai, and for *Myl2* by T. Mohun. Images were acquired with a Leica M205FA stereomicroscope and DFC310 FX digital camera.

### Pulse-Chase Lineage Tracing

Embryos of *Tbx5* ^CreERT2^/*ROSA26* ^eYFP/eYFP^ mice were dissected, and only those at the LB or EHF stage were studied. The embryos were incubated for 3 h in DMEM supplemented with 75% rat serum and 1 μM of 4-hydroxytamoxifen (Sigma) and then washed three times with Hepes-buffered DMEM (Gibco) to remove any residual drug. Whole-embryo culture was performed as previously described, with or without 1 μM 4-hydroxytamoxifen for 24 hours up to early somite stage for *ex vivo* lineage trace [28, 30]. For long culture, the dissected anterior portion of each embryo already exposed to 4-hydroxytamoxifen for 3 h was seeded on gelatin-coated Lab-Tek Chamber Slides (Nunc) after washing three times with Hepes-buffered DMEM to remove tamoxifen. The explants were cultured for 6 days without 4-hydroxytamoxifen and fixed for 10 min at 4°C with 4% paraformaldehyde in PBS prior to immunofluorescence staining.

### Derivation of mouse ES Cells

ES cell derivation was performed as previously described [31]. Blastocysts were harvested at E3.5 from pregnant *ROSA26* ^eYFP/eYFP^ mice that had been crossed with *Tbx5* ^CreERT2^/*ROSA26* ^eYFP/eYFP^ transgenic males. The hatched blastcycts were cultured on a feeder layer of mouse embryonic fibroblasts in iSTEM Embryonic Stem Cell Culture Medium (StemCells) supplemented with leukemia inhibitory factor (LIF) (ESGRO, Merck Millipore) at 1000 U/ml. After 5 to 6 days, the ES cell aggregates were isolated by exposure to trypsin and seeded again on a feeder layer. The resulting clones were isolated and expanded further. Among the established ES cell colonies, we selected three independent clones with the male karyotype (represented by the presence of *Sry*) for further study. The ES cells were maintained in ESGRO Complete PLUS Clonal Grade Medium (Millipore) without feeder cells but with the addition of LIF (1000 U/ml).

### Cardiomyogenic Differentiation of mouse ES cells

Cardiac differentiation of mouse ES cells was induced via embryoid body formation followed by the culture of formed embryoid bodies on the gelatin-coated culture dish in DMEM supplemented with 10% FBS, which allows stochastic cardiac differentiation in ES cells, in order to exclude the possibility that a defined media preferentially induce the FHF identity. Cardiac differentiation, especially for FACS analysis, was induced using a previously described protocol with a modification [32]. Differentiation was induced either by embryoid body formation or in monolayer culture. Undifferentiated colonies were passaged for cell counting and reseeded at a density of 5000 cells/mm^2^ on glass coverslips coated with gelatin, fibronectin, or laminin. Colonies were exposed either to DMEM supplemented with 10% FBS throughout the differentiation process or to defined media for three-step differentiation. For three-step differentiation, cells isolated by exposure to trypsin were incubated for 1 day in Iscove's modified Dulbecco's medium (IMDM)–Ham’s F12 (Invitrogen) supplemented with N2 and B27 supplements (Gibco), 10% bovine serum albumin (Sigma), 2mM L-glutamine (Gibco), penicillin-streptomycin (Gibco), 0.5 mM ascorbic acid (Sigma), and 150 mM monothioglycerol (Sigma). For mesodermal induction and patterning, cells were exposed for 2 days to different concentrations of Activin A (5 and 8 ng/ml, R&D Systems) and bone morphogenetic protein 4 (0.1, 0.25, and 0.5 ng/ml; R&D Systems) together with human vascular endothelial growth factor (VEGF, 5 ng/ml; R&D Systems). Cardiac specification was induced by exposure of the cells to StemPro-34 SF medium (Gibco) supplemented with 2 mM L-glutamine, 0.5 mM ascorbic acid, human VEGF (5 ng/ml), human basic fibroblast growth factor (10 ng/ml, R&D Systems), and human fibroblast growth factor 10 (50 ng/ml, R&D Systems). The medium was changed every other day, and cells were analysed after 10 to 14 days *in vitro*.

### Immunostaining

Immunofluorescence and immunoblot analysis of cultured cells and tissue sections was performed as previously described [28, 33, 34]. Alkaline phosphatase staining was performed with an Alkaline-Phosphatase Detection Kit (Millipore). Immunofluorescence images of tissue sections were acquired with Zeiss LSM510 confocal and Keyence BZ8000 fluorescence microscopes. Chemiluminescent Western blot data was acquired with Alpha Imager HP Imaging System (Alpha Innotech). Primary antibodies were as follows: TBX5 (1/100 dilution for histology and 1/1000 dilution for Western blot, rabbit polyclonal, Sigma, Catalogue number HPA008786), GFP for the detection of eYFP (1/100 dilution, mouse monoclonal, Invitrogen, Catalogue number A11120; 1/100 dilution, rabbit polyclonal, Molecular Probes, Catalogue number A6455; 1/4000 dilution, goat polyclonal, Abcam, Catalogue number AB38689), TNNT2 (1/100 dilution, goat polyclonal, HyTest, Catalogue number 4T19/2), ACTA2 (1/100 dilution, rabbit polyclonal, Abcam, Catalogue number AB32575), HCN4 (1/100 dilution, rabbit polyclonal, Millipore, Catalogue number AB5808), NKX2-5 (1/100 dilution, goat polyclonal, Santa Cruz, Catalogue number sc8697), PECAM1 (1/100 dilution, rat polyclonal, Pharmingen, Catalogue number 550274), Estrogen Receptor α (not diluted, ESR; rabbit monoclonal, Abcam, Catalogue number AB27595), SSEA1 (1/1000 dilution, mouse monoclonal, Abcam, Catalogue number AB16285) and αTubulin (1/200 dilution, mouse monoclonal, Sigma, Catalogue number T5168).

### Flow Cytometry

Mouse ES cells on day 14 of cardiac differentiation were analyzed with the use of an LSR Fortessa II Analyzer (BD Biosciences) and FACSDiva 7.0 software as previously described [33]. In brief, the cultured cells were isolated by exposure to 0.25% trypsin/EDTA (Sigma-Aldrich) for 6 min at 37°C under 5% CO_2_. These were then fixed and permeabilized with IntraStain Reagent A and B of an IntraStain kit (DAKO) according to the manufacturer’s protocol. Primary antibodies included antibodies to TNNT2 (1/200 dilution, goat polyclonal, HyTest) and to GFP (1/500 dilution, rabbit polyclonal, Molecular Probes). Secondary antibodies included Alexa Fluor 647–conjugated donkey antibodies to goat immunoglobulin G and Alexa Fluor 488–conjugated donkey antibodies to rabbit immunoglobulin G (Molecular Probes). To assay apoptosis, Annexin V positive apoptotic cells were measured using a Dead Cell Apoptosis Kit with Annexin V Alexa Fluor^™^ 488 & Propidium Iodide Kit (Molecular Probes) according to the manufacturer’s protocol.

### Gene Targeting with CRISPR/Cas9

The oligonucleotide for a sgRNA was cloned into the pX330 vector (Addgene), and electroporated with the pIRES-puro expression vector (Clontech) into ES cells, followed by puromycin (Sigma) selection as previously described [35]. The oligonucleotides used for sgRNA are listed in S4 Table. The genomic deletion was confirmed by sequencing of Polymerase chain reaction (PCR) products obtained from the targeted genomic sequence.

## Results

### The single-cell expression profile of the earliest cardiac cells is highly dynamic

**Fig 1:**
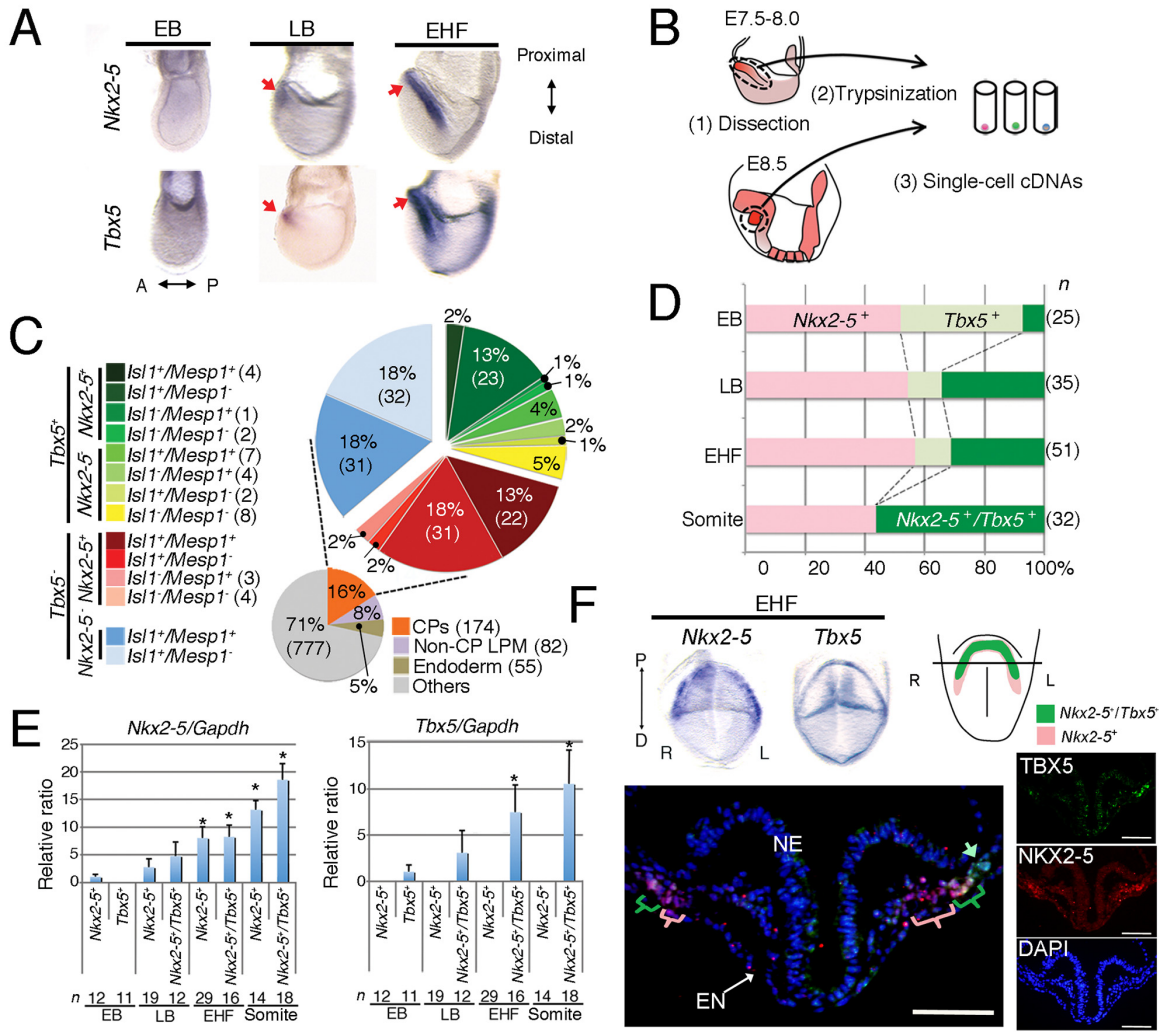
Single-cell expression profiling of the earliest CPs. (A) WISH analysis of *Nkx2-5* and *Tbx5* expression in mouse embryos at EB, late bud (LB), and EHF stages. Embryos are shown in left lateral view. A; anterior, P; posterior, Red arrows; the most anterior part of the embryo. (B) Strategy for generation of single-cell cDNA preparations. (C) Classification of single-cell cDNA preparations of CPs from EB, LB, and EHF stages by PCR analysis of marker genes. The number of preparations is shown in parentheses. (D) Subpopulation shift between EB and Somite stages. (E) Taqman assay for *Nkx2-5* and *Tbx5* on constructed single-cell cDNA preparations. (F) Distribution of *Nkx2-5*-expressing CPs and *Tbx5*-expressing CPs in EHF stage embryo. The pictures of the embryos in the upper panel indicate whole mount *in situ* hybridization for *Nkx2-5* and *Tbx5* in EHF stage embryos in the frontal view. Note the area of *Nkx2-5* is wider than that of *Tbx5*. Fluorescence images indicate the immunostained EHF stage mouse embryo for NKX2-5 and TBX5. The illustration at the upper right panel shows section plane of fluorescence image. D; distal, EN; endoderm, NE; neural ectoderm. Blue; 4ʹ,6-diamidino-2-phenylindole (DAPI), Green; TBX5, Red; NKX2-5, L; left, P; proximal, R; right. Scale bar; 100 μm.

To characterize the CPs, we analysed single-cell cDNA profiles for the micro-dissected heart-forming region of mouse embryos from the Early Allantoic bud (EB) stage to the Early Headfold (EHF) stage, where *Nkx2-5* and *Tbx5* expression are initiated (Fig 1A and 1B). We excluded non—cardiac cell cDNA preparations on the basis of the markers *Sox17* for endoderm, and *Sox2* for neural ectoderm (Fig 1C) [36–38]. *Cfc1* was used for a marker of LPM and it should be positively expressed in CPs. We identified *Sox17* ^−^/*Sox2* ^−^/*Cfc1* ^+^/*Nkx2-5* ^+^ and/or *Tbx5* ^+^ cells as candidates for FHF CPs. PCR amplification of cDNAs revealed expression of *Nkx2-5* and *Tbx5* from the EB stage, whereas we did not detect their expression by whole-mount *in situ* hybridization (WISH) at this stage (Fig 1A and 1C–1E). This finding appears consistent with the segregation of the FHF and SHF within the primitive streak [13, 14]. Among a total of 1088 single-cell cDNA preparations obtained from the EB to Somite stages, we identified 111 preparations (those other than the ones of medium and light blue colours in the CP pie chart shown in Fig 1C) as candidates for FHF CPs. We also detected *Isl1* ^+^ LPM cells negative for *Nkx2-5* and *Tbx5* expression, which must correspond to the most primitive SHF cells (the medium and light blue colours in Fig 1C). Of note, most *Tbx5*-negative CPs were *Isl1* positive (67% versus 4% of all CPs in Fig 1C). We then classified CP cDNA preparations chronologically in terms of *Nkx2-5* and *Tbx5* expression (Fig 1D). Consistent with the results of WISH analysis, the abundance of *Nkx2-5* and *Tbx5* mRNAs increased gradually (Fig 1E and 1F), with the number of double-positive CPs for *Nkx2-5* and *Tbx5* (*Nkx2-5* ^+^/*Tbx5* ^+^) increasing up to the somite stage at the expense of *Tbx5* single-positive (*Tbx5* ^+^) CPs (Fig 1D). Given the specificity of the earliest *Tbx5* for the FHF and the induction of *Tbx5* expression at the Primitive Streak stage [14], this subpopulation shift suggests that *Tbx5*-expressing FHF CPs appear initially as *Tbx5* ^*+*^ which later become *Nkx2-5* ^*+*^ */Tbx5* ^*+*^. Although the earliest expression of both *Nkx2-5* and *Tbx5* has been regarded as a marker for the cardiac crescent (FHF), the region of *Nkx2-5* expression was not identical to that of *Tbx5* expression (Fig 1D and 1F and S1 Fig) [1, 39]. The area of *Nkx2-*5 expression expanded more widely toward the medial region than that of *Tbx5*, suggesting that only *Tbx5-*expressing cells at the EHF stage constitutes the FHF. Alternatively, the data suggests that the cardiac crescent could harbour a heterogeneous mixture containing *Nkx2-5* ^low+^ cells that later activate *Tbx5*.

**Fig 2:**
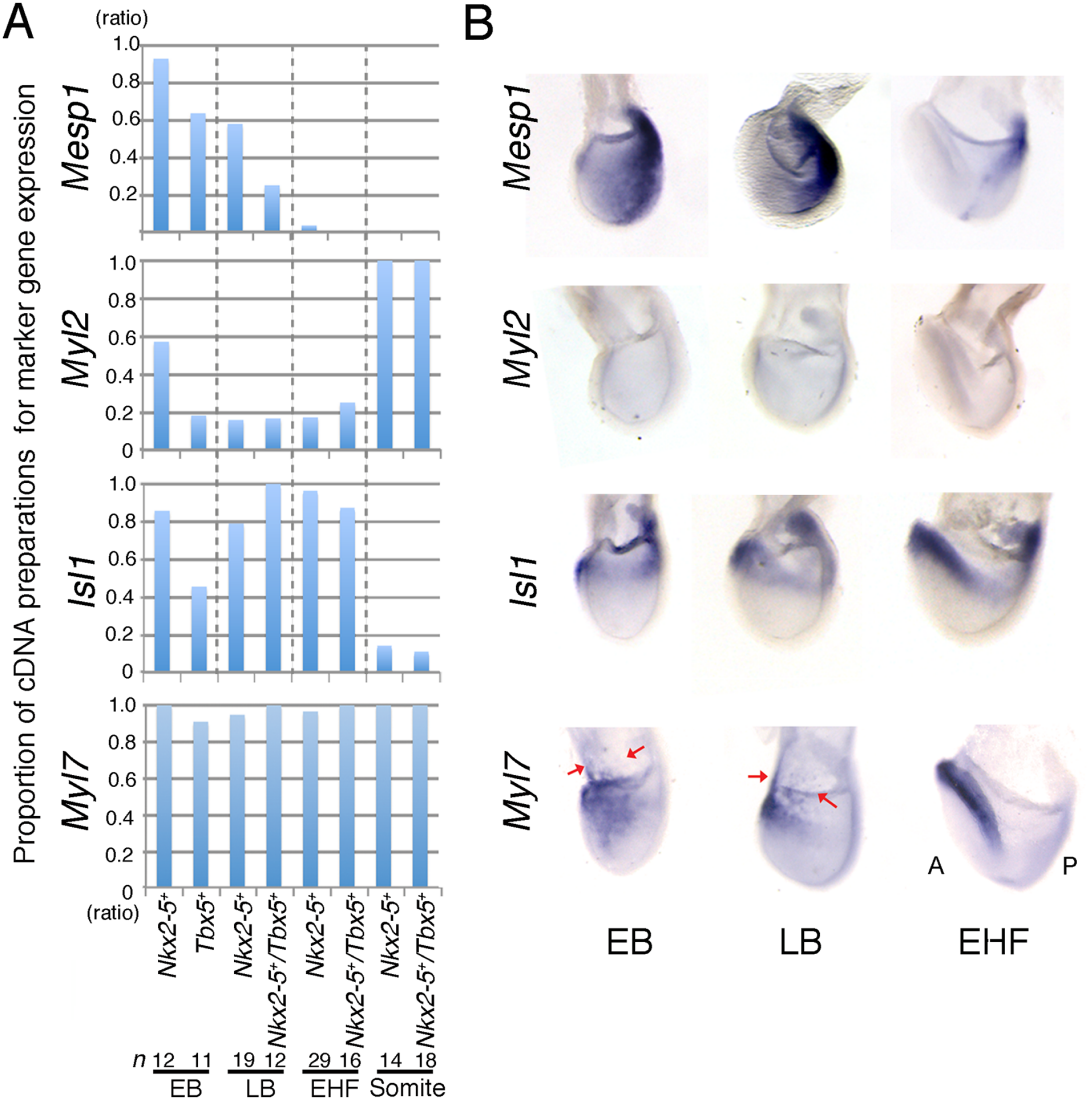
Dynamic Changes in the Expression Profiles of CPs from the EB to EHF Stages. (A) Proportion of single-cell cDNA preparations positive for *Mesp1*, *Myl2*, *Isl1*, and *Myl7* expression at the indicated embryonic stages as determined by PCR analysis. (B) WISH analysis of *Mesp1*, *Myl2*, *Isl1*, and *Myl7* expression in the mouse embryo at the EB, LB, and EHF stages. Embryos are shown in the left lateral view. Expression of *Mesp1* was detected at a low level in the anterior mesoderm at the EB stage. *Myl2* was not detected as expected by PCR analysis on single cell cDNA preparations in (A). A; anterior, P; posterior.

To characterize each of these subpopulations further, we scored cDNA preparations chronologically for the ratio of cells positive for the expression of additional cardiac marker genes by PCR (Fig 2A and 2B). Most CPs at the EB stage still expressed the cardiac progenitor marker *Mesp1* [1, 40]. The expression of *Mesp1* was almost completely down-regulated within the heart fields by the EHF stage. A terminal differentiation marker of cardiomyocytes, *Myl2*, was not expressed in a substantial proportion of cells until the somite stage, consistent with WISH data (Fig 2B) [41]. Thus, the clearly recognizable robust terminal differentiation likely takes place between the Late Headfold (LHF) stage (E8.0) and the somite stage. Unexpectedly, another cardiomyocyte marker *Myl7* was apparent in almost all CPs at all stages analysed and even among yolk sac (Fig 2B)[7, 42]. *Myl7* was expressed even at the EB stage, with its expression also previously having been detected within the primitive streak [13, 43], suggesting that its expression does not necessarily reflect a cardiomyocyte identity, at least up to the EHF stage. Also unexpectedly, *Isl1* that has been regarded as specific for the SHF was detected in most cDNA preparations in the EHF stage regardless of *Tbx5* expression, although fewer *Tbx5* ^+^ CPs in the EB stage expressed *Isl1* compared to *Nkx2-5* ^+^ CPs (Fig 2A). This evidence supports previous reports that *Isl1* is detected among the FHF population [44–47]. As indicated by previous studies, *Isl1* expression was down-regulated in differentiated cardiomyocytes at E8.5 (Fig 2A and 2B) [10, 44, 45]. Together, these results suggest that the expression profiles of CPs change rapidly from the EB to EHF stages; they might reflect a superimposition of different waves of progenitor cells and progressive differentiation.

**Fig 3:**
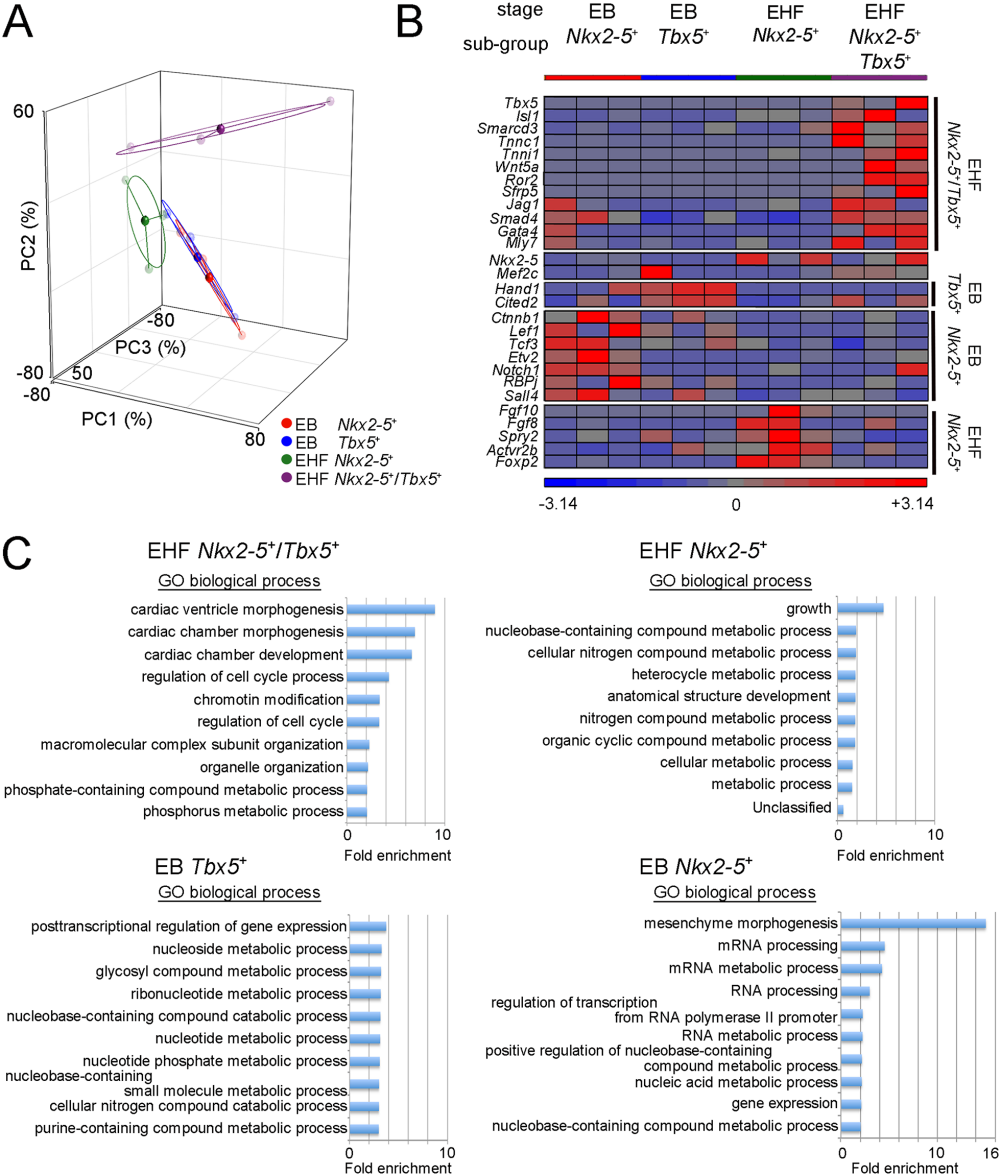
Distinct molecular signature of *Nkx2-5* ^+^/*Tbx5* ^+^ CPs at the EHF Stage. (A) PCA for the results of deep sequencing of single-cell cDNA preparations from cells of the indicated subpopulations. Circles of lighter colour represent each of the cell subpopulations, and those of darker colour represent the centroid for each cell subpopulation. Each ellipse indicates the standard deviation. (B) Heat-map of the expression of enriched key genes in each subpopulation. *Nkx2-5* and *Mef2c* were not enriched in any subpopulation. The intensity was calculated by the formula; z = (x-μ)/σ. z; intensity, x; value of Reads per Million, μ average, σ standard deviation. (C) Top ten categories of GO enrichment analysis in each subpopulation.

To explore further, we selected three typical single-cell cDNA preparations of EB and EHF stages and examined the expression profile by deep sequencing (Fig 3, S2 Fig and S3 Table). Principal component analysis (PCA) indicated that the expression profiles of *Nkx2-5* ^+^/*Tbx5* ^+^ FHF CPs at the EHF stage were relatively distinct from others (Fig 3A). The heterogeneity was observed to some extent even among cDNAs belonging to the same population, represented by the distance in PCA. We then filtered the genes showing a significant difference in expression in each cell subpopulation compared with the other cell subpopulations by one-way analysis of variance (ANOVA, with a non-adjusted *P* value of <0.05) (Fig 3B, S2 Fig and S5–S8 Tables). The enriched genes in *Nkx2-5* ^+^/*Tbx5* ^+^ FHF CPs included already known markers for CPs (*Tbx5*, *Smarcd3*, and *Gata4*), molecules of negative regulation of canonical WNT receptor signalling pathway (*Wnt5a*, *Ror2*, and *Sfrp5*) [48, 49], and sarcomere molecules (*Tnnc1*, *Tnni1*, and *Myl7*). This strongly suggests that the *Nkx2-5* ^+^/*Tbx5* ^+^ FHF CPs have already started terminal differentiation into cardiomyocytes to some extent, although we could not detect *Myl2* using PCR on single cell cDNA preparations and whole mount *in situ* hybridization (Fig 2). Potential inhibition of the WNT canonical pathway in this population is consistent with previous reports indicating that the WNT canonical pathway inhibits cardiomyocyte differentiation after cells have already been committed to a cardiac lineage [50, 51]. In contrast, the enriched genes in EHF *Nkx2-5* ^*+*^ CPs included the genes expressed in the SHF (*Fgf10* and *Fgf8*), suggesting that this population is the SHF CPs [52]. The enrichment of *Cited2* and *Hand1*, which are related to heart development, were observed in EB *Tbx5* ^*+*^ CPs, whereas the components of WNT canonical (*Ctnnb1*, *Lef1* and *Tcf3*) and NOTCH signalling (*Notch1* and *RBPj*) were enriched in EB *Nkx2-5* ^+^ CPs [53, 54]. The significant expression of the components of the WNT canonical pathway and the NOTCH pathway in early SHF CPs (EB *Nkx2-5* ^*+*^) supports a procardiogenic role of canonical WNT in the early phase and the requirement of the NOTCH1 pathway for cardiac differentiation [50, 55].

We next subjected these genes to Gene Ontology enrichment analyses (Fig 3C and S9–S12 Tables) [26]. The top three categories in *Nkx2-5* ^+^/*Tbx5* ^+^ FHF CPs were relevant to the cardiac development process. Additionally, the enrichment of genes involved in chromatin remodelling, organelle organization and metabolic processes (including molecules in mitochondria for lipid metabolism and oxidative phosphorylation) were also observed (S12 Table). This suggests a dynamic shift of the FHF CP in order to establish the unique structure and metabolism of the cardiomyocyte. Conversely, the other CPs did not show such specificity, rather general developmental (growth and anatomical structure development) and metabolic processes. EB *Tbx5* ^+^ CPs and EB *Nkx2-5* ^+^ CPs showed no sign of commitment, although markers involved in mesenchymal morphogenesis were observed in EB *Nkx2-5* ^+^ CPs. These results suggest that the differentiation of *Nkx2-5* ^+^/*Tbx5* ^+^ FHF CPs in the EHF stage is more advanced than the others [12, 13]. Consequently, it seems that *Tbx5* ^*+*^ FHF cells rapidly differentiate towards a cardiomyocyte fate after they activate *Nkx2-5*, moving away from maintaining a progenitor cell status for a prolonged period, a characteristic observed in CPs of the SHF [6].

### The production of bacterial artificial chromosome *Tbx5* ^CreERT2^ transgene to trace the FHF

**Fig 4:**
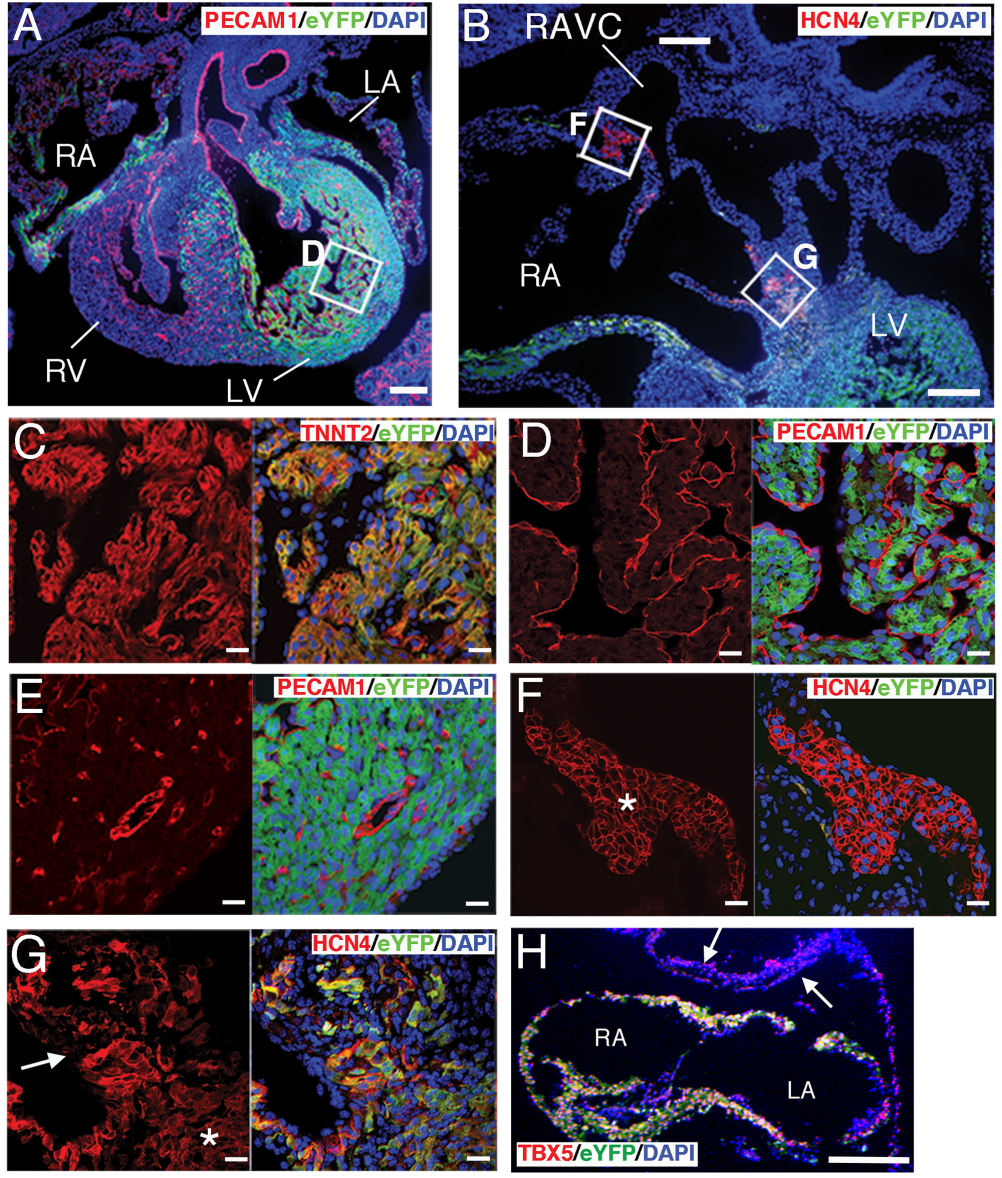
The FHF CPs marked by BAC *Tbx5* ^CreERT2^ transgene are unipotent cells that contribute only to the cardiomyocyte lineage. (A-G). Sections of the heart of E15.5 BAC *Tbx5* ^CreERT2^/*ROSA26* ^eYFP/eYFP^ mouse embryos treated with tamoxifen at E7.5 *in vivo* were subjected to confocal immunofluorescence analysis with antibodies to the indicated proteins. Nuclei were stained with DAPI. The boxed region in (A) is shown at higher magnification in (D), and those in (B) are shown at higher magnification in (F) and (G). Data are representative of three embryos. Asterisks indicate the sinoatrial node (F) or the bundle of His (G). The white arrow in (G) indicates the AV node. (H) Horizontal section stained with indicated antibodies at the atrial level of a BAC *Tbx5* ^CreERT2^/*ROSA26* ^eYFP/eYFP^ mouse embryo at E10.5 after tamoxifen treatment at E7.5 *in vivo*. TBX5^+^ cells in the DMP (white arrows) were negative for eYFP. LA; left atrium, RA; right atrium, RAVC; right anterior vena cava. Scale bars: 100 μm (A and B), 20 μm (C–G) and 200 μm (H).

We next attempted to trace the distinct sub-population of *Tbx5*-expressing cells inside the heart. Macro-anatomical distribution of *Tbx5*-expressing cells have been appreciated thus far, however identifying the cell type progeny is yet to be elucidated in detail [14]. To prevent heart anomalies caused by modifying the endogenous *Tbx5* allele leading to haploinsufficiency, we generated a bacterial artificial chromosome (BAC) *Tbx5* ^CreERT2^ transgene, which allowed the tamoxifen-induced labelling of *Tbx5*-expressing CPs progeny from E7.5 in *ROSA26 Cre* reporter mice harbouring a *lacZ* or enhanced yellow fluorescent protein (eYFP) reporter gene (Fig 4A and S3A Fig) [22, 23, 56, 57]. The expression of the *CreERT2* transgene mimicked endogenous *Tbx5* expression pattern from the Neural Plate to the Headfold stage (S3B and S3C Fig). Tamoxifen treatment (0.1 mg per g of body weight) by oral gavage at E7.5 labelled the *Tbx5-*expressing cell progeny in the heart only, and not the forelimb bud, where *Tbx5* expression begins at E8.5 (somite 8) (S3D and S3E Fig) [17]. The amount of expressed CreERT2 protein was sufficient to detect via immunofluorescence from EB stage embryos (S4 Fig). As expected, further analysis revealed that the progeny of E7.5 *Tbx5*-expressing cells were found specifically among cardiomyocytes of the left ventricle, parts of both atria, and parts of the cardiac conduction system (atrioventricular node, and bundle of His) at E15.5, corresponding to the expected FHF distribution (Fig 4B–4G) [11–14, 16]. The unipotency of the *Tbx5*-expressing FHF was also consistent with recent observations [12, 13, 58–61]. Thus, this transgene enabled to trace *Tbx5*-expressing CPs of the earliest stage [19]. The dorsal mesenchymal protrusion (DMP) may also derive from cells that express *Tbx5* at E7.5. This population of the SHF is known to express *Tbx5* at E10.5 contributing to the atrial septum [18]. Interestingly, tracing of E7.5 *Tbx5*-expressing cells in the *ROSA26* ^eYFP^ reporter mouse embryos at E10.5 revealed that TBX5^+^ DMP cells did not include any eYFP^+^ cells (Fig 4H), suggesting that *Tbx5*-expressing CPs at E7.5 do not contribute to this population.

### Distinct behaviour of the FHF CPs defined by a transcriptional network involving *Tbx5*

**Fig 5:**
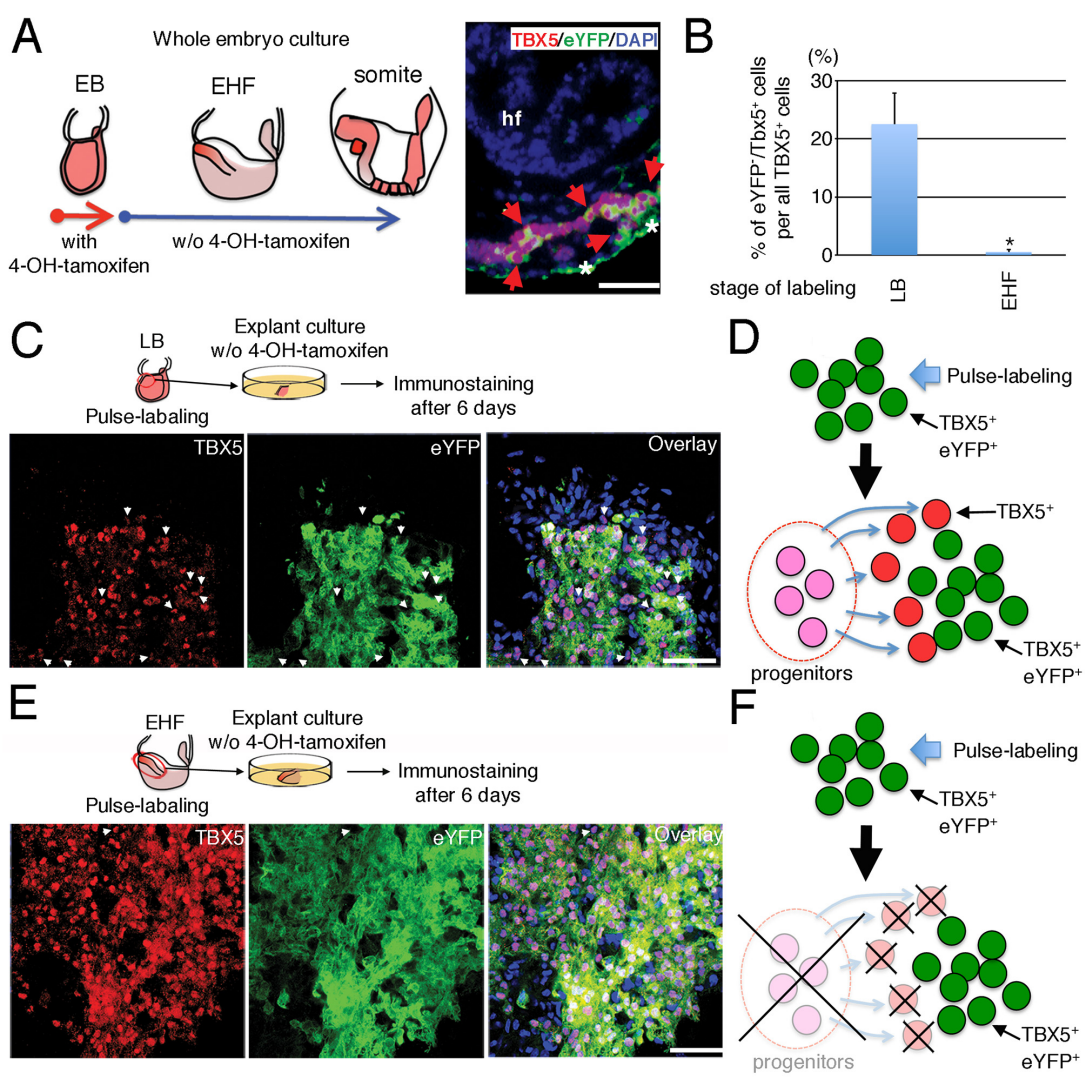
The production of *Tbx5*-expressing CPs terminates by the EHF stage. (A) Horizontal section of a BAC *Tbx5* ^CreERT2^/*ROSA26* ^eYFP/eYFP^ mouse embryo at the somite stage stained for TBX5 (red) and eYFP (green) is indicated in the right panel. This embryo was dissected at EB stage, pulse-labelled via transient exposure to 4-hydroxytamoxifen *in vitro* for 3 hours followed by the withdrawal of tamoxifen, and cultured for 24 hours via whole embryonic culture without 4-hydroxytamoxifen up to the early Somite stage. Red arrows indicate *Tbx5*-expressing cells labelled at the EB stage, and asterisks indicate the background signal in the endoderm due to the anti-mouse secondary antibodies on mouse section. Data are representative of five embryos. Left panel indicates the illustration of experimental design. hf, headfold. Scale bar, 50 μm. (B) Quantitative cytometric plot of the pulse-labelling experiment presented in C and E is indicated as mean ± SEM values. *P<0.05. (C) BAC *Tbx5* ^CreERT2^/*ROSA26* ^eYFP/eYFP^ embryos were pulse-labelled by transient exposure to 4-hydroxytamoxifen at the LB stage, and a tissue fragment from the anterior portion of each embryo was then cultured in the absence of 4-hydroxytamoxifen for 6 days before confocal immunofluorescence analysis with antibodies to TBX5 and to eYFP. Data are representative of four embryos. Blue fluorescence; DAPI, White arrowheads; TBX5^+^/eYFP^−^cells. Scale bar, 100 μm. (D) Schematic representation of CP eYFP^-^/TBX5^+^ progeny. (E) Embryos were analyzed as in (C) with the exception that pulse-labelling was performed at the EHF stage. Data are representative of six embryos. TBX5^+^/eYFP^−^cells were largely absent. Scale bar, 100 μm. (F) Schematic explanation of CP eYFP^+^/TBX5^+^-only progeny. A CP source producing TBX5^+^ cells is not present after pulse-labelling.

The evidence that *Tbx5* ^*+*^ CPs were replaced by *Nkx2-5* ^*+*^ */Tbx5* ^*+*^ CPs at later stage suggests that *Tbx5* ^*+*^ CPs become *Nkx2-5* ^*+*^ */Tbx5* ^*+*^ CPs (Fig 1D). To validate this hypothesis, we examined BAC *Tbx5* ^CreERT2^ /*ROSA26* ^eYFP/eYFP^ embryos subjected to pulse-chase labelling in culture transiently exposed to 4-hydroxytamoxifen (Fig 5 and S5 Fig). This system allowed the termination of genetic labelling of *Tbx5*-expressing cells almost immediately (within a few hours) after 4-hydroxytamoxifen removal from the culture medium (S5 Fig). If labelled at the EB stage (Fig 5A), we indeed detected eYFP^+^/TBX5^+^ cells in the heart tube at the somite stage (E8.25) (Fig 5A). Given that all *Tbx5-*expressing cells were *Nkx2-5* ^+^/*Tbx5* ^+^ at the somite stage (Fig 1D), the presence of eYFP^+^/TBX5^+^ cells in the heart tube of E8.25 embryos strongly suggests that *Tbx5* ^+^ cells at the EB stage became *Nkx2-5* ^+^/*Tbx5* ^+^ cells. However, we cannot exclude the possibility that *Nkx2-5* ^low+^ CPs at the EB stage also contribute to the FHF (*Nkx2-5* ^*+*^ */Tbx5* ^*+*^ CPs), because not all of TBX5-positive cells were eYFP-positive in this experiment (Fig 5A).

On the other hand, it is suggested that the production of *Tbx5*-expressing FHF CPs terminate by E8.5 demonstrated by the *Tbx5*-expressing cells labelled at E7.5 completely occupied the anatomical structure corresponding to the FHF (Fig 4). The question thus arises as to the duration of *Tbx5*-expressing FHF CPs being supplied by the cardiac mesoderm. To elucidate this, we utilized the pulse-chase system. If the production of *Tbx5*-expressing cells continued after pulse-labelling, TBX5^+^/eYFP^−^single positive cells should be observed (Fig 5D). Conversely, if the production of *Tbx5*-expressing cells has terminated before pulse-labelling, only TBX5^+^/eYFP^+^ double-positive cells should be detected (Fig 5F). Many TBX5^+^/eYFP^−^cells were detected when pulse-labelling was performed at the Late Allantoic Bud (LB) stage (Fig 5B and 5C), whereas almost all cells expressing TBX5 were eYFP^+^ when pulse-labelling was performed at the EHF stage (Fig 5B and 5E). These findings indicate that the production of *Tbx5*-expressing CPs continues at the LB stage but terminates before the EHF stage. This suggests that the progeny of FHF CPs increase in number as a result of proliferation of an already committed cell pool after the Headfold stage, rather than as a result of a continuous supply of cells from pre-established cardiac progenitors. Furthermore, the data suggest that *Nkx2-5* ^+^ cells at the EHF stage are from the SHF CPs, minimally contributing to the *Nkx2-5* ^+^/*Tbx5* ^+^ cell subpopulation after the EHF stage (Fig 1D). This idea is consistent with *Nkx2-5* ^+^ CPs expressing genes specifically from the SHF in deep sequencing analysis (Fig 3B). Thus, at the Headfold stage, *Tbx5*-expressing CPs are the FHF.

**Fig 6:**
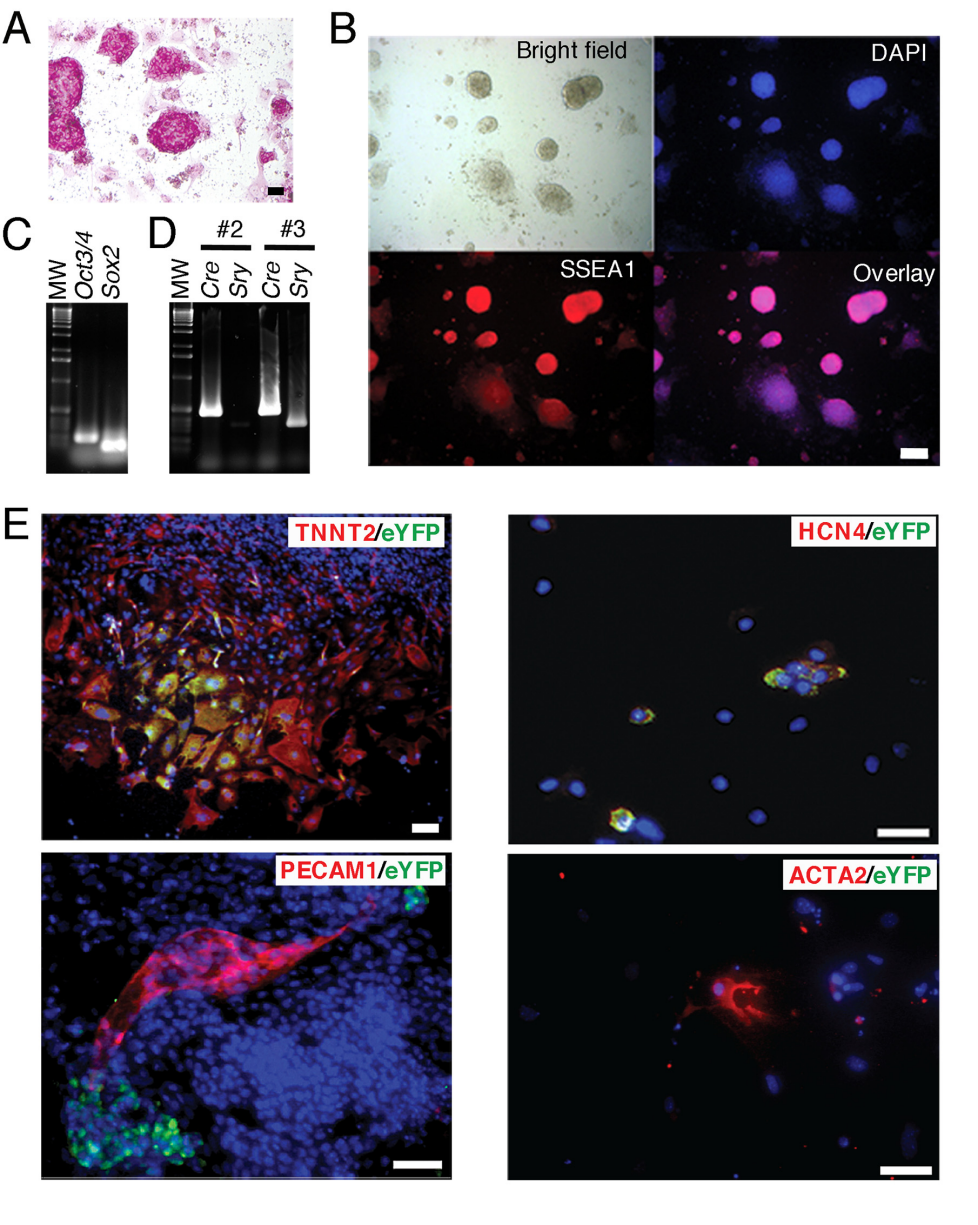
*Tbx5*-Expressing FHF cells derived from ES cells are unipotent. (A) Alkaline phosphatase staining for BAC *Tbx5* ^CreERT2^/*ROSA26* ^eYFP/eYFP^ ES cells. Scale bar, 100 μm. (B) BAC *Tbx5* ^CreERT2^/*ROSA26* ^eYFP/eYFP^ ES cells stained with SSEA1 antibody. Blue; DAPI. Scale bar, 100 μm. (C) RT-PCR analysis of *Oct3/4* and *Sox2* expression in BAC *Tbx5* ^CreERT2^/*ROSA26* ^eYFP/eYFP^ ES cells. (D) Genomic PCR analysis for *CreERT2* and *Sry* in isolated BAC *Tbx5* ^CreERT2^/*ROSA26* ^eYFP/eYFP^ ES cell lines. Only those with a male karyotype (*Sry* positive) were chosen for further experiments. Clones #2 and #3 are female and male, respectively. (E) BAC *Tbx5* ^CreERT2^ */ROSA26* ^eYFP/eYFP^ ES cells were induced to differentiate into cardiomyocytes *in vitro* in the presence of 4-hydroxytamoxifen. The cells at differentiation day 14 were then stained with antibodies to eYFP as well as with those to TNNT2, HCN4, PECAM1, or ACTA2A. Blue; DAPI. Scale bars, 100 μm.

To trace the FHF CPs *in vitro*, we derived embryonic stem (ES) cell lines from *Tbx5* ^CreERT2^/*ROSA26* ^eYFP/eYFP^ blastocysts (Fig 6A–6D and S6 Fig) and induced their cardiac differentiation in the presence of 4-hydroxytamoxifen (Fig 6E). The differentiated cells were examined on day 14 of differentiation. Using this *in vitro* ES cell differentiation method, beating foci of cardiomyocytes are usually recognized after day 10 of differentiation. Even if we did not use a defined medium that significantly drives ES cells to cardiomyocyte differentiation [32], we found that almost all eYFP^+^ cells differentiated into either TNNT2^+^ cardiomyocytes *in vitro*, suggesting that the cell fate of *Tbx5*-expressing cells is intrinsically determined. Although few in number, HCN4^+^ cells which are conduction system cells or primary myocardium of the FHF CPs, were observed [11, 12, 62]. However, eYFP^+^ endothelial cells (PECAM^+^) and smooth muscle cells (ACTA2^+^) were not found (Fig 6E).

**Fig 7:**
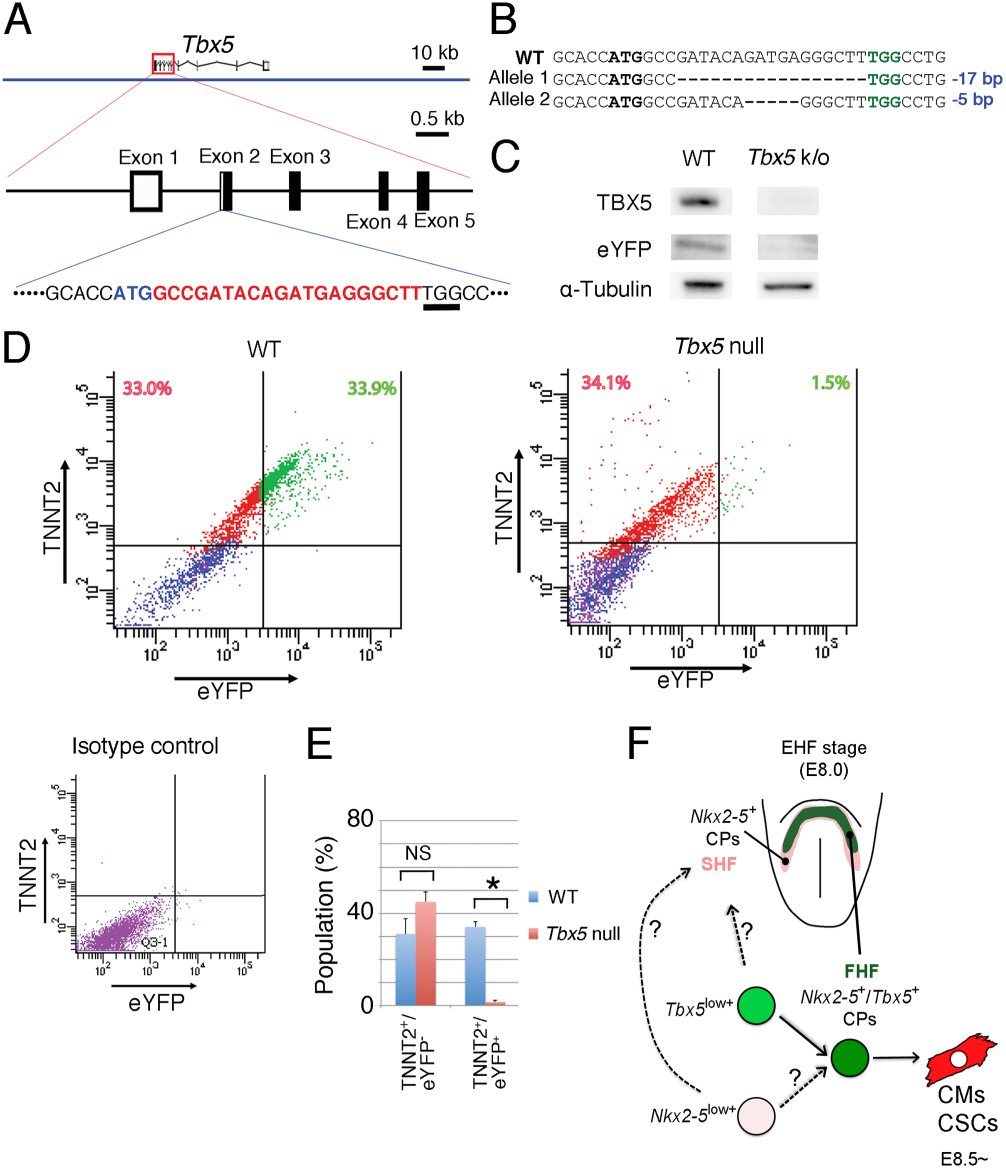
Existence of a positive feedback loop to activate the *Tbx5* gene. (A) An sgRNA was designed to target the sequence (shown in red) immediately downstream of the first methionine codon (ATG in blue). The underlined TGG sequence corresponds to the protospacer adjacent motif (PAM). (B) Obtained internal deletion alleles in a *Tbx5* null ES cell line. The first methionine codon (ATG) is shown in bold, and the PAM (TGG) is shown in green. (C) Immunoblot analysis of TBX5 and eYFP in differentiated BAC *Tbx5* ^CreERT2^/*ROSA26* ^eYFP/eYFP^ ES cells that were either wild type or null (k/o) for *Tbx5*. α-Tubulin was examined as a loading control. The whole blotted membrane is indicated as a whole in S8 Fig. (D) BAC *Tbx5* ^CreERT2^ */ROSA26* ^eYFP/eYFP^ ES cells either rendered *Tbx5* null by the CRISPR/Cas9 or left unmodified (WT) were induced to differentiate into cardiomyocytes in the presence of 4-hydroxytamoxifen. The cells were then subjected to flow cytometric analysis of TNNT2 and eYFP expression. ES cells without the BAC transgene were used as control. (E) Representative flow cytometric plots as well as mean ± SEM values from three independent experiments are shown. *P < 0.05; NS, not significant (Student's *t* test). (F) Schematic representation of the early CPs. CMs; cardiomyocytes, CSCs; electric conduction system cells.

The distinct genetic program of *Tbx5*-expressing CPs revealed by deep sequencing implied that *Tbx5* might directly regulate the specific properties of the FHF cells. To examine this notion, we ablated *Tbx5* in *Tbx5* ^CreERT2^/*ROSA26* ^eYFP/eYFP^ ES cells with the use of the CRISPR/Cas9 system (Fig 7A–7C) [35]. We designed a single guide (sg) RNA for the coding sequence in exon 2 immediately downstream of the first methionine in its open reading frame, so that the Cas9 nuclease does not affect the BAC transgene (the target sequence of the sgRNA was completely replaced by the *CreERT2* cassette in the transgene). The introduced mutations resulted in premature termination of translation before the T-box domain and the consequent production of short peptides unlikely to function as a transcription factor (Fig 7B and 7C, S7 and S8 Figs). Induction of cardiac differentiation in the non-modified ES cells in the presence of 4-hydroxytamoxifen similarly resulted in the differentiation of almost all eYFP^+^ cells into TNNT2^+^ cardiomyocytes (Fig 7D and 7E). In contrast, eYFP^+^ cells were not detected among the differentiated *Tbx5* knockout ES cells. These results strongly suggest the role of TBX5 in characterising the FHF by a positive feedback loop that activates directly or indirectly the earliest *Tbx5* expression. Alternatively an appropriate cell condition to transcribe this BAC transgene is lost in *Tbx5* null cells. Of note, the proportion of TNNT2^+^/eYFP^−^cardiomyocytes was similar in the knockout and non-modified ES cells, strongly suggesting that TNNT2^+^/eYFP^−^cardiomyocytes originate from the SHF CPs population. Given the previously reported phenotype of *Tbx5* null embryos, this evidence also suggests that the FHF-derived cells lost their cardiomyocyte identity in the absence of *Tbx5* expression [57]. This is also supported by the observation that no elevations of apoptotic cells are apparent when *Tbx5* is robustly induced (at differentiation day 7) during the cardiac differentiation of ES cells. This strongly suggests that *Tbx5* null cells did not die but instead lose their cardiomyocyte differentiation ability (S9 Fig). In addition, if TNNT2^+^/eYFP^−^cardiomyocytes belong to the SHF, the FHF may not be necessary for cardiomyocyte differentiation of the SHF, at least *in vitro*. Taken together, these findings suggest that a distinct genetic program related to *Tbx5* defines the unique characteristics of the FHF, fundamentally distinct from the SHF, although validating the precise function of *Tbx5* on the FHF requires further study.

## Discussion

On the basis of our results presented here, we propose a new model of CP differentiation (Fig 7F). From the EB to EHF stages, *Tbx5*-expressing cells constitute the specified cell population assigned to contribute to the FHF and therefore give rise to the left ventricle and portions of the atria. *Tbx5* ^low+^ cells appear as the initial FHF CPs and then become *Nkx2-5* ^+^/*Tbx5* ^+^ CPs. Once *Tbx5* expression is activated in the FHF, the cells are committed to a cardiomyocyte lineage only. The progenitor pool that supplies *Tbx5*-expressing FHF CPs only exists transiently from the primitive streak stage to the EHF stage, with rapid differentiation following activation of *Nkx2-5* expression. Expansion of the FHF progeny thus depends mainly on the proliferation of an already committed CP population to provide cardiomyocytes after EHF stage. On the other hand, the SHF cells become *Nkx2-5* CPs without *Tbx5* expression, which appear as an initial wave of SHF CPs after the EHF stage.

We demonstrate rapid and dynamic changes in gene expression profiles of the earliest CPs within a short time period from the Neural Plate stage to the Headfold stage; in addition to supporting the view that distinct transcriptional properties define the unique character of the FHF cells. The transcriptional program likely includes the core module of cardiac transcription factors driving differentiation [63]. Among the components of this core module, TBX5 likely plays a central role in cooperation with other cardiac genes. This is supported by our data and previous studies that indicate that *Tbx5* is necessary for the FHF’s development, even if *Tbx5* alone is insufficient for cardiomyocyte fate [57, 64]. *Tbx5* unlikely regulates progenitor function but rather plays its role to control the differentiation and patterning of the FHF. It seems that such a core module involving *Tbx5* is the reason that the FHF differs from the SHF CPs, in that they are rapidly differentiating and do not continue to function as a progenitor pool for long term supply of heart cells [65].

The mechanism underlying transcriptional regulation of *Tbx5* remains largely unclear, although enhancers sufficient to mimic all endogenous *Tbx5* expression are known to be distributed over 400 kb in the mouse genome, making it difficult to study any corresponding cis-regulatory elements [19]. Expression of the BAC transgene in the present study suggested that ~208 kb of this clone were sufficient to recapitulate *Tbx5* expression at least from the Neural Plate to the Headfold stage (S3 Fig). The expression pattern of the transgene was consistent with the results of lineage tracing using a *Tbx5* ^CreERT2^ knock-in allele [14]. In addition, our data indicate that a positive feedback loop exists to directly or indirectly activate the early *Tbx5* expression in the mouse embryo (Fig 7D and 7E), which has not been shown in previous studies [19, 63]. It is likely that this feedback loop is necessary for *Tbx5* activation at the earliest phase. Indeed, in the case of the human, the importance of a positive feedback loop via T-box binding sites in the promoter region of human *TBX5* was suggested [66]; however this human genomic region relevant to such a proposed transcriptional regulation is not conserved in the mouse genome. Thus, such a regulatory mechanism of human *TBX5* is unlikely universal across species. Full elucidation of the TBX5 binding sites is key to defining the cis-regulatory elements of mouse *Tbx5*, especially those required for the earliest expression in the cardiac lineage.

Previous studies indicated that segregation of the FHF and SHF occurs among the *Mesp1* ^*+*^ cardiac mesoderm cells from the Primitive Streak stage to the earliest phase of the Neural Plate stage [13, 14, 16]. Consistent with this, we detected the transcripts of *Nkx2-5*, *Isl1*, and *Tbx5* expression at the EB stage using single-cell analysis. Retrospective clonal tracing studies indicate that *Mesp1* ^+^ cells contributing to the FHF first appear at E6.25 and that their segregation terminates by E7.0 [13, 16]. Segregation of the SHF follows this first wave of the FHF from E6.75 and continues up to E7.5. A clone of each heart field contributes exclusively to the anatomic structures relevant to each heart field [7, 12–14, 16]. Our study entirely supports this idea, revealing that *Tbx5*-expressing CPs also showed an almost exclusive distribution with distinct transcriptional properties, and that they progressively differentiated over a relatively short period without maintaining their progenitor status. It will be of interest to determine whether the initial wave of cardiac *Mesp1* ^*+*^ nascent mesoderm cells (which correspond to the FHF but have not yet fully activated *Tbx5* expression) have sufficient plasticity to be able to change their fate according to extrinsic cues [13]. Indeed, the primitive streak or nascent mesoderm cells that naturally contribute to the future heart *in vivo* manifested the ability to change their fate after heterotopic transplantation to a new anatomic environment [5, 67]. This finding suggests that the cell fate of the FHF is not determined until the full activation of *Tbx5*.

An explanation for the biological significance of two distinct populations in heart morphogenesis can be explained by our data that supports the idea of the FHF having evolved to generate a minimal but sufficiently functional pump for initiation of blood circulation within a short time period [68–70]. The FHF may also provide the SHF with a scaffold for heart morphogenesis, but our data suggests that at least the cardiomyocyte differentiation of the SHF is independent of the FHF (Fig 7D). In line with this, our finding supports the previously demonstrated evidence that the cardiac crescent is not necessary in order for the SHF to generate the outflow tract [71]. However, we found that TNNT2 expression in eYFP^−^cardiomyocytes from the SHF, was slightly higher in the setting of *Tbx5* ablation (Fig 7D), suggesting that the differentiation of the SHF into cardiomyocytes was perhaps altered without the presence of the FHF CPs. Therefore, it is plausible that the interaction between the two heart fields control the spatiotemporal fate of SHF differentiation. Alternatively, the SHF, originally a common source for providing the pharyngeal muscle and a portion of the cardiomyocytes of the single liner heart in the protochordate *Ciona intestinalis*, may have evolved to generate additional complex heart structures for separation of the systemic and pulmonary circulation in higher animals [68–70]. Maintenance of the SHF progenitor pool is thus paramount until these evolutionarily new structures are completed. From this viewpoint, most congenital heart diseases that result from misalignment of the four chambers and great vessels might be regarded as direct or indirect diseases of the SHF.

## Supporting Information

S1 ARRIVE Checklist ARRIVE Checklist. (DOCX)

S1 Fig Distribution of NKX2-5^+^ CPs and NKX2-5^+^/TBX5^+^ CPs in EHF stage embryo. This section was derived from the same embryo indicated in Fig 1F, but the sectional plane was further posteriorly positioned as illustrated. In this section, only NKX2-5^+^ CPs (white arrow) could be found, which indicates that the area of NKX2-5^+^ cells is wider than that of TBX5 posteriorly. EN; endoderm, NE; neural ectoderm. Scale bar; 100 μm.
(TIF)

S2 Fig Dynamic Changes in the Expression Profiles of CPs from the EB to the EHF Stages. The average of RPM of each gene in each subpopulation was indicated with an error bar using the standard error. The asterisk indicates the statistical significance (*P*<0.05) when compared to the other subpopulations using a one-way ANOVA.
(TIF)

S3 Fig Generation of a BAC *Tbx5* ^CreERT2^ transgenic mouse. (A) Design of the BAC *Tbx5* ^CreERT2^ transgene. Exon 2 of *Tbx*5 in the BAC clone RP23-267B15 was replaced in-frame with the *CreERT2* cassette. (B) WISH analysis of endogenous *Tbx5* expression in wild-type (WT) embryos and of *CreERT2* expression in transgenic embryos at the indicated stages. The two expression patterns seemed identical. (C) *In situ* hybridization on sections of EHF stage embryo of *Tbx5* and *CreERT2* in BAC transgenic mouse. These are horizontal sections as indicated in the right-sided illustration. The sections arranged in parallel for *Tbx5* and *CreERT2* are sequential. Black arrows indicate the background signal, frequently observed at the margin of the tissue sections when performing *in situ* hybridization on sections. Scale bar; 100 μm. (D) E9.0 embryo stained with X-gal after the tamoxifen administration on the pregnant female at E7.5. Note only the heart was stained, suggesting that administered tamoxifen activity was optimal within 24 hours to induce the recombination of *ROSA26* ^lacZ/+^. (E) E9.0 embryo stained by X-gal after the tamoxifen treatment to the pregnant female on E8.5. Note the lateral plate mesoderm, which is most probably the forelimb bud, was stained in this case (black arrow).
(TIF)

S4 Fig The sufficient amount of expressed CreERT2 to detect in immunofluorescence. BAC *Tbx5* ^CreERT2^ transgenic embryos were subjected to the immunofluorescence analysis to detect CreERT2 protein with anti-ESR antibody, indicated by horizontal sections. The upper side of each picture is the posterior (P) and the lower side is the anterior (A). The intensity of fluorescence signal for CreERT2 (green) was relatively weaker at the EB stage than at the later stages. Al; allantois, En; endoderm, L; left, Mes; mesoderm, and NE; neural ectoderm, R; right. Scale bar; 100 μm.
(TIF)

S5 Fig Pulse-chase system via transient exposure to 4-hydroxytamoxifen. (A) Dissected BAC *Tbx5* ^CreERT2^/*ROSA26* ^lacZ/+^ embryos at E7.5 to E8.0 were cultured up to the five- or six-somite stage in the presence of 4-hydroxytamoxifen, after which 4-hydroxytamoxifen was removed (a) or not (b) and the culture was continued beyond the E8.5 equivalent (beyond the eight-somite stage). X-gal staining revealed that the future forelimb buds (arrows) are *lacZ*-negative in (a) but *lacZ*-positive in (b). Given that the forelimb bud begins to express *Tbx5* at the eight-somite stage [17] and that it takes 4 to 6 h for development from the five- to six-somite stage to the eight-somite stage (one somite is equivalent to two hours) [72], this result shows that withdrawal of 4-hydroxytamoxifen prevents further recombination at the *ROSA26* reporter allele within just a few hours. (B) Confocal micrograph on the section of the BAC *Tbx5* ^CreERT2^/*ROSA26* ^eYFP/eYFP^ embryo of the Headfold stage that was exposed to 4-hydroxytamoxifen for three hours. The inset square in the most left panel is presented in higher magnification in the right panels. Note the nuclear localization of CreERT2 recognized by anti-ESR antibody, which indicates three hours are sufficient for the translocation of CreERT2 protein into the nucleus. En; endoderm, Hf; Headfold, Head mes; head mesenchyme. Scale bar, 50 μm. (C) Confocal micrograph on the section of the BAC *Tbx5* ^CreERT2^/*ROSA26* ^eYFP/eYFP^ embryo of Headfold stage. This embryo was exposed to 4-hydroxytamoxifen for three hours, washed in HEPES-buffered DMEM three times, and then cultured continuously without 4-hydroxytamoxifen for an additional three hours. The inset square in the most left panel is presented in higher magnification to the right. Note the cytoplasmic localization of CreERT2, which indicates that three hours are enough to exclude the CreERT2 protein from the nucleus after 4-hydroxytamoxifen is withdrawn. In addition to the data presented in (A), this evidence strongly supports the notion that the recombination of reporter allele terminates within a few hours after the withdrawal of 4-hydroxytamoxifen. Scale bar, 50 μm.
(TIF)

S6 Fig Teratoma formation assay for derived BAC *Tbx5* ^CreERT2^/*ROSA26* ^eYFP/eYFP^ ES Cells. Haematoxylin-eosin staining of teratomas formed in nude mice by *Tbx5* ^CreERT2^/*ROSA26* ^eYFP/eYFP^ ES cells. The teratomas contained tissues of all three germ layers, including an epidermis-like structure (ectoderm), a gut epithelium—like structure (endoderm), and a cartilage-like structure (mesoderm), indicative of their pluripotency. Scale bar, 100 μm.
(TIF)

S7 Fig Targeting of *Tbx5* by the CRISPR/Cas9 System. Predicted translation products of the two mutated alleles of *Tbx5* are indicated along with WT TBX5. Red and blue colours in the amino acid sequence of wild-type (WT) mouse TBX5 indicate the T box and the epitope recognized by the rabbit polyclonal antibodies to TBX5, respectively, Bold letters and asterisks indicate missense and nonsense mutations, respectively.
(TIF)

S8 Fig The raw data of Western Blot for TBX5, eYFP and α-Tubulin of differentiating ES cells (related to Fig 7C). Each scanned image of the blotted membranes is indicated. The membrane used for α-Tubulin was the same membrane as used for TBX5 detection. It was subjected to the procedure to strip the already bound antibodies, and then to reprobing procedure with anti- α-Tubulin antibodies. Molecular weight, and the expected molecular weight of each protein are indicated. Red arrows indicate the band of each target protein.
(TIF)

S9 Fig Assay for apoptosis during cardiac differentiation of mouse ES cells. (A) BAC *Tbx5* ^CreERT2^ */ROSA26* ^eYFP/eYFP^ ES cells either rendered *Tbx5* null by the CRISPR/Cas9 or left unmodified (WT) were induced to differentiate into cardiomyocytes. The cells were then subjected to flow cytometric analysis of Annexin V that labels apoptotic cells on differentiation day 7. Representative example of 3 analyses is depicted. Q1 and Q2 indicate Annexin^+^/Propidium Iodide (PI)^-^ early apoptotic cells and Annexin^+^/PI^+^ late apoptotic cells, respectively. (B) Representative flow cytometric plots for all apoptotic cells as mean ± SEM values from three independent experiments are shown. No statistically significant difference was observed by Student's *t* test. NS; not significant.
(TIF)

S1 Table Primers for PCR of Marker Genes. (PDF)

S2 Table Primers and Probe Sets for Taqman Assays. (PDF)

S3 Table Number of Reads in deep sequencing on single cell cDNAs. (PDF)

S4 Table Primer Sets for Genotyping of CRISPR/Cas9–Guided Mutagenesis of *Tbx5*. (PDF)

S5 Table The enriched genes in EB *Nkx2-5* ^+^ CPs filtered via ANOVA. (PDF)

S6 Table The enriched genes in EB *Tbx5* ^+^ CPs filtered via ANOVA. (PDF)

S7 Table The enriched genes in EHF *Nkx2-5* ^+^ CPs filtered via ANOVA. (PDF)

S8 Table The enriched genes in EHF *Nkx2-5* ^+^/*Tbx5* ^*+*^ CPs filtered via ANOVA. (PDF)

S9 Table Gene Ontology enrichment analysis on *Nkx2-5* ^+^ EB CPs (*P*<0.05). (PDF)

S10 Table Gene Ontology enrichment analysis on *Tbx5* ^+^ EB CPs (*P*<0.05). (PDF)

S11 Table Gene Ontology enrichment analysis on *Nkx2-5* ^+^ FHF CPs (*P*<0.05). (PDF)

S12 Table Gene Ontology enrichment analysis on *Nkx2-5* ^+^ */Tbx5* ^+^ FHF CPs (*P*<0.05). (PDF)

## References

[1] Buckingham M, Meilhac S, Zaffran S. Building the mammalian heart from two sources of myocardial cells. Nat Rev Genet. 2005 11;6(11):826–35. . Epub 2005/11/24. eng. 16304598 10.1038/nrg1710

[2] Rana MS, Christoffels VM, Moorman AF. A molecular and genetic outline of cardiac morphogenesis. Acta Physiol (Oxf). 2013 4;207(4):588–615. . Epub 2013/01/10. eng. 23297764 10.1111/apha.12061

[3] Kinder SJ, Tsang TE, Quinlan GA, Hadjantonakis AK, Nagy A, Tam PP. The orderly allocation of mesodermal cells to the extraembryonic structures and the anteroposterior axis during gastrulation of the mouse embryo. Development. 1999 11;126(21):4691–701. . Epub 1999/10/16. eng. 10518487 10.1242/dev.126.21.4691

[4] Downs KM, Davies T. Staging of gastrulating mouse embryos by morphological landmarks in the dissecting microscope. Development. 1993 8;118(4):1255–66. . Epub 1993/08/01. eng. 8269852 10.1242/dev.118.4.1255

[5] Tam PP, Parameswaran M, Kinder SJ, Weinberger RP. The allocation of epiblast cells to the embryonic heart and other mesodermal lineages: the role of ingression and tissue movement during gastrulation. Development. 1997 5;124(9):1631–42. . Epub 1997/05/01. eng. 9165112 10.1242/dev.124.9.1631

[6] Kelly RG. The second heart field. Current topics in developmental biology. 2012;100:33–65. 10.1016/B978-0-12-387786-4.00002-6 22449840

[7] Cai CL, Liang X, Shi Y, Chu PH, Pfaff SL, Chen J, et al Isl1 identifies a cardiac progenitor population that proliferates prior to differentiation and contributes a majority of cells to the heart. Dev Cell. 2003 12;5(6):877–89. . Epub 2003/12/12. eng. 14667410 10.1016/s1534-5807(03)00363-0 PMC5578462

[8] Verzi MP, McCulley DJ, De Val S, Dodou E, Black BL. The right ventricle, outflow tract, and ventricular septum comprise a restricted expression domain within the secondary/anterior heart field. Dev Biol. 2005 11 1;287(1):134–45. . Epub 2005/09/29. eng. 16188249 10.1016/j.ydbio.2005.08.041

[9] Tzahor E, Evans SM. Pharyngeal mesoderm development during embryogenesis: implications for both heart and head myogenesis. Cardiovasc Res. 2011 7 15;91(2):196–202. Pubmed Central PMCID: 3125075. 10.1093/cvr/cvr116 21498416 PMC3125075

[10] Moretti A, Caron L, Nakano A, Lam JT, Bernshausen A, Chen Y, et al Multipotent embryonic isl1+ progenitor cells lead to cardiac, smooth muscle, and endothelial cell diversification. Cell. 2006 12 15;127(6):1151–65. . Epub 2006/11/25. eng. 17123592 10.1016/j.cell.2006.10.029

[11] Liang X, Wang G, Lin L, Lowe J, Zhang Q, Bu L, et al HCN4 dynamically marks the first heart field and conduction system precursors. Circ Res. 2013 8 2;113(4):399–407. Pubmed Central PMCID: 4017870. 10.1161/CIRCRESAHA.113.301588 23743334 PMC4017870

[12] Spater D, Abramczuk MK, Buac K, Zangi L, Stachel MW, Clarke J, et al A HCN4+ cardiomyogenic progenitor derived from the first heart field and human pluripotent stem cells. Nat Cell Biol. 2013 9;15(9):1098–106. Epub 2013/08/27. 10.1038/ncb2824 23974038

[13] Lescroart F, Chabab S, Lin X, Rulands S, Paulissen C, Rodolosse A, et al Early lineage restriction in temporally distinct populations of Mesp1 progenitors during mammalian heart development. Nat Cell Biol. 2014 9;16(9):829–40. 10.1038/ncb3024 25150979 PMC6984965

[14] Devine WP, Wythe JD, George M, Koshiba-Takeuchi K, Bruneau BG. Early patterning and specification of cardiac progenitors in gastrulating mesoderm. eLife. 2014;3 . 25296024 10.7554/eLife.03848 PMC4356145

[15] Schoenwolf GC, Garcia-Martinez V. Primitive-streak origin and state of commitment of cells of the cardiovascular system in avian and mammalian embryos. Cellular & molecular biology research. 1995;41(4):233–40. . 8775981

[16] Meilhac SM, Esner M, Kelly RG, Nicolas JF, Buckingham ME. The clonal origin of myocardial cells in different regions of the embryonic mouse heart. Dev Cell. 2004 5;6(5):685–98. . 15130493 10.1016/s1534-5807(04)00133-9

[17] Bruneau BG, Logan M, Davis N, Levi T, Tabin CJ, Seidman JG, et al Chamber-specific cardiac expression of Tbx5 and heart defects in Holt-Oram syndrome. Dev Biol. 1999 7 1;211(1):100–8. . Epub 1999/06/22. eng. 10373308 10.1006/dbio.1999.9298

[18] Xie L, Hoffmann AD, Burnicka-Turek O, Friedland-Little JM, Zhang K, Moskowitz IP. Tbx5-hedgehog molecular networks are essential in the second heart field for atrial septation. Dev Cell. 2012 8 14;23(2):280–91. Pubmed Central PMCID: 3912192. 10.1016/j.devcel.2012.06.006 22898775 PMC3912192

[19] Smemo S, Campos LC, Moskowitz IP, Krieger JE, Pereira AC, Nobrega MA. Regulatory variation in a TBX5 enhancer leads to isolated congenital heart disease. Hum Mol Genet. 2012 7 15;21(14):3255–63. Pubmed Central PMCID: 3384386. Epub 2012/05/01. 10.1093/hmg/dds165 22543974 PMC3384386

[20] Uehara M, Yashiro K, Takaoka K, Yamamoto M, Hamada H. Removal of maternal retinoic acid by embryonic CYP26 is required for correct Nodal expression during early embryonic patterning. Genes Dev. 2009 7 15;23(14):1689–98. Pubmed Central PMCID: 2714714. Epub 2009/07/17. eng. 10.1101/gad.1776209 19605690 PMC2714714

[21] Park EJ, Sun X, Nichol P, Saijoh Y, Martin JF, Moon AM. System for tamoxifen-inducible expression of cre-recombinase from the Foxa2 locus in mice. Dev Dyn. 2008 2;237(2):447–53. . Epub 2007/12/28. eng. 18161057 10.1002/dvdy.21415

[22] Soriano P. Generalized lacZ expression with the ROSA26 Cre reporter strain. Nat Genet. 1999 1;21(1):70–1. . Epub 1999/01/23. eng. 9916792 10.1038/5007

[23] Srinivas S, Watanabe T, Lin CS, William CM, Tanabe Y, Jessell TM, et al Cre reporter strains produced by targeted insertion of EYFP and ECFP into the ROSA26 locus. BMC Dev Biol. 2001;1:4 . Pubmed Central PMCID: 31338. Epub 2001/04/12. eng. 11299042 10.1186/1471-213X-1-4 PMC31338

[24] Kurimoto K, Yabuta Y, Ohinata Y, Ono Y, Uno KD, Yamada RG, et al An improved single-cell cDNA amplification method for efficient high-density oligonucleotide microarray analysis. Nucleic Acids Res. 2006;34(5):e42 . Pubmed Central PMCID: 1409679. Epub 2006/03/21. eng. 16547197 10.1093/nar/gkl050 PMC1409679

[25] Brouilette S, Kuersten S, Mein C, Bozek M, Terry A, Dias KR, et al A simple and novel method for RNA-seq library preparation of single cell cDNA analysis by hyperactive Tn5 transposase. Dev Dyn. 2012 10;241(10):1584–90. Epub 2012/08/23. eng. 10.1002/dvdy.23850 22911638

[26] Mi H, Muruganujan A, Casagrande JT, Thomas PD. Large-scale gene function analysis with the PANTHER classification system. Nat Protoc. 2013 8;8(8):1551–66. 10.1038/nprot.2013.092 23868073 PMC6519453

[27] Kanehisa M, Goto S, Sato Y, Kawashima M, Furumichi M, Tanabe M. Data, information, knowledge and principle: back to metabolism in KEGG. Nucleic Acids Res. 2014 1;42(Database issue):D199–205. Pubmed Central PMCID: 3965122. 10.1093/nar/gkt1076 24214961 PMC3965122

[28] Kawasumi A, Nakamura T, Iwai N, Yashiro K, Saijoh Y, Belo JA, et al Left-right asymmetry in the level of active Nodal protein produced in the node is translated into left-right asymmetry in the lateral plate of mouse embryos. Dev Biol. 2011 5 15;353(2):321–30. Epub 2011/03/23. eng. 10.1016/j.ydbio.2011.03.009 21419113 PMC4134472

[29] Yashiro K, Zhao X, Uehara M, Yamashita K, Nishijima M, Nishino J, et al Regulation of retinoic acid distribution is required for proximodistal patterning and outgrowth of the developing mouse limb. Dev Cell. 2004 3;6(3):411–22. . Epub 2004/03/20. eng. 15030763 10.1016/s1534-5807(04)00062-0

[30] Takaoka K, Yamamoto M, Hamada H. Origin and role of distal visceral endoderm, a group of cells that determines anterior-posterior polarity of the mouse embryo. Nat Cell Biol. 2011 7;13(7):743–52. Epub 2011/05/31. eng. 10.1038/ncb2251 21623358

[31] Kiyonari H, Kaneko M, Abe S, Aizawa S. Three inhibitors of FGF receptor, ERK, and GSK3 establishes germline-competent embryonic stem cells of C57BL/6N mouse strain with high efficiency and stability. Genesis. 2010 5;48(5):317–27. Epub 2010/02/18. eng. 10.1002/dvg.20614 20162675

[32] Wamstad JA, Alexander JM, Truty RM, Shrikumar A, Li F, Eilertson KE, et al Dynamic and coordinated epigenetic regulation of developmental transitions in the cardiac lineage. Cell. 2012 9 28;151(1):206–20. Pubmed Central PMCID: 3462286. Epub 2012/09/18. eng. 10.1016/j.cell.2012.07.035 22981692 PMC3462286

[33] Yamahara K, Fukushima S, Coppen SR, Felkin LE, Varela-Carver A, Barton PJ, et al Heterogeneic nature of adult cardiac side population cells. Biochem Biophys Res Commun. 2008 7 11;371(4):615–20. 10.1016/j.bbrc.2008.04.021 18413147

[34] Shintani Y, Kapoor A, Kaneko M, Smolenski RT, D'Acquisto F, Coppen SR, et al TLR9 mediates cellular protection by modulating energy metabolism in cardiomyocytes and neurons. Proc Natl Acad Sci U S A. 2013 3 26;110(13):5109–14. Pubmed Central PMCID: 3612600. 10.1073/pnas.1219243110 23479602 PMC3612600

[35] Cong L, Ran FA, Cox D, Lin S, Barretto R, Habib N, et al Multiplex genome engineering using CRISPR/Cas systems. Science. 2013 2 15;339(6121):819–23. Pubmed Central PMCID: 3795411. 10.1126/science.1231143 23287718 PMC3795411

[36] Kanai-Azuma M, Kanai Y, Gad JM, Tajima Y, Taya C, Kurohmaru M, et al Depletion of definitive gut endoderm in Sox17-null mutant mice. Development. 2002 5;129(10):2367–79. . Epub 2002/04/26. eng. 11973269 10.1242/dev.129.10.2367

[37] Wood HB, Episkopou V. Comparative expression of the mouse Sox1, Sox2 and Sox3 genes from pre-gastrulation to early somite stages. Mech Dev. 1999 8;86(1–2):197–201. . Epub 1999/08/14. 10446282 10.1016/s0925-4773(99)00116-1

[38] Shen MM, Wang H, Leder P. A differential display strategy identifies Cryptic, a novel EGF-related gene expressed in the axial and lateral mesoderm during mouse gastrulation. Development. 1997 1;124(2):429–42. . Epub 1997/01/01. eng. 9053319 10.1242/dev.124.2.429

[39] Harvey RP. Patterning the vertebrate heart. Nat Rev Genet. 2002 7;3(7):544–56. . 12094232 10.1038/nrg843

[40] Saga Y, Miyagawa-Tomita S, Takagi A, Kitajima S, Miyazaki J, Inoue T. MesP1 is expressed in the heart precursor cells and required for the formation of a single heart tube. Development. 1999 8;126(15):3437–47. . Epub 1999/07/07. eng. 10393122 10.1242/dev.126.15.3437

[41] O'Brien TX, Lee KJ, Chien KR. Positional specification of ventricular myosin light chain 2 expression in the primitive murine heart tube. Proc Natl Acad Sci U S A. 1993 6 1;90(11):5157–61. . Pubmed Central PMCID: 46674. Epub 1993/06/01. eng. 8506363 10.1073/pnas.90.11.5157 PMC46674

[42] Kelly RG, Brown NA, Buckingham ME. The arterial pole of the mouse heart forms from Fgf10-expressing cells in pharyngeal mesoderm. Dev Cell. 2001 9;1(3):435–40. . 11702954 10.1016/s1534-5807(01)00040-5

[43] Costello I, Pimeisl IM, Drager S, Bikoff EK, Robertson EJ, Arnold SJ. The T-box transcription factor Eomesodermin acts upstream of Mesp1 to specify cardiac mesoderm during mouse gastrulation. Nat Cell Biol. 2011 9;13(9):1084–91. Epub 2011/08/09. 10.1038/ncb2304 21822279 PMC4531310

[44] Prall OW, Menon MK, Solloway MJ, Watanabe Y, Zaffran S, Bajolle F, et al An Nkx2-5/Bmp2/Smad1 negative feedback loop controls heart progenitor specification and proliferation. Cell. 2007 3 9;128(5):947–59. . Pubmed Central PMCID: 2092439. Epub 2007/03/14. eng. 17350578 10.1016/j.cell.2007.01.042 PMC2092439

[45] Ma Q, Zhou B, Pu WT. Reassessment of Isl1 and Nkx2-5 cardiac fate maps using a Gata4-based reporter of Cre activity. Dev Biol. 2008 11 1;323(1):98–104. Pubmed Central PMCID: 2655699. Epub 2008/09/09. eng. 10.1016/j.ydbio.2008.08.013 18775691 PMC2655699

[46] Nelson DO, Jin DX, Downs KM, Kamp TJ, Lyons GE. Irx4 identifies a chamber-specific cell population that contributes to ventricular myocardium development. Dev Dyn. 2014 3;243(3):381–92. . Pubmed Central PMCID: 4005848. 24123507 10.1002/dvdy.24078 PMC4005848

[47] Cagavi E, Bartulos O, Suh CY, Sun B, Yue Z, Jiang Z, et al Functional cardiomyocytes derived from Isl1 cardiac progenitors via Bmp4 stimulation. PLoS One. 2014;9(12):e110752 Pubmed Central PMCID: 4270687. 10.1371/journal.pone.0110752 25522363 PMC4270687

[48] Mikels AJ, Nusse R. Purified Wnt5a protein activates or inhibits beta-catenin-TCF signaling depending on receptor context. PLoS biology. 2006 4;4(4):e115 . Pubmed Central PMCID: 1420652. 16602827 10.1371/journal.pbio.0040115 PMC1420652

[49] Li Y, Rankin SA, Sinner D, Kenny AP, Krieg PA, Zorn AM. Sfrp5 coordinates foregut specification and morphogenesis by antagonizing both canonical and noncanonical Wnt11 signaling. Genes Dev. 2008 11 1;22(21):3050–63. Pubmed Central PMCID: 2577796. 10.1101/gad.1687308 18981481 PMC2577796

[50] Tian Y, Cohen ED, Morrisey EE. The importance of Wnt signaling in cardiovascular development. Pediatr Cardiol. 2010 4;31(3):342–8. Pubmed Central PMCID: 3736804. 10.1007/s00246-009-9606-z 19967349 PMC3736804

[51] Naito AT, Shiojima I, Akazawa H, Hidaka K, Morisaki T, Kikuchi A, et al Developmental stage-specific biphasic roles of Wnt/beta-catenin signaling in cardiomyogenesis and hematopoiesis. Proc Natl Acad Sci U S A. 2006 12 26;103(52):19812–7. . Pubmed Central PMCID: 1750922. 17170140 10.1073/pnas.0605768103 PMC1750922

[52] Watanabe Y, Zaffran S, Kuroiwa A, Higuchi H, Ogura T, Harvey RP, et al Fibroblast growth factor 10 gene regulation in the second heart field by Tbx1, Nkx2-5, and Islet1 reveals a genetic switch for down-regulation in the myocardium. Proc Natl Acad Sci U S A. 2012 11 6;109(45):18273–80. Pubmed Central PMCID: 3494960. 10.1073/pnas.1215360109 23093675 PMC3494960

[53] McFadden DG, Barbosa AC, Richardson JA, Schneider MD, Srivastava D, Olson EN. The Hand1 and Hand2 transcription factors regulate expansion of the embryonic cardiac ventricles in a gene dosage-dependent manner. Development. 2005 1;132(1):189–201. . 15576406 10.1242/dev.01562

[54] Bamforth SD, Braganca J, Eloranta JJ, Murdoch JN, Marques FI, Kranc KR, et al Cardiac malformations, adrenal agenesis, neural crest defects and exencephaly in mice lacking Cited2, a new Tfap2 co-activator. Nat Genet. 2001 12;29(4):469–74. . 11694877 10.1038/ng768

[55] Kwon C, Qian L, Cheng P, Nigam V, Arnold J, Srivastava D. A regulatory pathway involving Notch1/beta-catenin/Isl1 determines cardiac progenitor cell fate. Nat Cell Biol. 2009 8;11(8):951–7. Pubmed Central PMCID: 2748816. 10.1038/ncb1906 19620969 PMC2748816

[56] Feil R, Wagner J, Metzger D, Chambon P. Regulation of Cre recombinase activity by mutated estrogen receptor ligand-binding domains. Biochem Biophys Res Commun. 1997 8 28;237(3):752–7. . Epub 1997/08/28. 9299439 10.1006/bbrc.1997.7124

[57] Bruneau BG, Nemer G, Schmitt JP, Charron F, Robitaille L, Caron S, et al A murine model of Holt-Oram syndrome defines roles of the T-box transcription factor Tbx5 in cardiogenesis and disease. Cell. 2001 9 21;106(6):709–21. . Epub 2001/09/27. eng. 11572777 10.1016/s0092-8674(01)00493-7

[58] Moskowitz IP, Pizard A, Patel VV, Bruneau BG, Kim JB, Kupershmidt S, et al The T-Box transcription factor Tbx5 is required for the patterning and maturation of the murine cardiac conduction system. Development. 2004 8;131(16):4107–16. . Epub 2004/08/04. eng. 15289437 10.1242/dev.01265

[59] Bakker ML, Boukens BJ, Mommersteeg MT, Brons JF, Wakker V, Moorman AF, et al Transcription factor Tbx3 is required for the specification of the atrioventricular conduction system. Circ Res. 2008 6 6;102(11):1340–9. 10.1161/CIRCRESAHA.107.169565 18467625

[60] Hoogaars WM, Tessari A, Moorman AF, de Boer PA, Hagoort J, Soufan AT, et al The transcriptional repressor Tbx3 delineates the developing central conduction system of the heart. Cardiovasc Res. 2004 6 1;62(3):489–99. . Epub 2004/05/26. eng. 15158141 10.1016/j.cardiores.2004.01.030

[61] Wiese C, Grieskamp T, Airik R, Mommersteeg MT, Gardiwal A, de Gier-de Vries C, et al Formation of the sinus node head and differentiation of sinus node myocardium are independently regulated by Tbx18 and Tbx3. Circ Res. 2009 2 13;104(3):388–97. Epub 2008/12/20. 10.1161/CIRCRESAHA.108.187062 19096026

[62] Jensen B, Wang T, Christoffels VM, Moorman AF. Evolution and development of the building plan of the vertebrate heart. Biochim Biophys Acta. 2013 4;1833(4):783–94. 10.1016/j.bbamcr.2012.10.004 23063530

[63] He A, Kong SW, Ma Q, Pu WT. Co-occupancy by multiple cardiac transcription factors identifies transcriptional enhancers active in heart. Proc Natl Acad Sci U S A. 2011 4 5;108(14):5632–7. Pubmed Central PMCID: 3078411. 10.1073/pnas.1016959108 21415370 PMC3078411

[64] Takeuchi JK, Bruneau BG. Directed transdifferentiation of mouse mesoderm to heart tissue by defined factors. Nature. 2009 6 4;459(7247):708–11. Pubmed Central PMCID: 2728356. Epub 2009/04/28. eng. 10.1038/nature08039 19396158 PMC2728356

[65] Fijnvandraat AC, Lekanne Deprez RH, Christoffels VM, Ruijter JM, Moorman AF. TBX5 overexpression stimulates differentiation of chamber myocardium in P19C16 embryonic carcinoma cells. Journal of muscle research and cell motility. 2003;24(2–3):211–8. . 14609032 10.1023/a:1026063409656

[66] Sun G, Lewis LE, Huang X, Nguyen Q, Price C, Huang T. TBX5, a gene mutated in Holt-Oram syndrome, is regulated through a GC box and T-box binding elements (TBEs). J Cell Biochem. 2004 5 1;92(1):189–99. . 15095414 10.1002/jcb.20039

[67] Inagaki T, Garcia-Martinez V, Schoenwolf GC. Regulative ability of the prospective cardiogenic and vasculogenic areas of the primitive streak during avian gastrulation. Dev Dyn. 1993 5;197(1):57–68. . 8400411 10.1002/aja.1001970106

[68] Moorman AF, Christoffels VM, Anderson RH, van den Hoff MJ. The heart-forming fields: one or multiple? Philos Trans R Soc Lond B Biol Sci. 2007 8 29;362(1484):1257–65. . Pubmed Central PMCID: 2440394. 17581808 10.1098/rstb.2007.2113 PMC2440394

[69] Wang W, Razy-Krajka F, Siu E, Ketcham A, Christiaen L. NK4 antagonizes Tbx1/10 to promote cardiac versus pharyngeal muscle fate in the ascidian second heart field. PLoS biology. 2013 12;11(12):e1001725 Pubmed Central PMCID: 3849182. 10.1371/journal.pbio.1001725 24311985 PMC3849182

[70] Stolfi A, Gainous TB, Young JJ, Mori A, Levine M, Christiaen L. Early chordate origins of the vertebrate second heart field. Science. 2010 7 30;329(5991):565–8. Epub 2010/07/31. 10.1126/science.1190181 20671188 PMC4970750

[71] Mjaatvedt CH, Nakaoka T, Moreno-Rodriguez R, Norris RA, Kern MJ, Eisenberg CA, et al The outflow tract of the heart is recruited from a novel heart-forming field. Dev Biol. 2001 10 1;238(1):97–109. . 11783996 10.1006/dbio.2001.0409

[72] Forsberg H, Crozet F, Brown NA. Waves of mouse Lunatic fringe expression, in four-hour cycles at two-hour intervals, precede somite boundary formation. Current biology: CB. 1998 9 10;8(18):1027–30. . Epub 1998/09/19. 9740806 10.1016/s0960-9822(07)00424-1

