# Supplementary material for: Single-Cell Expression Profiling Reveals a Dynamic State of Cardiac Precursor Cells in the Early Mouse Embryo
Source: PLoS One. 2015 Oct 15;10(10):e0140831. doi: 10.1371/journal.pone.0140831 (PMC4607431; doi:10.1371/journal.pone.0140831)

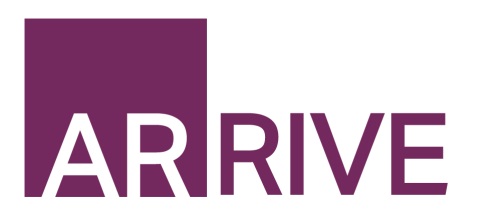


The ARRIVE Guidelines Checklist

Animal Research: Reporting In Vivo Experiments

Carol Kilkenny^1^, William J Browne^2^, Innes C Cuthill^3^, Michael Emerson^4^ and Douglas G Altman^5^

*^1^The National Centre for the Replacement, Refinement and Reduction of Animals in Research, London, UK, ^2^School of Veterinary Science, University of Bristol, Bristol, UK, ^3^School of Biological Sciences, University of Bristol, Bristol, UK, ^4^National Heart and Lung Institute, Imperial College London, UK, ^5^Centre for Statistics in Medicine, University of Oxford, Oxford, UK.*

|  | | ITEM | RECOMMENDATION | Section/ Paragraph |
| --- | --- | --- | --- | --- |
| 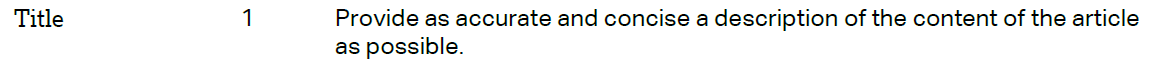 | | | **Title page**  Page 1 |  |
| 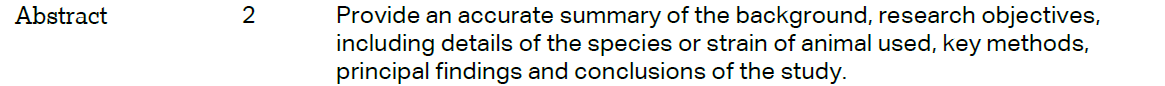 | | | **Abstract**  Page 2 |  |
| INTRODUCTION | | |  |  |
| 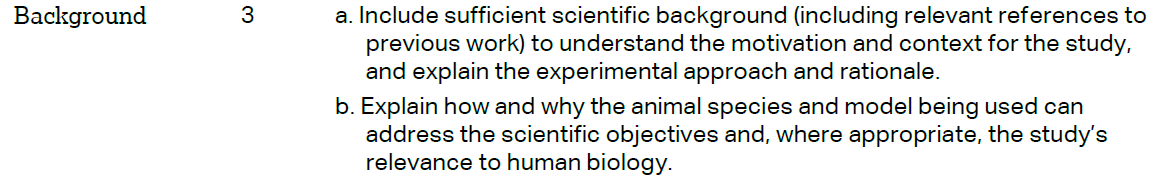 | | | **Introduction**  Page 3-5 |  |
| 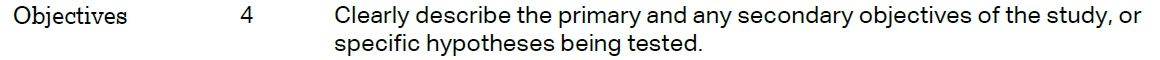 | | | **Introduction**  Page 3-5 |  |
| METHODS | | |  |  |
| 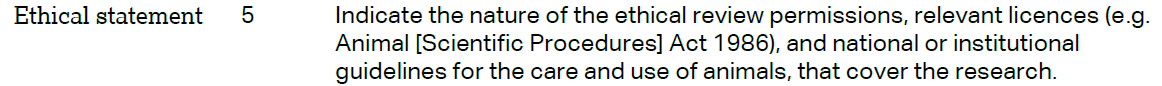 | | | **Material & Methods**  Page 6-12 |  |
| 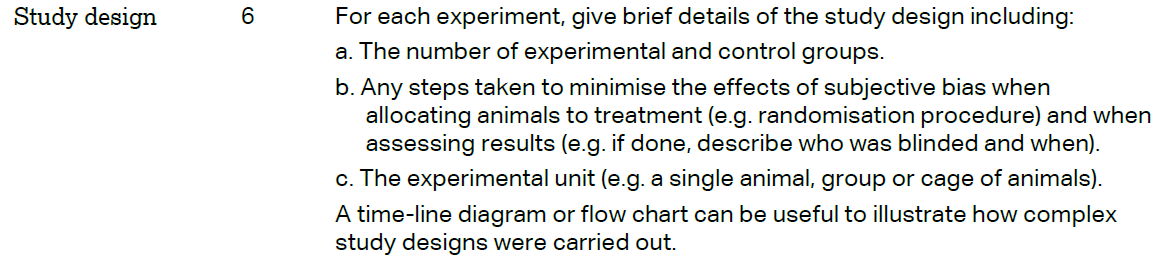 | | | **Material & Methods**  **Results**  Page 6-22 |  |
| 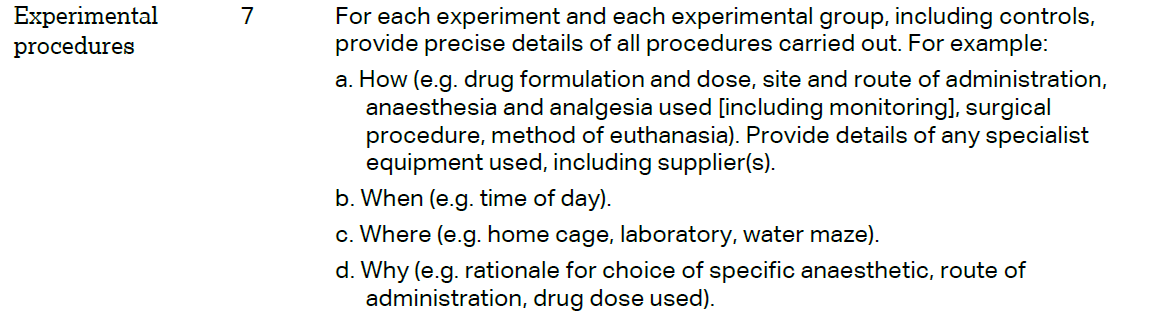 | | | **Material & Methods**  **Results**  Page 6-22 |  |
| 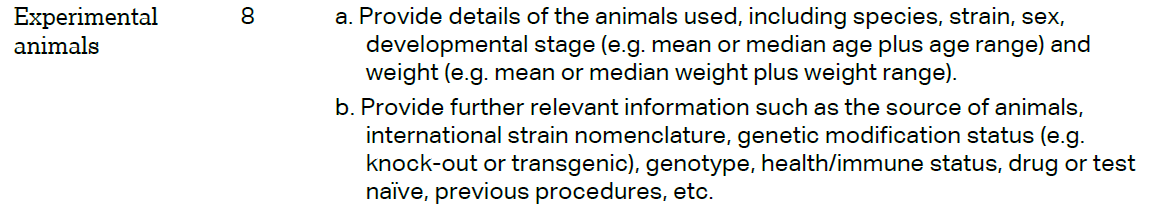 | | | **Material & Methods**  **Results**  Page 6-22 |  |

The ARRIVE guidelines. Originally published in *PLoS Biology*, June 2010^1^

| 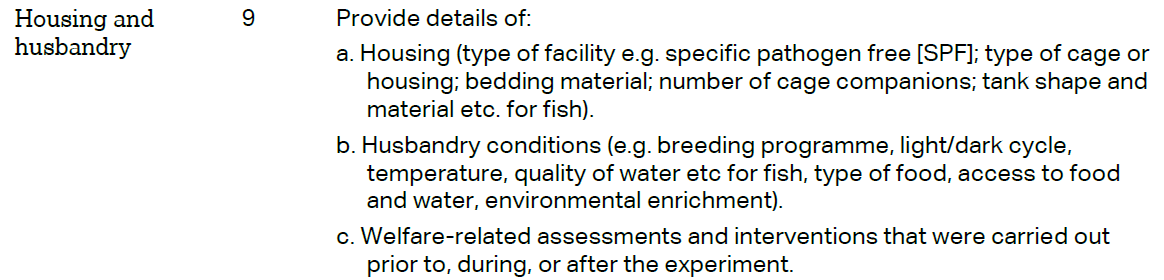 | **Material & Methods**  Page 6-13 | |
| --- | --- | --- |
| 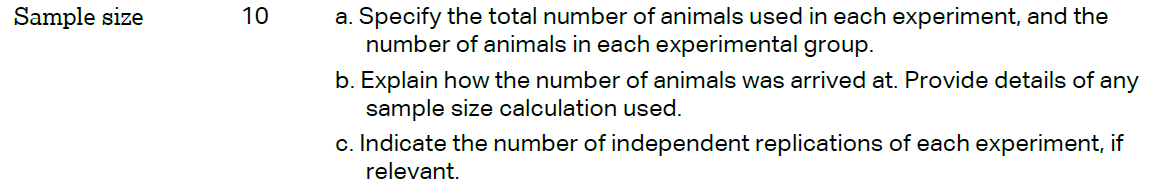 | **Material & Methods**  **Results**  Page 6-22 | |
| 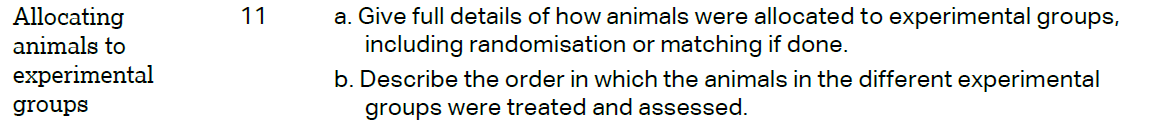 | **Material & Methods**  **Results**  Page 6-22 | |
| 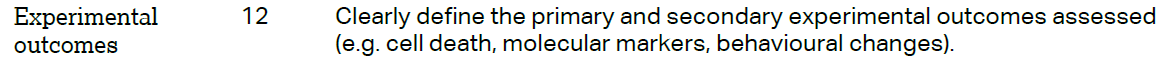 | **Results**  Page 14-22 | |
| 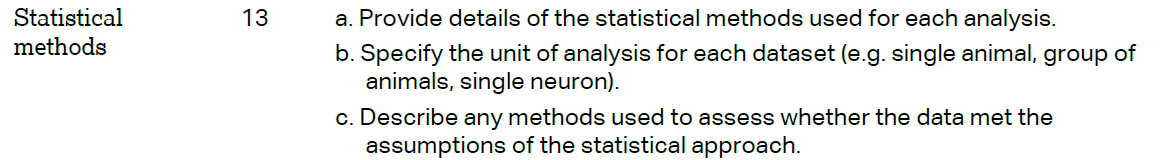 | **Material & Methods**  **Results**  Page 6-22 | |
| RESULTS |  | |
| 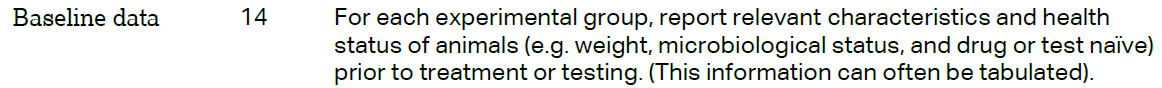 | **Material & Methods**  **Results**  Page 6-22 | |
| 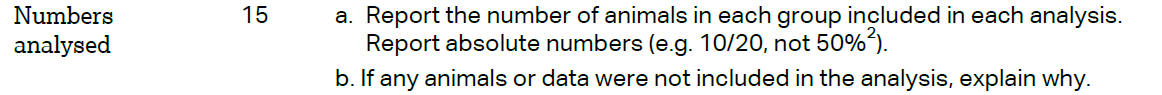 | **Results**  Page 6-22 | |
| 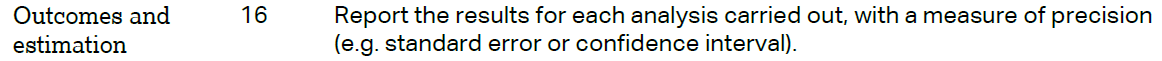 | **Results**  Page 6-22 | |
| 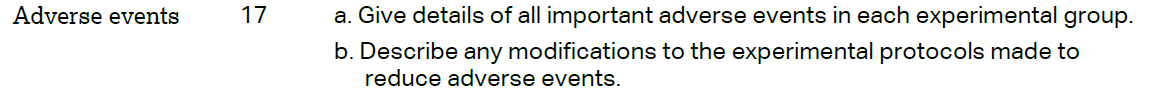 | **Results**  Page 6-22 | |
| DISCUSSION |  | |
| 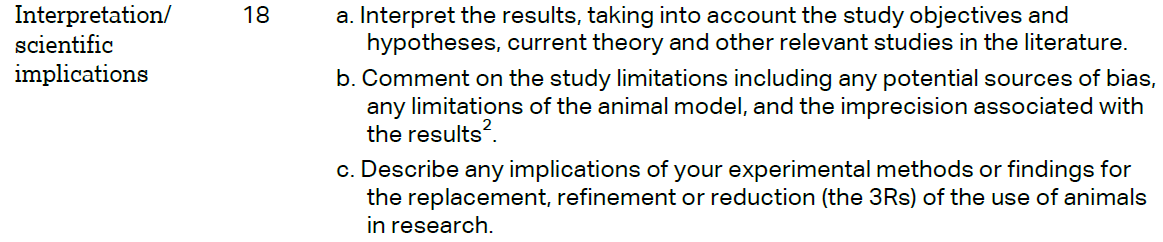 | **Results**  **Discussion**  Page 14-26 | |
| 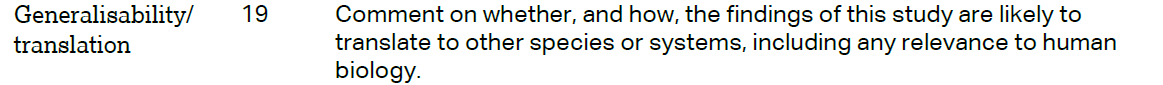 | **Discussion**  Page 23-26 | |
| 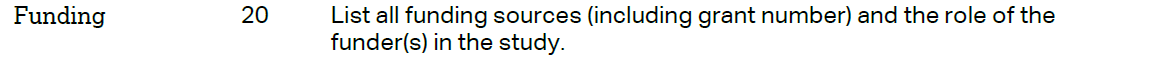 | | **Funding Information** |


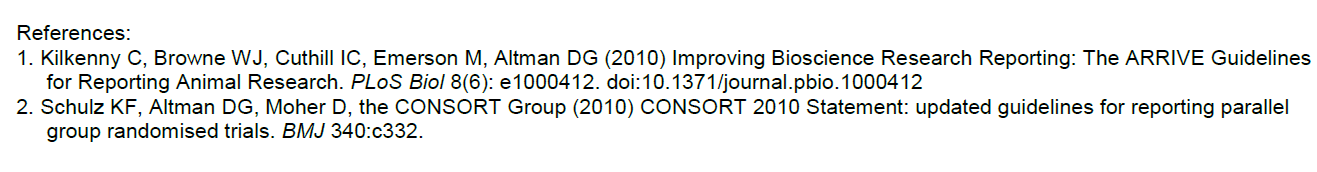

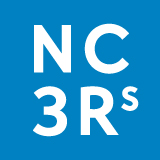

Supplement: S1 ARRIVE Checklist — (DOCX) [file pone.0140831.s001.docx]
