# Supplementary figures and images for: Single-Cell Expression Profiling Reveals a Dynamic State of Cardiac Precursor Cells in the Early Mouse Embryo
Source: PLoS One. 2015 Oct 15;10(10):e0140831. doi: 10.1371/journal.pone.0140831 (PMC4607431; doi:10.1371/journal.pone.0140831)

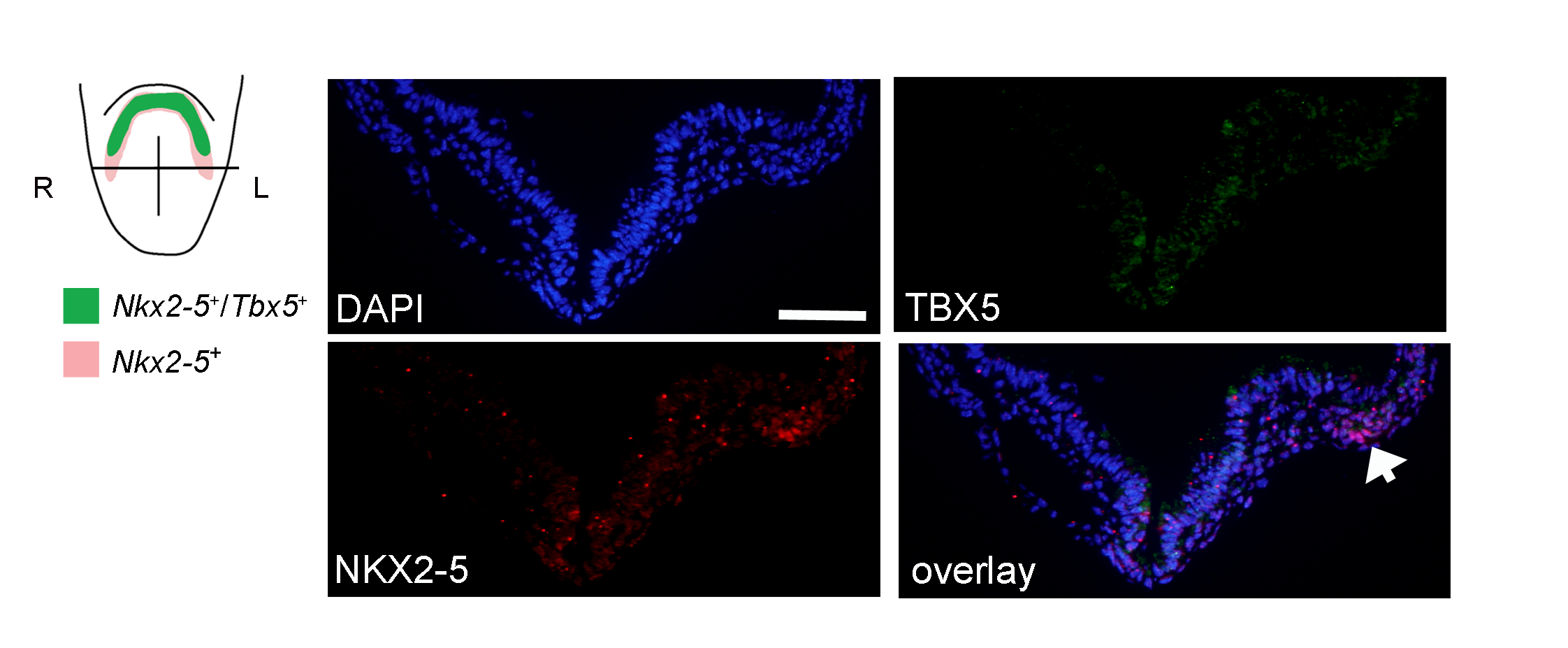

Supplement: S1 Fig — This section was derived from the same embryo indicated in Fig 1F, but the sectional plane was further posteriorly positioned as illustrated. In this section, only NKX2-5+ CPs (white arrow) could be found, which indicates that the area of NKX2-5+ cells is wider than that of TBX5 posteriorly. EN; endoderm, NE; neural ectoderm. Scale bar; 100 μm. (TIF) [file pone.0140831.s002.tif]

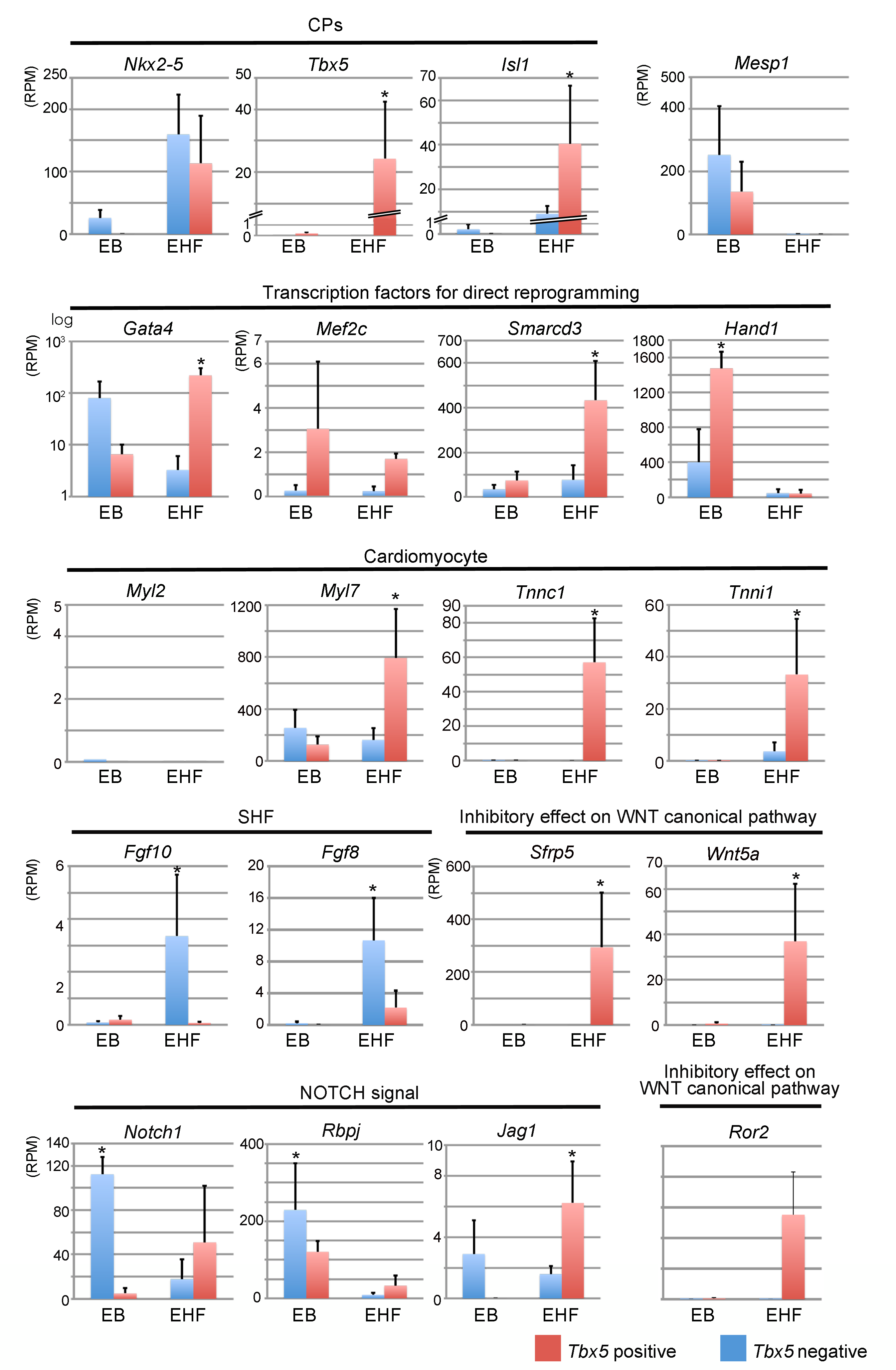

Supplement: S2 Fig — The average of RPM of each gene in each subpopulation was indicated with an error bar using the standard error. The asterisk indicates the statistical significance (P<0.05) when compared to the other subpopulations using a one-way ANOVA. (TIF) [file pone.0140831.s003.tif]

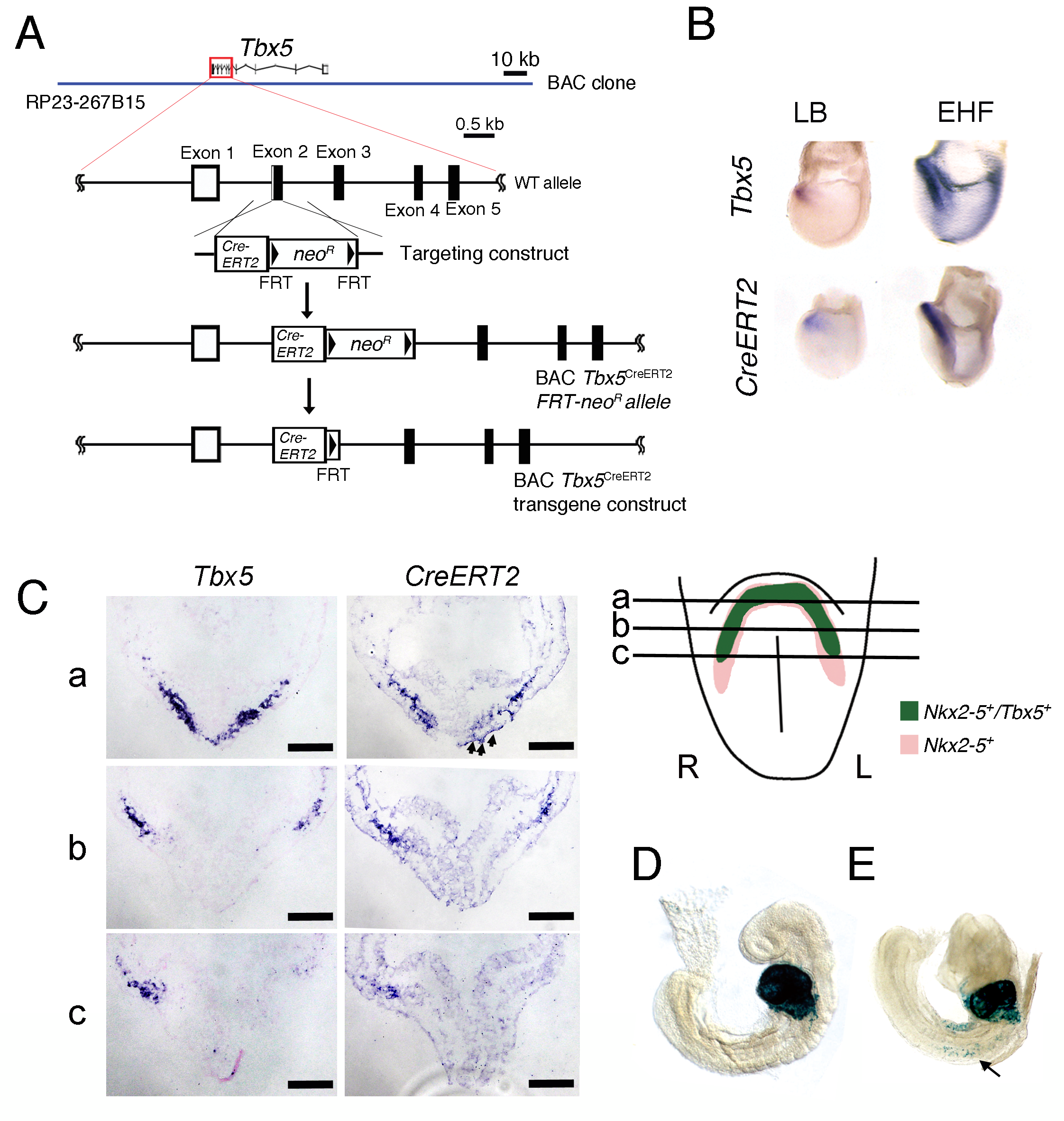

Supplement: S3 Fig — (A) Design of the BAC Tbx5 CreERT2 transgene. Exon 2 of Tbx5 in the BAC clone RP23-267B15 was replaced in-frame with the CreERT2 cassette. (B) WISH analysis of endogenous Tbx5 expression in wild-type (WT) embryos and of CreERT2 expression in transgenic embryos at the indicated stages. The two expression patterns seemed identical. (C) In situ hybridization on sections of EHF stage embryo of Tbx5 and CreERT2 in BAC transgenic mouse. These are horizontal sections as indicated in the right-sided illustration. The sections arranged in parallel for Tbx5 and CreERT2 are sequential. Black arrows indicate the background signal, frequently observed at the margin of the tissue sections when performing in situ hybridization on sections. Scale bar; 100 μm. (D) E9.0 embryo stained with X-gal after the tamoxifen administration on the pregnant female at E7.5. Note only the heart was stained, suggesting that administered tamoxifen activity was optimal within 24 hours to induce the recombination of ROSA26 lacZ/+. (E) E9.0 embryo stained by X-gal after the tamoxifen treatment to the pregnant female on E8.5. Note the lateral plate mesoderm, which is most probably the forelimb bud, was stained in this case (black arrow). (TIF) [file pone.0140831.s004.tif]

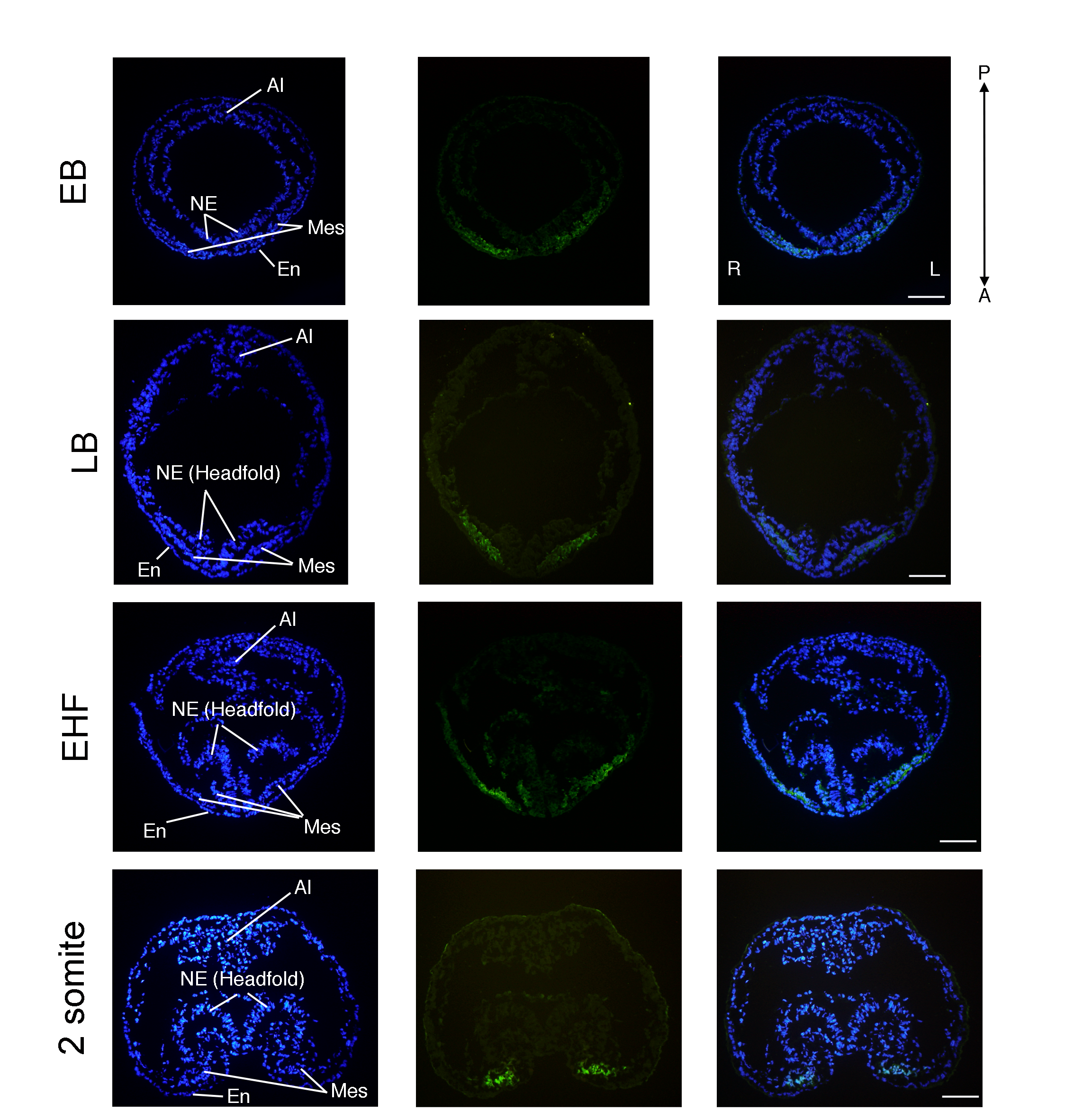

Supplement: S4 Fig — BAC Tbx5 CreERT2 transgenic embryos were subjected to the immunofluorescence analysis to detect CreERT2 protein with anti-ESR antibody, indicated by horizontal sections. The upper side of each picture is the posterior (P) and the lower side is the anterior (A). The intensity of fluorescence signal for CreERT2 (green) was relatively weaker at the EB stage than at the later stages. Al; allantois, En; endoderm, L; left, Mes; mesoderm, and NE; neural ectoderm, R; right. Scale bar; 100 μm. (TIF) [file pone.0140831.s005.tif]

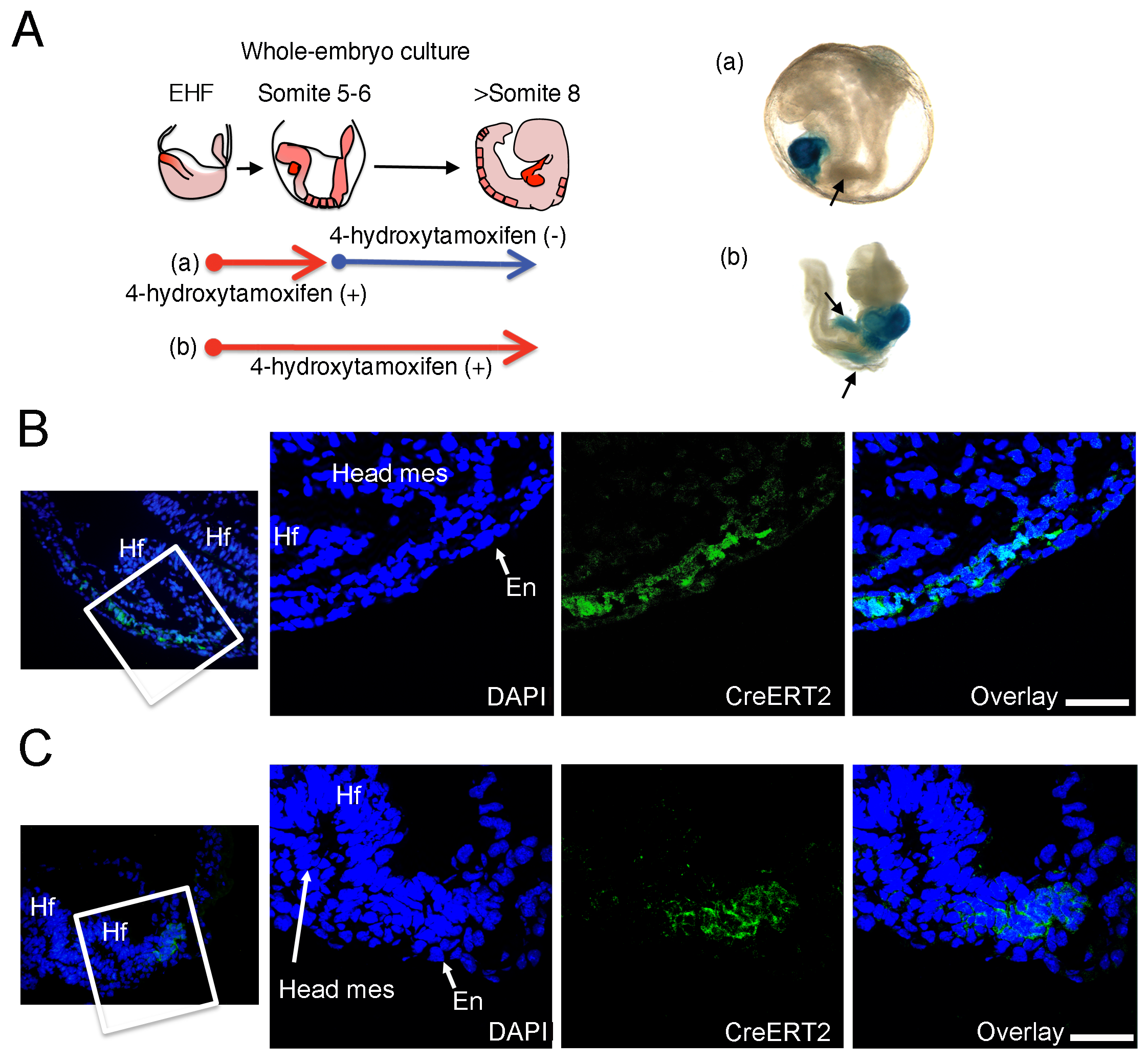

Supplement: S5 Fig — (A) Dissected BAC Tbx5 CreERT2/ROSA26 lacZ/+ embryos at E7.5 to E8.0 were cultured up to the five- or six-somite stage in the presence of 4-hydroxytamoxifen, after which 4-hydroxytamoxifen was removed (a) or not (b) and the culture was continued beyond the E8.5 equivalent (beyond the eight-somite stage). X-gal staining revealed that the future forelimb buds (arrows) are lacZ-negative in (a) but lacZ-positive in (b). Given that the forelimb bud begins to express Tbx5 at the eight-somite stage [17] and that it takes 4 to 6 h for development from the five- to six-somite stage to the eight-somite stage (one somite is equivalent to two hours) [72], this result shows that withdrawal of 4-hydroxytamoxifen prevents further recombination at the ROSA26 reporter allele within just a few hours. (B) Confocal micrograph on the section of the BAC Tbx5 CreERT2/ROSA26 eYFP/eYFP embryo of the Headfold stage that was exposed to 4-hydroxytamoxifen for three hours. The inset square in the most left panel is presented in higher magnification in the right panels. Note the nuclear localization of CreERT2 recognized by anti-ESR antibody, which indicates three hours are sufficient for the translocation of CreERT2 protein into the nucleus. En; endoderm, Hf; Headfold, Head mes; head mesenchyme. Scale bar, 50 μm. (C) Confocal micrograph on the section of the BAC Tbx5 CreERT2/ROSA26 eYFP/eYFP embryo of Headfold stage. This embryo was exposed to 4-hydroxytamoxifen for three hours, washed in HEPES-buffered DMEM three times, and then cultured continuously without 4-hydroxytamoxifen for an additional three hours. The inset square in the most left panel is presented in higher magnification to the right. Note the cytoplasmic localization of CreERT2, which indicates that three hours are enough to exclude the CreERT2 protein from the nucleus after 4-hydroxytamoxifen is withdrawn. In addition to the data presented in (A), this evidence strongly supports the notion that the recombination of reporter allel [file pone.0140831.s006.tif]

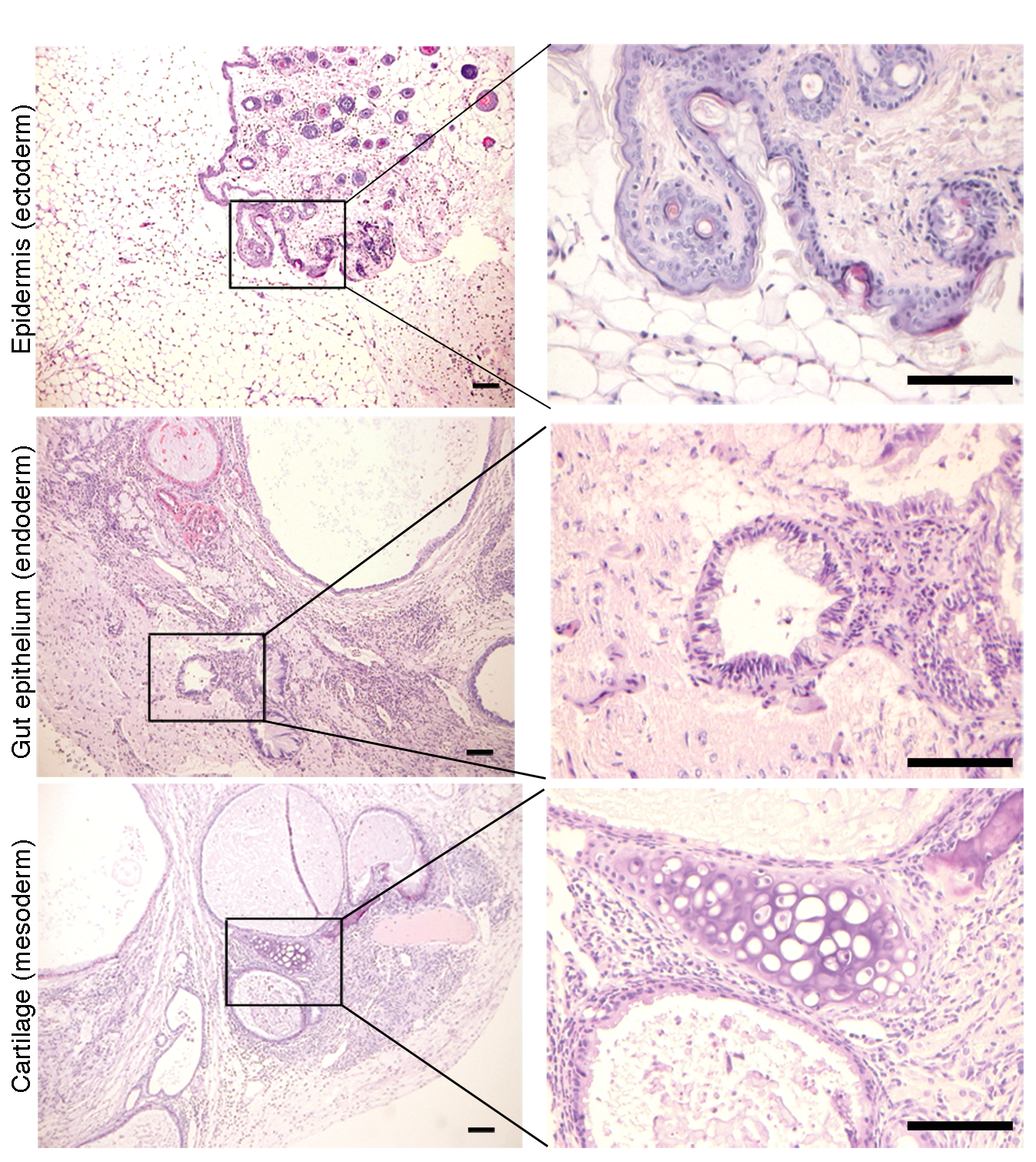

Supplement: S6 Fig — Haematoxylin-eosin staining of teratomas formed in nude mice by Tbx5 CreERT2/ROSA26 eYFP/eYFP ES cells. The teratomas contained tissues of all three germ layers, including an epidermis-like structure (ectoderm), a gut epithelium—like structure (endoderm), and a cartilage-like structure (mesoderm), indicative of their pluripotency. Scale bar, 100 μm. (TIF) [file pone.0140831.s007.tif]

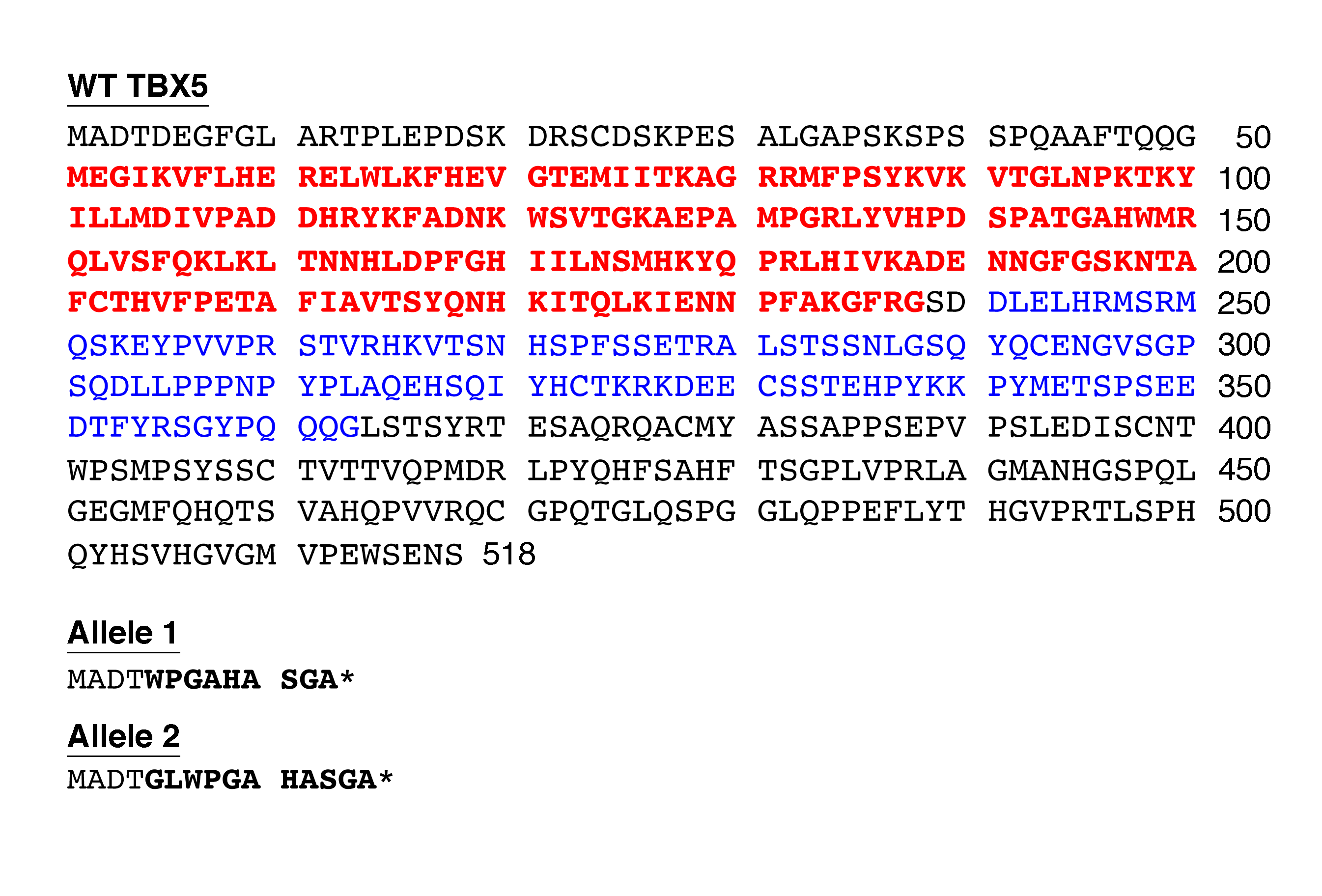

Supplement: S7 Fig — Predicted translation products of the two mutated alleles of Tbx5 are indicated along with WT TBX5. Red and blue colours in the amino acid sequence of wild-type (WT) mouse TBX5 indicate the T box and the epitope recognized by the rabbit polyclonal antibodies to TBX5, respectively, Bold letters and asterisks indicate missense and nonsense mutations, respectively. (TIF) [file pone.0140831.s008.tif]

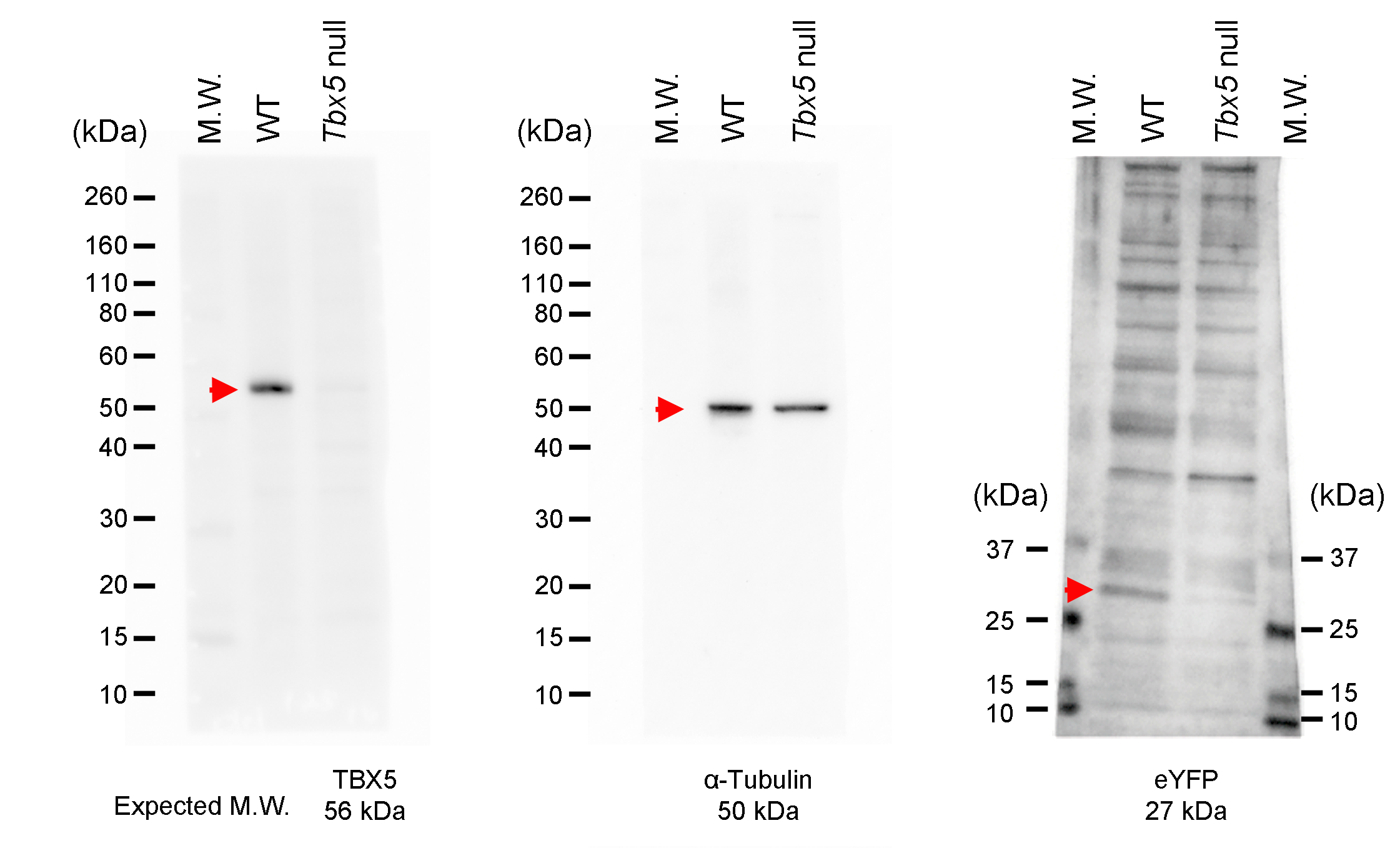

Supplement: S8 Fig — Each scanned image of the blotted membranes is indicated. The membrane used for α-Tubulin was the same membrane as used for TBX5 detection. It was subjected to the procedure to strip the already bound antibodies, and then to reprobing procedure with anti- α-Tubulin antibodies. Molecular weight, and the expected molecular weight of each protein are indicated. Red arrows indicate the band of each target protein. (TIF) [file pone.0140831.s009.tif]

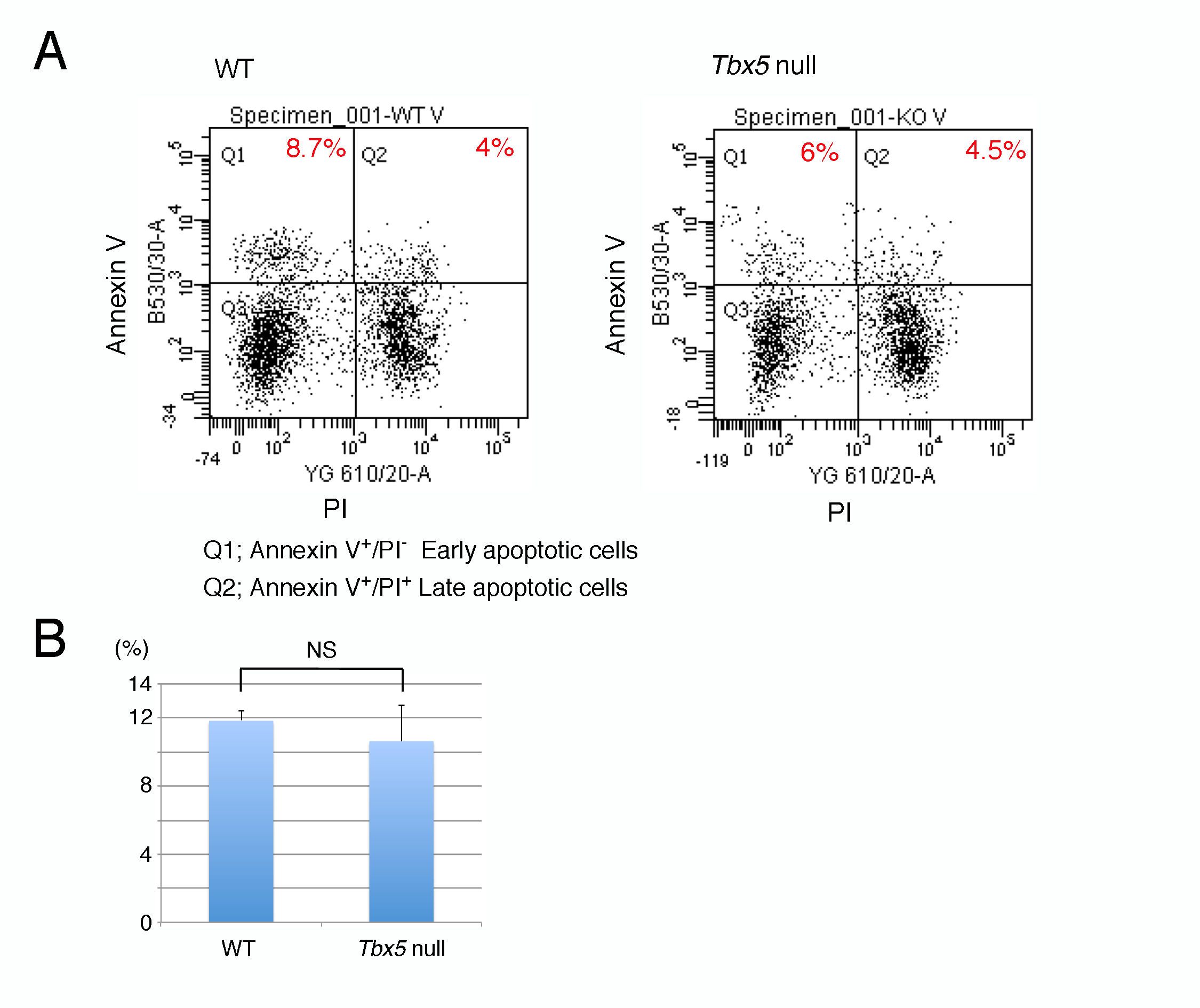

Supplement: S9 Fig — (A) BAC Tbx5 CreERT2 /ROSA26 eYFP/eYFP ES cells either rendered Tbx5 null by the CRISPR/Cas9 or left unmodified (WT) were induced to differentiate into cardiomyocytes. The cells were then subjected to flow cytometric analysis of Annexin V that labels apoptotic cells on differentiation day 7. Representative example of 3 analyses is depicted. Q1 and Q2 indicate Annexin+/Propidium Iodide (PI)- early apoptotic cells and Annexin+/PI+ late apoptotic cells, respectively. (B) Representative flow cytometric plots for all apoptotic cells as mean ± SEM values from three independent experiments are shown. No statistically significant difference was observed by Student's t test. NS; not significant. (TIF) [file pone.0140831.s010.tif]
