# Supplementary material for: Single-Cell Expression Profiling Reveals a Dynamic State of Cardiac Precursor Cells in the Early Mouse Embryo
Source: PLoS One. 2015 Oct 15;10(10):e0140831. doi: 10.1371/journal.pone.0140831 (PMC4607431; doi:10.1371/journal.pone.0140831)
Supplement: S1 Table — (PDF) [file pone.0140831.s011.pdf]

Table S1. Primers for PCR of Marker Genes

Gene	Primer name	Primer sequence (5' to 3')
Gapdh	Gapdh_L	GCATCTTGGGCTACACTGAG
	Gapdh_R	AGGTGGAAGAGTGGGAGTTG
Nkx2-5	Nkx2-5_L	TTGACGTAGCCTGGTGTCTC
	Nkx2-5_R	TAGTGTGGAATCCGTCGAAA
Tbx5	Tbx5_L	CGTCGTGGAATTCAGAGTTG
	Tbx5_R	CGATAGGTGCTGAGGAGTGA
Isl1	Isl1_L	TTATAAAGCATTGCAACAAGGTT
	Isl1_R	TCTTGGACAGACAGGAGTCAA
Mesp1	Mesp1_L	ATTGTCACCCTGTCTGAGCA
	Mesp1_R	GATGCCCATGTTGGTATCAC
Cfc1 *(Cryptic)*	Cfc1_L	CGCCAGAGGATCAAGAGAAT
	Cfc1_R	CTTAGGAGCCTCAGCCCTTA
Myl2	Mlc2v_R	AGGGTCACTGAAGGCTGACT

(Mlc2v)	Mlc2v_R	GGTCGATCTCCTCTTTGGAG
Myl7 *(Mlc2a)*	Mlc2a_L	TCAAGCAGCTTCTCATGACC
	Mlc2a_R	AGCGCAAACAGTTGCTCTAC
Actc1 (cardiac actin)	Actc1_L	CCCATCTCTCATCAGTCATTGT
	Actc1_R	TGTAGGTTGCAAGTCCTGGT
Sox2	Sox2_L	CATGAGAGCAAGTACTGGCAAG
	Sox2_R	CCAACGATATCAACCTGCATGG
Sox17	Sox17_L	TTCTGTACACTTTAATGAGGCTGTTC
	Sox17_R	TTGTGGGAAGTGGGATCAAG
Cre	Cre_L	AGGTTCGTTCACTCATGGA
	Cre_R	TCGACCAGTTTAGTTACCC
Oct3/4	Oct3/4_L	GAAGAGTATGAGGCTACAGGG
	Oct3/4_R	CCCTGTAGCCTCATACTCTTC
Sry	Sry_L	CGGGATCCATGTCAAGCGCCCCATGAATGCATTTATG

	Sry_R	GCGGAATTCACTTTAGCCCTCCGATGAGGCTGAT<br>AT
