# Supplementary material for: Single-Cell Expression Profiling Reveals a Dynamic State of Cardiac Precursor Cells in the Early Mouse Embryo
Source: PLoS One. 2015 Oct 15;10(10):e0140831. doi: 10.1371/journal.pone.0140831 (PMC4607431; doi:10.1371/journal.pone.0140831)
Supplement: S2 Table — (PDF) [file pone.0140831.s012.pdf]

Table S2. Primers and Probe Sets for Taqman Assays

Gene	Name	Sequence (5’ to 3’)
Nkx2-5	Nkx2-5_L	TTGACGTAGCCTGGTGTCTC
	Nkx2-5_R	TAGTGTGGAATCCGTCGAAA
	Nkx2-5_Probe	CTGGCAGAGCCCTTCCAGGG
Tbx5	Tbx5_L	CGTCGTGGAATTCAGAGTTG
	Tbx5_R	CGATAGGTGCTGAGGAGTGA
	Tbx5_Probe	TGTCGGGCTCAGTCTGACACCC
