# Supplementary material for: Single-Cell Expression Profiling Reveals a Dynamic State of Cardiac Precursor Cells in the Early Mouse Embryo
Source: PLoS One. 2015 Oct 15;10(10):e0140831. doi: 10.1371/journal.pone.0140831 (PMC4607431; doi:10.1371/journal.pone.0140831)
Supplement: S3 Table — (PDF) [file pone.0140831.s013.pdf]

Table S3. Number of Reads in deep sequencing

Sample ID	Total Number of Reads
EB *Tbx5*_1	36,733,874
EB *Tbx5*_2	36,168,502
EB *Tbx5*_3	32,844,430
EB *Nkx2-5*_1	27,764,442
EB *Nkx2-5*_2	22,681,504
EB *Nkx2-5*_3	35,891,446
EHF *Nkx2-5/Tbx5*_1	37,905,007
EHF *Nkx2-5/Tbx5*_2	36,588,366
EHF *Nkx2-5/Tbx5*_3	34,866,324
EHF *Nkx2-5*_1	33,505,486
EHF *Nkx2-5*_2	35,445,587
EHF *Nkx2-5*_3	12,475,588
