# Supplementary material for: Single-Cell Expression Profiling Reveals a Dynamic State of Cardiac Precursor Cells in the Early Mouse Embryo
Source: PLoS One. 2015 Oct 15;10(10):e0140831. doi: 10.1371/journal.pone.0140831 (PMC4607431; doi:10.1371/journal.pone.0140831)
Supplement: S4 Table — (PDF) [file pone.0140831.s014.pdf]

Table S4. Primer Sets for Genotyping of CRISPR/Cas9–Guided Mutagenesis of *Tbx5*

Primer		Sequence (5’ to 3’)
For PCR	Forward	GACAAATTTGACGGACAGCTC
	Reverse	CCATAAAGATAAACGCCTCGAT
For sequencing	Forward	TCCAACTCCCTCTCTTGTCC
	Reverse	CCTGCACCGTTAGCAGAGAG
