# Supplementary material for: Single-Cell Expression Profiling Reveals a Dynamic State of Cardiac Precursor Cells in the Early Mouse Embryo
Source: PLoS One. 2015 Oct 15;10(10):e0140831. doi: 10.1371/journal.pone.0140831 (PMC4607431; doi:10.1371/journal.pone.0140831)
Supplement: S5 Table — (PDF) [file pone.0140831.s015.pdf]

Table S5. The enriched genes in EB *Nkx2-5*+ CPs filtered via ANOVA

ID	p-value (Attribute)	p-value	Fold-Change	F (Attribute)	SS (Attribute)	SS (Error)	F (Error)
Ctnnb1	0.140921	0.0321114	8.99E+307	2.42352	3.44E+06	3.79E+06	1
Pabpc1	0.150959	0.039267	8.99E+307	2.3278	5.39E+06	6.18E+06	1
Tcfl5	4.97E-05	5.96E-06	9.84E+189	36.8302	901195	65250.5	1
Zfp207	0.118229	0.0331035	4.30E+187	2.67465	1.06E+06	1.06E+06	1
Ybx3	0.0801189	0.0365882	5.49E+155	3.2689	940291	767059	1
Odc1	0.00537638	0.0149446	1.11E+151	9.36997	1.67E+06	475382	1
Arpc5	0.022016	0.00384162	2.00E+144	5.68921	546063	255953	1
Ctdsp2	0.0540119	0.0149998	1.78E+138	3.92943	587769	398883	1
Wdr45b	0.0920342	0.0186365	3.56E+134	3.05094	475163	415315	1
Ctbp2	0.170389	0.0358581	2.81E+120	2.16288	368321	454112	1
Strap	0.0125305	0.00184897	3.93E+110	7.00912	307054	116821	1
Bzw2	0.193178	0.0404416	1.60E+108	1.99647	291994	390013	1
Lamp1	0.196628	0.0464344	8.59E+107	1.97337	309185	417811	1
Enoph1	0.0844814	0.0362607	1.08E+105	3.18471	414739	347275	1
Clns1a	0.137082	0.0271758	3.27E+101	2.46237	259741	281291	1
Mex3b	0.141333	0.0276591	7.85E+100	2.41943	254251	280232	1
Jam3	0.202801	0.0499175	1.12E+99	1.93323	265441	366145	1
Dpysl5	0.0459448	0.00765925	4.34E+97	4.21899	239545	151407	1
Cisd2	0.0967698	0.0208549	4.16E+93	2.97379	235834	211477	1
Sec31a	0.0601228	0.0135208	4.52E+90	3.74368	230410	164123	1
Emb	0.169525	0.0445799	1.30E+89	2.16972	226683	278602	1
Sec13	0.0687049	0.013155	5.10E+88	3.51901	205003	155349	1
Myl12a	0.165044	0.0411553	8.48E+86	2.20586	210142	254041	1
Lsm8	0.00701224	0.00522424	2.54E+86	8.57486	329940	102607	1
Asap1	0.0853231	0.0153053	1.43E+86	3.16909	185687	156249	1
Ywhaq	0.0856004	0.020502	3.27E+84	3.16398	202910	171017	1
Pitpnb	0.118026	0.0281748	1.45E+84	2.67717	197500	196725	1
Srrm1	0.146247	0.0378504	5.75E+83	2.37173	200805	225776	1
Cdca7	0.0417324	0.00747761	4.46E+80	4.39656	168743	102349	1
Brk1	0.0467278	0.00764275	3.63E+74	4.18821	138552	88217.5	1
Cry1	0.107201	0.0200124	4.05E+73	2.81917	135656	128318	1
Smad4	0.0108351	0.0316936	4.22E+71	7.38039	417700	150922	1
Papss1	0.111725	0.0293694	8.02E+70	2.75776	147336	142470	1
Stk4	0.00726592	0.00129617	2.36E+70	8.4724	133689	42078.4	1
Prpf4	0.200313	0.0423416	4.77E+67	1.94922	114263	156320	1
Sall4	0.128634	0.0345451	1.79E+67	2.55276	132987	138921	1
Dnajc10	0.0824371	0.0178688	1.43E+67	3.22348	122751	101547	1
Slc30a5	0.016965	0.00263199	1.13E+67	6.27684	113934	48403.8	1
Tcf3	0.0631954	0.020666	4.85E+66	3.65892	146619	106858	1
Zc3hc1	0.120293	0.0301811	2.97E+66	2.64945	126077	126896	1
Cyb5	0.0652128	0.0190633	2.53E+66	3.60599	138173	102180	1
Ube2e3	0.0146731	0.00231396	2.19E+66	6.6208	112565	45338	1

Exo1	0.0624469	0.0153663	9.54E+65	3.67909	126645	91794.7	1
Sdc1	0.199652	0.0418217	2.58E+64	1.95351	103049	140668	1
Taf4a	0.128535	0.0382901	1.01E+59	2.55386	107947	112715	1
Psmc2	0.0836652	0.0353887	7.82E+58	3.20005	129410	107840	1
Zfp598	0.0679237	0.0125087	7.47E+58	3.53798	88896.8	67003.7	1
Rragc	0.0480869	0.00919359	6.60E+57	4.13628	88467.7	57035.3	1
Eftud2	0.0278309	0.0155818	1.96E+56	5.19188	130879	67222.2	1
Pttg1ip	0.00747367	0.00105181	1.38E+56	8.39177	78774.6	25032.3	1
Ythdf1	0.00679423	0.000936769	2.75E+54	8.66667	73728.4	22685.6	1
Atp6v1g1	0.114892	0.0347458	9.71E+53	2.71658	91462.5	89782.1	1
Dgcr2	0.102334	0.021622	4.51E+52	2.8889	73672	68004.8	1
Rbpj	0.137651	0.0446208	4.34E+52	2.45653	89586.8	97250.4	1
Tinf2	0.0614711	0.0103599	1.68E+50	3.70584	62734.1	45142.5	1
Dcbld1	0.0675727	0.0115447	8.94E+49	3.5466	62061.6	46663.7	1
Memo1	0.0479967	0.0102134	3.61E+49	4.13967	67838.8	43700	1
2310057M21Rik	0.15207	0.0307546	1.70E+48	2.31768	58601.6	67425.4	1
Sdhd	0.190752	0.0444725	1.61E+48	2.01302	61461	81417.9	1
Traf4	0.014954	0.00233482	7.90E+47	6.57515	58450.4	23705.6	1
Ncapd2	0.0924754	0.0176125	7.21E+47	3.04355	58488.6	51246	1
Grpel1	0.123514	0.0290171	4.44E+46	2.61116	60028.4	61304.3	1
Degs2	0.0708257	0.0196954	4.36E+46	3.46881	66534.5	51148.8	1
Zrsr2	0.0856475	0.0183471	3.69E+46	3.16312	58599.4	49402.3	1
Dab2ip	0.210707	0.0471175	1.29E+46	1.88393	54300.8	76861.7	1
Ankrd40	0.128956	0.0263872	9.99E+45	2.54917	54442.6	56951.9	1
Lef1	0.145449	0.0336658	1.76E+45	2.37934	55379.4	62066.9	1
Cbfa2t2	0.0533812	0.00880075	1.11E+45	3.95008	50398.7	34023.8	1
E2f8	0.0866203	0.0172989	1.32E+44	3.14537	50972.4	43214.7	1
Cdc42	0.0412194	0.0128096	1.06E+44	4.4197	62713.3	37838.7	1
Clic4	0.10473	0.0217199	1.04E+44	2.85407	50966.4	47619.9	1
C1qbp	0.166661	0.0477953	1.56E+43	2.19269	55892.6	67974.6	1
Nploc4	0.174768	0.0411705	1.49E+43	2.12887	50032.3	62671.4	1
Rabgef1	0.124108	0.0233731	1.12E+42	2.60423	43901.9	44954.3	1
Snai1	0.0830325	0.0180281	1.15E+41	3.21207	45906.2	38111.4	1
Ube2q1	0.0905458	0.0241744	6.63E+39	3.07621	47244.4	40954.7	1
Uchl4	0.177045	0.0367138	5.88E+39	2.1116	39676.3	50105.8	1
Wbp1l	0.0555678	0.0124404	2.57E+39	3.87975	43582.4	29955.5	1
Rab34	0.010594	0.00812108	1.04E+39	7.43905	69005.6	24736.3	1
Gatad1	0.0956348	0.017189	4.25E+38	2.99185	37058.4	33030.5	1
Pbdc1	0.114358	0.0226401	3.09E+38	2.72342	37905.7	37115.8	1
Eif1a	0.107015	0.0197653	3.71E+37	2.82176	35174.7	33241.4	1
Fam103a1	0.0114063	0.00190788	2.71E+37	7.24766	36758.1	13524.6	1
Tmem41a	0.0631324	0.0106386	2.07E+37	3.66061	34578.4	25189.6	1
Lmnb2	0.0109929	0.00225256	1.03E+37	7.34285	38502.1	13982.6	1
Pde12	0.0530173	0.00872491	6.13E+36	3.96213	33602.7	22615.9	1
Nt5c2	0.0983931	0.0200656	3.80E+36	2.94842	35080.3	31728	1

Usp24	0.208957	0.0471072	2.36E+36	1.89465	33976.8	47821.3	1
Bet1	0.166827	0.0353939	1.26E+36	2.19134	33299	40521.9	1
Eif3m	0.131063	0.0326898	9.87E+35	2.52603	36663.6	38704.7	1
Usp14	0.139556	0.0295523	4.46E+35	2.43718	33015.2	36123.9	1
Adnp2	0.0965852	0.0180473	3.33E+35	2.97671	31856.6	28538.5	1
Rer1	0.0508045	0.00936671	2.97E+35	4.03769	32745.5	21626.6	1
Vti1b	0.0752834	0.0176939	2.20E+35	3.36911	35381.6	28004.7	1
Lhx2	0.0551809	0.00912839	8.59E+34	3.89194	30301.4	20761.8	1
Mier3	0.179525	0.036894	5.93E+34	2.09309	30143.3	38403.6	1
Abcc5	0.106946	0.0301713	4.33E+34	2.82273	36463.8	34447.8	1
Wasf1	0.128637	0.0277112	3.92E+34	2.55272	31564.4	32973.3	1
Mogs	0.140138	0.0306306	3.19E+34	2.43135	31399.3	34438.3	1
Kin	0.17654	0.0436126	2.56E+34	2.1154	32560.8	41046	1
Socs2	0.052947	0.00871315	7.10E+33	3.96447	28453.4	19139	1
Etv2	0.0527289	0.00897698	6.07E+33	3.97176	28727.3	19287.7	1
Evx1	0.134119	0.0269426	3.46E+33	2.49328	28590.2	30578.4	1
Noc4l	0.137317	0.0421444	2.81E+33	2.45995	35136	38088.6	1
Cbx2	0.132152	0.0255858	2.53E+33	2.51426	27903.3	29594.8	1
Wdr89	0.0657524	0.0115007	1.27E+33	3.59218	27566	20463.7	1
Gsr	0.0548129	0.00924914	1.18E+33	3.90364	27383.2	18706.1	1
Pcgf3	0.0875188	0.0159303	9.95E+32	3.12921	27364	23319.2	1
Klf13	0.0965897	0.0257522	9.13E+32	2.97664	32264.5	28904.6	1
Scaf4	0.0602289	0.0103551	1.75E+32	3.74067	26094.5	18602.4	1
Ralgps2	0.193068	0.0457408	1.12E+32	1.99722	27368	36541.6	1
Anxa7	0.107145	0.0198049	8.42E+31	2.81994	25406.4	24025.5	1
Wdr61	0.0906106	0.0169823	6.97E+31	3.07509	25747.3	22327.6	1
Psmd7	0.0285704	0.0145468	6.40E+31	5.13799	40148	20837.2	1
Med23	0.161466	0.0372666	2.06E+31	2.2356	26241.9	31301.9	1
Chaf1b	0.129798	0.0259865	1.46E+31	2.53987	24718.7	25952.7	1
Chaf1a	0.00332378	0.000482468	7.36E+30	10.9499	24359.8	5932.45	1
Fbf1	0.12881	0.0270173	5.77E+30	2.5508	24638.4	25757.6	1
Tspan31	0.0761615	0.0132075	3.02E+30	3.35033	23081.5	18371.5	1
Pald1	0.0557546	0.00923599	2.11E+30	3.8739	22831.4	15716.4	1
Trappc6b	0.0621648	0.0104605	7.74E+29	3.68677	22188.7	16049.2	1
Mrpl15	0.0908855	0.0232983	6.15E+29	3.07039	25938.5	22527.8	1
Vkorc1	0.21681	0.0480018	1.08E+29	1.84738	21326.8	30784.8	1
Cdca7l	0.168341	0.0339919	4.20E+28	2.17916	20396.7	24959.7	1
Tango6	0.167296	0.0338701	2.34E+28	2.18755	20076.8	24474	1
Sqle	0.000624741	7.74E-05	6.40E+27	18.1758	19201.1	2817.09	1
Lysmd2	0.141924	0.0311361	5.53E+27	2.41359	20314.1	22444.1	1
Ptk2	0.206246	0.049704	4.82E+27	1.91147	20451.7	28532	1
Kctd20	0.0850594	0.0150219	1.50E+27	3.17396	18357.5	15423.4	1
Lsm6	0.0514917	0.010143	1.41E+27	4.0138	19684.1	13077.6	1
Ino80e	0.104675	0.0206862	1.33E+27	2.85485	18934	17685.9	1
Ppp2r2a	0.161062	0.0365418	1.06E+27	2.239	19380.8	23082.7	1

Tmem223	0.107006	0.0197558	4.76E+26	2.82189	17733.2	16757.7	1
Cenpe	0.175949	0.0384806	3.42E+26	2.11988	18166.9	22852.7	1
Notch1	0.101484	0.0271639	2.46E+26	2.90149	20680.2	19006.6	1
Fam149a	0.217104	0.0475265	1.84E+26	1.84566	17343.1	25057.8	1
Zpr1	0.0822105	0.0287668	1.71E+26	3.22785	23367.2	19304.6	1
Plekha3	0.155909	0.0346754	1.16E+26	2.28341	17903.2	20908.1	1
1300002E11Rik	0.0629511	0.0106017	9.11E+25	3.66547	16732.5	12173.1	1
Triap1	0.168198	0.0492717	5.05E+25	2.1803	20013.7	24478.3	1
Ddx28	0.158477	0.0314	4.46E+25	2.26106	16335.4	19265.7	1
Nif3l1	0.147436	0.049647	1.17E+25	2.36048	20695.1	23379.6	1
Ube2g1	0.0615679	0.0103719	8.54E+24	3.70317	15454.6	11128.9	1
Sil1	0.0883666	0.0156649	3.97E+24	3.11414	15024.3	12865.5	1
Ppil3	0.128411	0.0243461	2.19E+24	2.55524	14709.9	15351.4	1
Tsen15	0.0749477	0.0464054	1.35E+24	3.37636	26430.9	20875.2	1
Etfa	0.048544	0.038434	9.96E+23	4.11922	28857	18681.2	1
Hexim1	0.160751	0.0319533	6.59E+23	2.24164	14087	16758	1
Ddx18	0.144595	0.0328841	4.96E+23	2.38755	15062.9	16823.8	1
Bahcc1	0.107674	0.0197808	2.25E+23	2.81259	13549.7	12846.7	1
Herpud2	0.124556	0.0238857	2.19E+23	2.59904	13636.9	13991.7	1
B2m	0.00822463	0.00244643	9.43E+22	8.12207	16878.2	5541.52	1
Cog4	0.0286467	0.0044082	4.30E+22	5.13253	12734.8	6616.54	1
Smox	0.065883	0.011174	3.59E+22	3.58886	12635.2	9388.45	1
Chmp2a	0.0146185	0.00211834	3.03E+22	6.62978	12562.8	5053.08	1
Rfc5	0.106977	0.0274355	2.43E+22	2.82229	14544	13742.1	1
Tbccd1	0.131793	0.0280524	2.15E+22	2.51813	13053.8	13823.8	1
Dlg3	0.169025	0.0346838	3.53E+21	2.17369	11647.3	14288.8	1
Pygo2	0.118265	0.0376582	2.55E+21	2.67422	14749.5	14707.8	1
Tax1bp3	0.0389171	0.00813355	2.38E+21	4.52814	12615.5	7429.41	1
Ube2f	0.165406	0.0371229	1.91E+21	2.2029	11920.7	14430.3	1
2310033P09Rik	0.0620081	0.0104223	1.15E+21	3.69106	11014.2	7957.39	1
Metap1	0.00133591	0.00020242	2.44E+20	14.516	10868.2	1996.54	1
Rnf2	0.196709	0.0414893	1.57E+20	1.97283	10188.9	13772.3	1
BC029722	0.1177	0.023652	8.00E+19	2.6812	10182.6	10127.4	1
Atg5	0.0360822	0.00647948	3.65E+19	4.67318	9989.96	5700.6	1
Slc30a6	0.0244516	0.00752402	2.40E+19	5.46303	12137	5924.43	1
Cirh1a	0.11347	0.0237524	1.23E+19	2.73489	9572.06	9333.27	1
Hmg20a	0.187687	0.0387142	1.21E+19	2.0343	9043.55	11854.8	1
Mtf1	0.0759289	0.0353603	1.88E+18	3.35528	13057.7	10377.8	1
Ptpn23	0.037163	0.0152607	1.07E+18	4.61626	11831.2	6834.51	1
Mex3d	0.0201097	0.00298844	1.01E+18	5.88917	8053.62	3646.75	1
Map3k3	0.0557305	0.0111995	1.41E+17	3.87466	7893.14	5432.32	1
BC030867	0.123232	0.0239801	8.21E+16	2.61447	7217.08	7361.18	1
Smap1	0.142884	0.0420339	3.78E+16	2.40416	8422.96	9342.66	1
Ung	0.10643	0.0349541	1.42E+16	2.82994	8554.6	8061.03	1
Tssc1	0.112184	0.0218674	1.40E+16	2.75171	6632.87	6427.88	1

Stard7	0.206479	0.0444051	1.25E+16	1.91001	6496.87	9070.62	1
Scap	0.115503	0.0216963	1.12E+16	2.70879	6428.45	6328.48	1
Ahsa2	0.181606	0.0389966	1.03E+16	2.07779	6529.54	8380.1	1
Foxk2	0.138212	0.0444841	7.96E+15	2.4508	8142.27	8859.44	1
Gtpbp6	0.000111698	1.35E-05	5.51E+15	29.5448	6182.27	558.002	1
Gm8994	0.0292503	0.0126796	3.44E+15	5.08995	8957.45	4692.88	1
Cdc42se2	0.161667	0.0348861	2.81E+15	2.2339	6170.96	7366.43	1
Itgb1bp1	0.0331028	0.00974281	2.55E+15	4.84171	7524.28	4144.14	1
Zfp628	0.0862976	0.0152435	1.17E+15	3.15123	5638.02	4771.06	1
Rbck1	0.0511323	0.00888664	1.06E+15	4.02624	5737.28	3799.93	1
Ankib1	0.119341	0.0224646	9.63E+14	2.66101	5592.62	5604.51	1
Rfx1	0.106741	0.0214263	7.34E+14	2.82559	5719.98	5398.26	1
Bod1	0.10892	0.0214396	5.28E+14	2.79546	5552.25	5296.44	1
Coa5	0.110783	0.0281805	2.93E+14	2.77029	6039.62	5813.71	1
Auh	0.0166194	0.00265431	1.23E+14	6.32487	5083.21	2143.16	1
Crtc3	0.204122	0.042977	4.82E+13	1.92483	4648.79	6440.46	1
Rrp12	0.223475	0.0484079	3.04E+13	1.80887	4524.33	6669.84	1
Wdr90	0.0226236	0.014942	1.08E+13	5.62996	7471.86	3539.09	1
Spats2	0.154742	0.0370405	6.98E+12	2.29372	4515.73	5249.96	1
Cpsf2	0.0482816	0.00791667	2.37E+12	4.12899	3814.37	2463.47	1
Avpi1	0.104684	0.0198564	2.37E+12	2.85472	3868.5	3613.66	1
Elp4	0.133018	0.0268086	1.04E+12	2.50497	3677.82	3915.23	1
Stk25	0.0565687	0.00940692	1.03E+12	3.84869	3585.65	2484.41	1
Mkks	0.0959956	0.0174886	4.64E+11	2.98608	3399.35	3035.73	1
Dnaaf2	0.0324307	0.00504664	3.40E+11	4.88235	3303.7	1804.43	1
L3hypdh	0.043261	0.00695198	2.83E+11	4.32965	3258.31	2006.82	1
Coq2	0.106426	0.0195754	1.30E+11	2.83001	3074.6	2897.14	1
Tmem229a	0.209901	0.0445147	1.03E+11	1.88885	3011.15	4251.11	1
Setd1b	0.225968	0.0491198	9.82E+10	1.79482	3007.95	4469.1	1
Wwc2	0.0860815	0.0154578	5.95E+10	3.15517	2903.64	2454.08	1
Gm6644	0.0173396	0.00263059	3.59E+10	6.22613	2799.82	1199.17	1
Dna2	0.213146	0.0499498	3.10E+10	1.86917	2880.34	4109.26	1
Fam101b	0.193974	0.0471041	1.89E+10	1.9911	2850.35	3817.46	1
Kdm4b	0.0523348	0.0103451	1.79E+10	3.98501	2809.37	1879.96	1
Atmin	0.173837	0.0369106	1.28E+10	2.13601	2598.99	3244.66	1
Tmem50b	0.16899	0.0339886	1.00E+10	2.17397	2483.89	3046.83	1
Gal	0.0572818	0.00952313	3.31E+09	3.82698	2250.05	1567.85	1
Snx15	0.102969	0.0195177	3.09E+09	2.87957	2277.4	2109.02	1
Nfu1	0.212034	0.0468974	2.13E+09	1.87588	2207.46	3138.02	1
Sult4a1	0.0833204	0.0158157	1.31E+09	3.20658	2133.99	1774.67	1
Cd9	0.0795212	0.015525	1.18E+09	3.28088	2144.68	1743.18	1
Eif2d	0.167624	0.0479716	4.89E+08	2.18491	2258.34	2756.28	1
Cachd1	0.0437193	0.0106248	4.63E+08	4.31015	2194.29	1357.59	1
Nomo1	0.0697902	0.0120517	2.04E+08	3.49308	1722.08	1314.66	1
Glrx2	0.102996	0.0213116	1.28E+08	2.87917	1728.53	1600.95	1

Iqsec1	0.14405	0.0333705	1.09E+08	2.39281	1749.94	1950.22	1
Poc1a	0.0649579	0.0113646	8.30E+07	3.61257	1578.55	1165.23	1
9430038I01Rik	0.158891	0.0419811	6.46E+07	2.2575	1754.54	2072.55	1
Slc39a4	0.117056	0.0217996	5.54E+07	2.68923	1488.94	1476.45	1
Phgdh	0.051911	0.0105321	4.40E+07	3.99941	1578.59	1052.55	1
Pin1rt1	0.073876	0.0128904	3.97E+07	3.39978	1441.08	1130.33	1
Strbp	0.18551	0.0459658	3.92E+07	2.04967	1580.88	2056.76	1
St6galnac4	0.209282	0.0444538	1.98E+07	1.89265	1323.37	1864.58	1
Cmip	0.0828822	0.0217696	1.77E+07	3.21494	1558.25	1292.51	1
Git1	0.096809	0.0175918	1.53E+07	2.97318	1287.49	1154.76	1
Gm5434	0.193987	0.0428002	1.11E+07	1.99101	1271.94	1703.58	1
Prr22	0.0809306	0.0141461	8.41E+06	3.25282	1190.62	976.077	1
Gm15645	0.00645119	0.0363888	5.94E+06	8.81885	4785.02	1446.91	1
Cep120	0.0918034	0.0180984	1.91E+06	3.05483	1023.11	893.107	1
Mpv17l2	0.0800876	0.0141013	655952	3.26953	843.306	687.81	1
Mtx2	0.0483862	0.00885535	490362	4.12508	842.007	544.316	1
Plcxd1	2.63E-05	3.07E-06	473069	43.7162	800.497	48.8299	1
Prpf39	0.0907228	0.0241202	336165	3.07317	908.116	787.994	1
Tank	0.165193	0.0330392	56671.3	2.20464	561.02	678.592	1
Celf6	0.22043	0.0474224	42000.4	1.82629	531.071	775.444	1
Smarcad1	0.0526023	0.00873243	41605.8	3.976	531.81	356.68	1
Vps36	0.119425	0.0227713	7910.67	2.65998	380.76	381.716	1
Rab18	0.0198597	0.00890494	5618.22	5.91714	525.555	236.851	1
Gm7977	0.120633	0.0332838	4939.97	2.64535	407.993	411.281	1
Akr1b3	0.00725532	0.00100726	4412.96	8.4766	330.79	104.064	1
Hiat1	0.217567	0.048708	2774.91	1.84294	301.66	436.491	1
4933417G07Rik	0.206079	0.0481736	2494.96	1.91251	302.861	422.287	1
Thap1	0.125988	0.0274301	1908.28	2.58257	285.789	295.095	1
Sh2b3	0.0528126	0.00868746	1391.75	3.96896	245.37	164.86	1
Proser1	0.116256	0.0255337	699.927	2.69926	217.115	214.493	1
Atoh8	0.164472	0.0328948	623.416	2.21057	194.037	234.073	1
Gm10767	0.07929	0.0451265	583.486	3.28554	332.879	270.176	1
Gm6498	0.14658	0.0359189	504.439	2.36857	203.273	228.856	1
Irf1	0.0786328	0.0136833	445.81	3.29889	174.253	140.858	1
2900097C17Rik	0.189907	0.0494107	209.331	2.01885	151.321	199.878	1
Ankzf1	0.0584574	0.00982517	179.646	3.7919	126.606	89.0358	1
Slc22a18	0.185327	0.038167	157.333	2.05097	119.966	155.979	1
Hnrnpul2	0.078508	0.0136752	110.614	3.30144	103.771	83.819	1
Peli1	0.106847	0.0484217	86.7674	2.8241	146.018	137.878	1
Ecm1	0.0828761	0.0147253	67.8734	3.21506	83.7544	69.4685	1
Dtl	0.0625782	0.0106595	42.994	3.67553	66.5732	48.3001	1
Otud6b	0.0188789	0.00779012	36.5452	6.03126	88.3137	39.0471	1
Lin7c	0.0818741	0.0253887	34.177	3.23436	75.4023	62.1676	1
Strn3	0.137463	0.032075	33.2432	2.45845	63.1758	68.5263	1
Acy1	0.026277	0.00400333	31.2795	5.31116	55.55	27.891	1

Per1	0.216187	0.0498494	25.962	1.85106	51.7802	74.5954	1
Gm6607	0.0111353	0.0317714	24.9596	7.30956	157.62	57.5029	1
Pxylp1	0.20876	0.0442095	22.4156	1.89586	45.2886	63.7016	1
Iqce	0.0470538	0.00773985	13.2999	4.17558	31.5383	20.1414	1
Kpna1	0.0629406	0.0115573	11.652	3.66575	29.2493	21.2775	1
Arhgef4	0.0990644	0.0179019	11.4038	2.93807	27.7431	25.1803	1
Glce	0.106625	0.0205077	10.7812	2.82721	27.0734	25.536	1
Donson	0.106306	0.0209762	10.0519	2.83169	25.8051	24.3012	1
Pcsk5	0.0619699	0.0105377	10.0263	3.6921	25.0053	18.0604	1
Spast	0.0760247	0.0181802	8.99205	3.35324	25.9593	20.6442	1
Vash2	0.134293	0.0344473	8.73074	2.49143	25.3758	27.1606	1
Csnk1g1	0.0130286	0.00268395	7.65344	6.91181	21.9459	8.46703	1
Trib2	0.0642673	0.0109993	6.98711	3.63054	17.7947	13.0703	1
Msh6	0.0687241	0.014245	6.90718	3.51854	18.9751	14.381	1
Skil	0.00146504	0.000201549	5.53289	14.1171	14.0182	2.64797	1
Lrrc40	0.0547787	0.0196416	5.21757	3.90473	17.7017	12.0891	1
Lrch3	0.063632	0.0107325	4.88975	3.64729	11.7968	8.62504	1
Ncapg2	0.00504723	0.00160441	4.66633	9.5669	14.6289	4.07764	1
Rnf219	0.127803	0.0274309	4.54887	2.56204	11.4023	11.8679	1
Ube2v2	0.0791429	0.0233127	4.52754	3.28852	13.4706	10.9234	1
Pabpc2	0.0973747	0.0260243	3.99954	2.96428	10.7721	9.69063	1
BC005764	0.0650683	0.0142532	3.82041	3.60972	9.36647	6.91945	1
Tctex1d2	0.141539	0.0275049	3.66094	2.4174	7.90533	8.72049	1
Setdb1	0.0326069	0.0324175	3.65813	4.8716	17.2429	9.4386	1
Mpp1	0.0646052	0.0148239	3.6322	3.62172	8.84914	6.51561	1
Insig2	0.106427	0.0222487	3.59562	2.82999	8.14661	7.67645	1
Fbxo46	0.170963	0.0450952	3.47079	2.15836	8.34454	10.3097	1
Fut8	0.0787473	0.016945	3.26935	3.29655	7.19768	5.82239	1
Zfp850	0.170123	0.0466171	3.12238	2.16498	7.13484	8.78819	1
Med14	0.00311027	0.000410148	3.11911	11.1832	6.0661	1.44648	1
Kansl1l	0.0466517	0.00756971	2.93628	4.19117	5.43777	3.45982	1
Tbc1d23	0.0661281	0.0116061	2.79087	3.58264	5.00299	3.72387	1
Ptprk	0.0958444	0.0173434	2.77226	2.9885	4.88404	4.35808	1
Zfp719	0.0908102	0.0170903	2.57441	3.07168	4.28969	3.72408	1
Appl1	0.123346	0.0415468	2.46008	2.61313	5.06037	5.16405	1
Ndc1	0.00586251	0.0008202	2.45539	9.10509	3.81409	1.11706	1
Casp2	0.208359	0.0458428	2.4181	1.89834	3.72831	5.23731	1
Zyg11b	0.0474406	0.0104963	2.36886	4.16074	3.93769	2.52371	1
Ryr1	0.189912	0.0392586	2.35231	2.01881	3.42665	4.52629	1
Zkscan1	0.174939	0.0358334	2.30784	2.12756	3.29324	4.12772	1
Wdhd1	0.0839999	0.042258	2.29269	3.19374	5.30184	4.42686	1
Pter	0.00840685	0.00151476	2.27163	8.06124	3.43176	1.13523	1
Adal	0.128197	0.0265006	2.24878	2.55763	3.20415	3.34075	1
Ermard	0.212112	0.0452881	2.11287	1.87541	2.62619	3.73421	1
Gm21319	0.0772811	0.013641	2.11265	3.32677	2.63853	2.11499	1

Hist1h4m	0.176837	0.0373625	2.08994	2.11316	2.59615	3.27616	1
Sap130	0.0988383	0.0191398	2.07367	2.94155	2.56902	2.32895	1
Dcaf5	0.170775	0.0358165	2.06042	2.15984	2.49741	3.08345	1
Slc35f5	0.187541	0.0447874	2.0509	2.03532	2.61208	3.42233	1
Casd1	0.0179322	0.049271	-3.24783	6.14865	22.3592	9.69718	1
Phf21a	0.000777518	0.0246237	-3.29271	17.0504	44.6159	6.9779	1
Has2	0.0280163	0.0207424	-3.68004	5.1782	14.9654	7.70685	1
Dcbld2	0.00441472	0.0336759	-4.86729	9.99464	53.6828	14.3231	1
Smtn	0.00128999	0.0399138	-55.2404	14.6697	552.417	100.419	1
Ptges3	0.00347274	0.0331588	-892849	10.7979	4314.29	1065.47	1
Cnih1	6.21E-05	0.0158469	-2.34E+25	34.6865	178882	13752.3	1
Ccnd2	0.00420332	0.0173501	-3.23E+30	10.155	78787.5	20689.3	1
Sod1	0.00111641	0.00547115	-8.05E+46	15.3189	176705	30760.2	1
Csrp2	0.0245826	0.0209845	-4.40E+66	5.45167	219701	107466	1
