# Supplementary material for: Single-Cell Expression Profiling Reveals a Dynamic State of Cardiac Precursor Cells in the Early Mouse Embryo
Source: PLoS One. 2015 Oct 15;10(10):e0140831. doi: 10.1371/journal.pone.0140831 (PMC4607431; doi:10.1371/journal.pone.0140831)
Supplement: S6 Table — (PDF) [file pone.0140831.s016.pdf]

Table S6. The enriched genes in EB *Tbx5*$^{+}$ CPs filtered via ANOVA

ID	p-value (Attribute)	p-value	Fold-Change	F (Attribute)	SS (Attribute)	SS (Error)	F (Error)
Hand1	0.00431322	0.000706759	8.99E+307	10.0704	4.14E+06	1.09E+06	1
Pcbp1	0.0312566	0.0289292	8.99E+307	4.95592	1.27E+07	6.83E+06	1
Eef1a1	0.0651432	0.0141259	8.99E+307	3.60779	1.44E+07	1.06E+07	1
H3f3a	0.0593293	0.0281064	1.90E+260	3.76645	2.65E+06	1.88E+06	1
Nudcd2	0.0599293	0.0399858	9.38E+167	3.7492	1.31E+06	934275	1
Egln1	0.133652	0.0256286	6.61E+162	2.49822	659280	703734	1
Hsp90aa1	0.0681244	0.0135892	1.50E+162	3.53308	697382	526364	1
Ppp1ca	0.0895064	0.0251551	7.74E+158	3.09415	770779	664288	1
Bmp4	0.120177	0.0239522	1.39E+155	2.65086	615285	618954	1
Ide	0.0849393	0.0164044	3.99E+150	3.17619	585940	491944	1
Odc1	0.00537638	0.0162733	2.00E+148	9.36997	1.67E+06	475382	1
Syncrip	0.0504573	0.0173791	1.19E+141	4.04992	672327	442693	1
Aldoa	0.0709749	0.0247939	1.82E+135	3.46535	621339	478135	1
Rbm7	0.224646	0.0488797	2.84E+119	1.80225	355731	526352	1
Capzb	0.0585845	0.0229362	4.93E+112	3.78816	454684	320074	1
Arpc1a	0.0346006	0.0102641	7.39E+106	4.75473	363061	203621	1
Laptm4b	0.0508269	0.0377109	1.93E+96	4.03691	450804	297788	1
Psmd2	0.0748882	0.0131319	9.11E+94	3.37765	225315	177887	1
E430025E21Rik	0.00129985	0.000177121	1.23E+91	14.6361	210382	38331.1	1
Daxx	0.212082	0.0484599	2.32E+85	1.87559	188172	267539	1
Cnih1	6.21E-05	7.77E-06	1.59E+84	34.6865	178882	13752.3	1
Pdcd6ip	0.0638507	0.0133276	3.17E+83	3.6415	188997	138403	1
Cbx1	0.0463845	0.00788034	1.03E+81	4.20163	166055	105391	1
Dnajc8	0.016931	0.005565	6.28E+80	6.28151	216359	91850.2	1
Rps4x	0.140794	0.0299836	5.30E+78	2.42479	161321	177413	1
Mthfd1	0.146878	0.0327554	8.08E+73	2.36575	144914	163347	1
Pycrl	0.21982	0.0484463	1.64E+73	1.82981	135012	196759	1
Psmd12	0.0499451	0.00815729	5.52E+72	4.06815	131385	86123	1
Uqcr10	0.126774	0.0284089	2.35E+69	2.57364	129509	134190	1
Eif4g2	0.0164317	0.00447079	1.22E+69	6.35148	147575	61959.1	1
A430005L14Rik	0.127974	0.0244535	3.72E+65	2.56013	107181	111641	1
Ppme1	0.193549	0.0461978	2.96E+65	1.99396	114630	153303	1
D8Ertd82e	0.0938521	0.0168536	9.68E+64	3.02076	104993	92685.6	1
Cyb5r3	0.153921	0.0332947	1.61E+63	2.30103	103939	120455	1
Wdr73	0.108533	0.0199399	6.05E+57	2.80076	82912.8	78943.3	1
Taf2	0.0927041	0.0207801	2.36E+56	3.03973	87264.8	76554.8	1
Khdrbs3	0.103714	0.0189589	9.39E+55	2.86871	77909.6	72422.3	1
Itpa	0.199685	0.0491418	1.31E+55	1.9533	82324.5	112390	1
Pagr1a	0.0833914	0.0163985	2.09E+53	3.20524	74110.6	61657.9	1
Ccnd2	0.00420332	0.000874976	3.58E+52	10.155	78787.5	20689.3	1
Chic2	0.11932	0.0230708	6.77E+51	2.66126	67751.6	67889.1	1
Msx1	0.184317	0.0418035	7.53E+50	2.05819	67720.3	87741.1	1

Dera	0.189822	0.0441777	4.61E+48	2.01943	62607.8	82673.7	1
Wdr70	0.0821536	0.0152768	3.36E+48	3.22895	59974	49530.3	1
Armc1	0.16902	0.0339846	4.23E+47	2.17373	56322.7	69095	1
Rwdd1	0.195149	0.045901	3.04E+46	1.98321	57269.6	77006.1	1
Med19	0.130882	0.030673	2.88E+46	2.528	59232.1	62481	1
Nkd1	0.0209324	0.00362435	8.47E+45	5.80009	55238	25396.4	1
Syf2	0.0113786	0.00187981	1.23E+45	7.25389	53116.1	19526.5	1
Phtf1	0.10933	0.0467767	7.06E+44	2.78988	75774.1	72427.7	1
Cox18	0.162021	0.0337259	6.14E+44	2.23094	50925.6	60872	1
Rpl7	0.0758893	0.0205735	4.71E+42	3.35612	54959.1	43668.7	1
Bex2	0.0206182	0.00758623	5.51E+41	5.83359	60310.6	27569.4	1
Vrtn	0.105396	0.0298577	3.62E+41	2.84456	52630.5	49339.1	1
Oxa1l	0.155124	0.0422178	5.16E+40	2.29034	48509.2	56479.8	1
Slc16a3	0.127305	0.0252244	3.46E+40	2.56764	41692	43299.9	1
Cited2	0.0363344	0.0181888	2.58E+38	4.65971	58512.5	33485.7	1
Paics	0.0114899	0.00174134	2.06E+37	7.22893	35337.9	13035.7	1
Sypl	0.192604	0.0399918	3.00E+35	2.00037	31264.7	41678.6	1
Cyc1	0.0545032	0.0104805	2.41E+35	3.91356	33032.3	22508	1
Mrpl30	0.0682996	0.0243973	5.73E+34	3.52882	41467.5	31336.3	1
Nudt1	0.175232	0.0370819	3.48E+34	2.12533	30279.2	37991.6	1
Prdx1	0.116945	0.0359324	2.96E+34	2.69061	37568.6	37234.3	1
Mrrf	0.106328	0.0198889	1.66E+34	2.83138	29362.4	27654.2	1
Ap1ar	0.0880319	0.0157209	1.11E+34	3.12006	28881.3	24684.4	1
Upp1	0.157799	0.0312373	1.10E+34	2.26692	28775.4	33849.6	1
4930427A07Rik	0.200832	0.0438627	5.48E+33	1.94586	28887.2	39587.9	1
Vma21	0.0674776	0.0120465	1.19E+33	3.54894	27712.3	20822.9	1
Dnaja2	0.142662	0.0377855	5.93E+32	2.40633	31162.6	34533.9	1
Scaf11	0.123597	0.0352309	5.81E+32	2.61019	32595.7	33300.9	1
Zfp710	0.107247	0.0208349	5.57E+32	2.81853	27334	25861.2	1
Usf2	0.0747976	0.0469628	2.86E+31	3.37962	45252.3	35706	1
Grwd1	0.119524	0.0393012	7.56E+30	2.65878	31196.4	31288.9	1
Syngr2	0.00183005	0.000236122	3.99E+30	13.1922	23281.6	4706.14	1
Stxbp1	0.0745143	0.0132747	3.75E+30	3.38578	23519.8	18524.4	1
Arrb1	0.0297364	0.0146927	2.95E+30	5.05645	36414.8	19204.4	1
Elp3	0.0955357	0.0182081	9.39E+29	2.99344	22896.7	20397.2	1
Arhgap21	0.120074	0.0265562	7.96E+29	2.6521	24005.8	24137.7	1
Gmeb1	0.131866	0.0389034	6.95E+29	2.51734	27441.1	29068.9	1
Tpd52l2	0.0577592	0.00994631	3.18E+29	3.81263	21908.7	15323.6	1
Mapkapk5	0.0444417	0.00727969	2.53E+29	4.27994	21609.6	13464.1	1
Cmpk1	0.171424	0.0347121	1.02E+29	2.15475	20933	25906.2	1
Slc7a6os	0.0734843	0.0126796	2.32E+28	3.40845	19992	15641.2	1
Fam58b	0.0885871	0.0167313	1.30E+28	3.11025	20167.3	17291.1	1
Ndufa10	0.173792	0.0437406	9.68E+27	2.13636	21788.7	27197.2	1
Plxna1	0.199622	0.0442627	8.15E+27	1.95371	19946.2	27225	1
Rnd2	0.0793133	0.0138198	5.71E+27	3.28507	19128.9	15527.9	1

Ing2	0.0992473	0.0179594	4.70E+27	2.93527	19021.4	17280.8	1
Exosc6	0.0476933	0.0120784	4.25E+27	4.15113	22639.9	14543.8	1
Ppap2c	0.127683	0.0261029	2.70E+27	2.56339	19368.1	20148.4	1
Raver2	0.081378	0.0145812	1.29E+27	3.24403	18435.4	15154.3	1
Pex11b	0.187775	0.0387284	1.93E+26	2.03368	17159.4	22500.3	1
Zfp362	0.222172	0.0480917	6.94E+25	1.81629	16628.4	24413.8	1
9430016H08Rik	0.215654	0.0492034	2.16E+25	1.85421	16523.6	23763.7	1
Nt5c3	0.068204	0.0118685	9.12E+24	3.53114	15604.9	11784.6	1
Rps6ka6	0.0263392	0.00568034	7.82E+23	5.30623	16105.2	8093.73	1
Lrrc16a	0.161206	0.034879	5.34E+23	2.23779	14581.8	17376.4	1
Hmgn2	0.0301027	0.0258714	2.10E+23	5.03165	27364.2	14502.4	1
Tdp1	0.00187884	0.000273783	3.55E+22	13.0861	13101.7	2669.83	1
Cnot11	0.174574	0.035461	6.43E+21	2.13036	11825.7	14802.8	1
Tab1	0.137661	0.0264672	3.89E+21	2.45643	11573.8	12564.4	1
Fam189b	0.0567357	0.00947835	1.85E+21	3.84358	11257.4	7810.38	1
Dnmbp	0.1226	0.0271708	1.68E+21	2.6219	12088.6	12295	1
Frmd8	0.175693	0.0358542	8.41E+20	2.12182	10904.4	13704.4	1
Atp6v0c	0.0314	0.00933933	3.50E+20	4.94675	13441.4	7245.9	1
C2cd5	0.0946994	0.0191193	3.15E+20	3.00694	10993.9	9749.85	1
Zfp647	0.14108	0.0296485	2.88E+20	2.42195	10823.8	11917.4	1
M6pr	0.197831	0.0417431	1.29E+20	1.96542	10096.3	13698.5	1
Cthrc1	0.057917	0.00964321	4.26E+19	3.80792	9566.67	6699.49	1
Polr3a	0.0916235	0.0174662	2.49E+19	3.05786	9617.4	8387.04	1
Supt5	0.0234613	0.00903367	1.62E+19	5.55145	13013.2	6250.93	1
Slc9a3r1	0.208614	0.0448189	6.69E+18	1.89676	8869.03	12469	1
Tbc1d8	0.0811198	0.0141843	6.32E+18	3.2491	8776.32	7203.09	1
Ivd	0.117669	0.0257666	1.60E+18	2.68159	8867.7	8818.34	1
Ythdf2	0.0465609	0.00753863	1.34E+17	4.19472	7282.36	4629.54	1
Hspb11	0.118359	0.0274569	3.03E+16	2.67306	7469.64	7451.78	1
Trmt61a	0.12253	0.0492613	4.52E+15	2.62273	8930.04	9079.63	1
Eif4ebp2	0.0655326	0.0111072	1.15E+15	3.59779	5633.05	4175.19	1
Hnrnpa0	0.0566285	0.0116557	4.43E+14	3.84686	5810.23	4027.68	1
Matr3	0.15338	0.035163	2.43E+14	2.30587	5546.09	6413.88	1
Gnpnat1	0.151001	0.0329595	1.96E+14	2.32741	5348.04	6127.6	1
Vasp	0.134541	0.0321976	1.25E+14	2.48882	5499.48	5892.46	1
Mocs3	0.0885512	0.0157029	7.61E+13	3.11088	4784.25	4101.09	1
Selt	0.121336	0.0228757	5.82E+13	2.63692	4716.12	4769.32	1
Gtpbp1	0.171014	0.0458368	8.51E+12	2.15797	4817.98	5953.73	1
Ccnb1	0.140679	0.0277345	7.33E+12	2.42593	4150.44	4562.3	1
Abcb6	0.138853	0.0290023	6.95E+12	2.44429	4257.67	4645.03	1
Rfc4	0.143439	0.0332208	5.10E+12	2.39874	4374.35	4862.94	1
Ptges3	0.00347274	0.000615132	4.00E+12	10.7979	4314.29	1065.47	1
Med12	0.0499076	0.00823912	3.22E+12	4.06949	3900.78	2556.12	1
Tfip11	4.02E-05	5.16E-06	2.84E+12	39.003	3944.69	269.702	1
Sub1	0.0292014	0.00642659	2.32E+12	5.09336	4337.61	2270.99	1

Gm15421	0.110224	0.0207683	1.48E+12	2.77779	3715.46	3566.83	1
Xpo1	0.0164061	0.00250182	1.45E+12	6.35514	3728.9	1564.67	1
Nucks1	0.099579	0.0425712	7.85E+11	2.9302	5321.42	4842.83	1
Anks3	0.150887	0.0326241	1.62E+11	2.32846	3274.97	3750.66	1
Ncbp1	0.0760611	0.0135717	7.89E+10	3.35246	2986.04	2375.2	1
Znhit3	0.140021	0.0297918	2.40E+10	2.43251	2804.95	3074.95	1
Mplkip	0.181887	0.042172	1.32E+10	2.07574	2714.75	3487.58	1
Aldoart2	0.0632813	0.0331277	6.94E+09	3.65662	3993.91	2912.64	1
Farsa	0.10008	0.0192087	6.87E+09	2.92258	2465.87	2249.95	1
Zfand6	0.19557	0.0442838	6.44E+09	1.9804	2497.69	3363.21	1
Bzw1	0.0222664	0.00662621	1.41E+09	5.66455	2671.89	1257.83	1
Gm15645	0.00645119	0.00961108	1.34E+09	8.81885	4785.02	1446.91	1
Lrrc61	0.0601845	0.0100742	5.28E+08	3.74193	1889.19	1346.32	1
Dgat2	0.161064	0.0328977	4.57E+08	2.23899	1886.96	2247.39	1
Chordc1	0.0385064	0.00727797	1.59E+08	4.54832	1786.7	1047.54	1
Phf2	0.0679308	0.0115676	9.32E+07	3.53781	1577.06	1188.73	1
Top2a	0.20048	0.0461845	8.42E+07	1.94814	1640.96	2246.19	1
Ppa2	0.160044	0.0325684	3.16E+07	2.24764	1413.73	1677.29	1
Gnai3	0.0678049	0.0138795	2.19E+07	3.54089	1444.88	1088.15	1
Braf	0.00965753	0.00188759	1.14E+07	7.68381	1379.83	478.869	1
Ddx19a	0.16777	0.0339625	9.90E+06	2.18374	1220.32	1490.2	1
Hspa1l	0.156367	0.0442436	6.72E+06	2.27939	1392.22	1628.76	1
Rpf2	0.00286779	0.000375504	5.77E+06	11.4737	1134.98	263.785	1
Ube2d2a	0.120818	0.0257886	4.07E+06	2.64312	1152.8	1163.07	1
Hnrnpa3	0.039231	0.00733942	1.88E+06	4.51289	1040.93	615.083	1
Gas6	0.123733	0.0270413	1.16E+06	2.6086	979.186	1000.98	1
Mir8094	0.0679658	0.0255233	609465	3.53695	1176	886.639	1
Xrn2	0.026107	0.00408542	280684	5.32475	744.793	372.997	1
Nckap1	0.0309856	0.00612401	159593	4.97339	736.256	394.771	1
Eif3e	0.210165	0.0481358	116827	1.88724	664.763	939.309	1
Dhx15	0.212024	0.0471623	102207	1.87594	638.532	907.681	1
Itpk1	0.0540267	0.00892336	95037.6	3.92895	615.545	417.784	1
Metap2	0.0930132	0.0364859	94488.9	3.0346	889.594	781.735	1
Coa7	0.0916257	0.0165401	60116.4	3.05782	570.078	497.153	1
Smc3	0.0280901	0.00712219	56158.1	5.17279	675.747	348.36	1
Slc38a4	0.176303	0.0431077	55015.5	2.1172	614.748	774.293	1
Kpna2	0.0478333	0.0105055	35536.5	4.14583	579.42	372.693	1
Btf3l4	0.118026	0.0261043	28021.3	2.67716	531.757	529.672	1
Mgat2	0.200622	0.0472498	22856	1.94722	502.353	687.958	1
Magoh	0.0440237	0.00715968	12346.4	4.29735	417.473	259.058	1
Trim59	0.0824838	0.014742	12341	3.22258	419.042	346.755	1
Trmt10c	0.158636	0.0331548	10315.6	2.25969	410.669	484.631	1
Mlxip	0.0430238	0.0070108	6051.74	4.33984	357.31	219.554	1
Nrp1	0.0492423	0.00953917	5216.88	4.09354	367.264	239.248	1
Canx	0.103358	0.0479242	4889.58	2.87389	535.234	496.641	1

D10Wsu102e	0.0824136	0.0146198	4327.47	3.22393	329.966	272.931	1
Fem1b	0.143141	0.0315616	3439.81	2.40165	330.69	367.18	1
Slc39a6	0.0909975	0.0162125	3127.04	3.06848	303.339	263.617	1
Plin2	0.077823	0.0135892	1763.94	3.31552	262.251	210.928	1
Cbx3	0.0159775	0.00443186	1691.09	6.41748	324.54	134.857	1
Cul4b	0.023816	0.00442209	1577.96	5.51926	273.721	132.25	1
Txndc9	0.108563	0.0202239	1146.17	2.80034	233.867	222.703	1
Sf3b6	0.091542	0.0201858	853.262	3.05924	234.33	204.26	1
Rap2c	0.102611	0.0210319	802.644	2.88482	221.055	204.339	1
Ugp2	0.0766439	0.0133502	798.007	3.34013	209.537	167.288	1
Ireb2	0.13487	0.0469345	651.652	2.48536	266.24	285.662	1
Tab2	0.031954	0.00534938	568.269	4.91182	193.769	105.199	1
Arv1	0.124553	0.0311213	373.537	2.59908	188.025	192.914	1
Greb1l	0.0473493	0.0208624	298.084	4.16423	230.723	147.749	1
Siah1b	0.185607	0.0384082	287.553	2.04898	150.619	196.024	1
Psmc6	0.0673385	0.0143699	268.013	3.55238	160.942	120.814	1
Sccpdh	0.131191	0.0250616	242.883	2.52464	141.502	149.462	1
Polr2b	0.0248348	0.0047397	179.626	5.43	137.233	67.3948	1
Utp3	0.11465	0.0217237	167.532	2.71968	124.013	121.596	1
Atf2	0.153704	0.038558	142.322	2.30297	130.103	150.65	1
Ak4	0.133599	0.0266616	130.091	2.49879	113.296	120.907	1
Wdr82	0.0345712	0.0375697	120.284	4.75639	247.435	138.724	1
Smim15	0.0648593	0.0122298	112.96	3.61512	109.421	80.7139	1
Bmpr1a	0.14779	0.0402533	100.478	2.35715	117.732	133.192	1
Nras	0.0876828	0.016957	99.3728	3.12628	102.911	87.7815	1
Ncoa4	0.13538	0.026847	95.1345	2.48002	98.7792	106.213	1
Zfp36l1	0.189447	0.0482643	94.7048	2.02203	108.494	143.083	1
Rad23b	0.207222	0.0481592	87.8604	1.90538	98.7738	138.239	1
Grrp1	0.0696926	0.0120137	73.8665	3.49539	86.9946	66.3689	1
Kbtbd4	0.0121675	0.00181789	68.6078	7.08312	85.0688	32.0269	1
Ppp3cb	0.0122882	0.00186858	66.0839	7.0582	84.0417	31.7519	1
Xrcc2	0.0978391	0.017657	62.1416	2.95702	79.8851	72.0411	1
Frem1	0.190187	0.0394888	49.8474	2.01691	71.7084	94.8097	1
BC055324	0.0855623	0.0152405	31.032	3.16468	55.4952	46.7622	1
1700071K01Rik	0.0835977	0.0372407	28.7017	3.20133	81.4314	67.8314	1
Vcl	0.0410572	0.00888456	26.8157	4.42709	57.0106	34.3405	1
Dcbld2	0.00441472	0.000751619	26.0754	9.99464	53.6828	14.3231	1
Vdac3	0.037857	0.00729253	23.3679	4.58079	50.145	29.1915	1
Fam134a	0.0865669	0.0153191	21.2701	3.14634	43.8034	37.1254	1
Tmem168	0.0604595	0.0110186	19.0455	3.73414	42.0829	30.0528	1
Tmem260	0.209812	0.0444988	17.4207	1.8894	38.2469	53.9812	1
G2e3	0.14707	0.0341893	17.0232	2.36394	41.0468	46.3033	1
Asxl1	0.0187417	0.00282286	14.6717	6.04782	34.0352	15.0072	1
Lrp5	0.0744577	0.0128596	10.2077	3.38702	25.2834	19.9061	1
Fbln7	0.0771986	0.0134184	9.92805	3.3285	24.6916	19.782	1

Psme4	0.0622741	0.0117954	9.50534	3.68379	24.9285	18.0455	1
Galnt1	0.137452	0.040416	9.25907	2.45857	28.6823	31.1101	1
Zmym2	0.0550208	0.0123839	9.15435	3.89702	26.0156	17.8021	1
Fam135a	0.209284	0.0447887	9.14326	1.89264	23.0574	32.4871	1
Herc4	0.146904	0.0305809	8.5524	2.3655	22.2759	25.1119	1
Chp1	0.0679347	0.0116296	8.49087	3.53771	21.475	16.1875	1
Uqcc1	0.0390475	0.0062011	8.47144	4.52179	21.3934	12.6165	1
Tab3	0.130096	0.0254227	8.10374	2.5366	20.7706	21.8356	1
Gm9731	0.0559789	0.00942202	8.08578	3.86691	20.584	14.1949	1
Rnf13	0.0169685	0.00261649	8.05454	6.27636	20.7583	8.81969	1
Asun	0.158531	0.0344835	7.83342	2.2606	20.7887	24.5229	1
Zmpste24	0.0233574	0.00885319	6.73174	5.561	24.0373	11.5266	1
Naf1	0.176801	0.0418207	5.86393	2.11343	15.8568	20.0077	1
Casd1	0.0179322	0.00876344	5.76901	6.14865	22.3592	9.69718	1
Tgoln1	0.141135	0.0364453	5.76486	2.42139	16.5876	18.2679	1
Msx1os	0.105682	0.0201261	5.5666	2.8405	14.0596	13.1992	1
Gm16845	0.128806	0.0244357	5.29046	2.55085	12.9968	13.5869	1
Tmx2	0.125231	0.0264486	5.19021	2.59126	13.3925	13.7822	1
Tnfrsf19	0.000615231	7.80E-05	4.50234	18.2571	10.6757	1.55932	1
Rab2b	0.156881	0.0315588	4.46078	2.27491	10.5618	12.3807	1
AI597479	0.0958106	0.0173179	4.23012	2.98904	9.76643	8.7131	1
Kbtbd7	0.173579	0.0370431	3.86502	2.138	8.79676	10.9719	1
Cep57l1	0.137154	0.026552	3.77402	2.46162	8.29086	8.98146	1
Rfc1	0.152241	0.0363267	3.64333	2.31613	8.62832	9.93418	1
Zfp213	0.185483	0.0381806	3.60976	2.04986	7.72267	10.0464	1
Firre	0.106123	0.0410985	3.53625	2.83428	10.7438	10.1085	1
Ascc3	0.0857679	0.0153479	3.51273	3.16091	7.4377	6.27474	1
Pnpla8	0.0149736	0.00377884	3.40451	6.572	8.52527	3.45923	1
Tmem65	0.212261	0.0488379	3.29644	1.8745	6.95551	9.8949	1
Crbn	0.047208	0.0133115	3.22665	4.16965	8.02938	5.13512	1
Styx	0.181553	0.0494472	3.20594	2.07818	7.40618	9.50341	1
Srgap2	0.104257	0.0226815	3.17922	2.86086	6.78748	6.32674	1
Abcc4	0.21977	0.0471835	3.11245	1.83011	6.03723	8.79691	1
Morc4	0.111806	0.0206899	3.1096	2.7567	6.03342	5.83637	1
Abcb7	0.0131108	0.00420375	2.97254	6.89619	7.34951	2.84196	1
Abcd2	0.162406	0.0323636	2.96527	2.22771	5.53379	6.6242	1
1810030O07Rik	0.110112	0.0267214	2.93423	2.7793	6.16771	5.91776	1
Tgs1	0.0949088	0.0178269	2.9085	3.00354	5.44685	4.83593	1
Mir17hg	0.0885933	0.0169221	2.8517	3.11014	5.31096	4.55368	1
Uba6	0.0482404	0.00786145	2.71941	4.13053	4.69103	3.02853	1
Unc5c	0.127359	0.0241897	2.71217	2.56703	4.66951	4.85076	1
Zmym5	0.0407581	0.00894538	2.53129	4.44081	4.57218	2.74555	1
Slc12a2	0.155873	0.0396825	2.46766	2.28373	4.34663	5.07547	1
Phf8	0.0278373	0.0287789	2.35827	5.19141	7.58375	3.89554	1
Ankrd6	0.107606	0.0197433	2.21635	2.81354	2.96704	2.81216	1

Dcaf10	0.0949676	0.0171257	2.20289	3.00259	2.92757	2.60004	1
Tpd52	0.188636	0.0389342	2.18361	2.02767	2.85651	3.75672	1
Ndrg3	0.191492	0.042591	2.16591	2.00795	2.90404	3.85673	1
Lypd6	0.0520655	0.00854808	2.16158	3.99414	2.78266	1.85783	1
Xrcc5	0.107949	0.0418802	2.09547	2.80879	3.68901	3.50234	1
Slc35e2	0.0188403	0.0037914	2.0713	6.0359	2.76944	1.22354	1
Atp6ap2	0.13404	0.0281839	2.02098	2.49411	2.42581	2.59364	1
Skil	0.00146504	0.0239759	-2.09323	14.1171	14.0182	2.64797	1
Phf21a	0.000777518	0.0112083	-4.11657	17.0504	44.6159	6.9779	1
Plcxd1	2.63E-05	0.00606982	-68.0695	43.7162	800.497	48.8299	1
Nle1	0.00417059	0.0481801	-7357.71	10.1807	2088.97	547.171	1
Flnc	0.00974143	0.0485896	-24191.3	7.66069	2029.69	706.529	1
Gtpbp6	0.000111698	0.00648578	-1.32E+06	29.5448	6182.27	558.002	1
Llgl2	0.00110059	0.0333734	-2.56E+06	15.3843	7151.51	1239.62	1
B3galnt2	0.000414672	0.0156059	-2.60E+06	20.457	6701.82	873.612	1
Sqle	0.000624741	0.0358134	-3.09E+09	18.1758	19201.1	2817.09	1
Creld2	0.0153845	0.0411483	-8.29E+09	6.50724	8069.96	3307.07	1
Atat1	0.0218027	0.0381971	-1.66E+10	5.71049	7230.5	3376.48	1
Ptpn23	0.037163	0.0402542	-2.19E+14	4.61626	11831.2	6834.51	1
B2m	0.00822463	0.0254066	-3.01E+14	8.12207	16878.2	5541.52	1
Sdhb	0.00208721	0.0223985	-1.10E+32	12.6691	121592	25593.3	1
Txndc12	0.000998452	0.010323	-2.15E+34	15.8368	125086	21062.5	1
Igf2bp1	0.113429	0.0396525	-3.38E+50	2.73542	86342.6	84172.3	1
Osbpl11	0.0559581	0.0318476	-8.05E+52	3.86756	119713	82541.4	1
Cenpa	0.000349386	0.0236438	-6.11E+53	21.4819	595682	73945.3	1
Tcfl5	4.97E-05	0.0144888	-2.14E+56	36.8302	901195	65250.5	1
