# Supplementary material for: Single-Cell Expression Profiling Reveals a Dynamic State of Cardiac Precursor Cells in the Early Mouse Embryo
Source: PLoS One. 2015 Oct 15;10(10):e0140831. doi: 10.1371/journal.pone.0140831 (PMC4607431; doi:10.1371/journal.pone.0140831)
Supplement: S7 Table — (PDF) [file pone.0140831.s017.pdf]

Table S7. The enriched genes in EHF *Nkx2-5*$^+$ CPs filtered via ANOVA

ID	p-value (Attribute)	p-value	Fold-Change	F (Attribute)	SS (Attribute)	SS (Error)	F (Error)
Eef1b2	0.025648	0.00786499	8.99E+307	5.36197	5.44E+06	2.71E+06	1
Tubb5	0.0335087	0.0140217	8.99E+307	4.81766	8.11E+06	4.49E+06	1
Tpt1	0.0366609	0.0298859	8.99E+307	4.64244	6.02E+06	3.46E+06	1
Gnas	0.127941	0.0299958	8.99E+307	2.56049	4.33E+06	4.51E+06	1
Rplp2	0.0479111	0.00818058	1.69E+213	4.14289	1.15E+06	740839	1
Apoe	0.0730081	0.0137972	2.17E+169	3.41906	740500	577547	1
Gins2	0.148523	0.0295836	2.05E+160	2.35029	644170	730882	1
Ndufc2	0.0782143	0.0185841	4.49E+156	3.30746	697361	562254	1
Cenpa	0.000349386	4.45E-05	8.82E+153	21.4819	595682	73945.3	1
Aprt	0.0939343	0.0181957	2.64E+152	3.01941	597090	527335	1
Nhp2	0.155904	0.0319203	9.46E+148	2.28346	561062	655219	1
Tmbim6	0.144131	0.0450942	7.29E+148	2.39202	701674	782238	1
Shfm1	0.151276	0.0300481	2.57E+136	2.3249	464888	533227	1
Chsy1	0.113648	0.0226225	2.03E+121	2.73259	377636	368526	1
Polr2e	0.0147948	0.00284704	6.80E+110	6.60089	336355	135883	1
Rps21	0.0588534	0.00982222	3.08E+110	3.78028	303130	213832	1
Sdhc	0.0173861	0.00306059	1.88E+105	6.21994	293242	125721	1
Rpl12	0.119203	0.0241544	3.60E+97	2.66269	245339	245705	1
Klhdc3	0.184941	0.0434048	2.81E+89	2.05373	213097	276696	1
Bola3	0.128955	0.024777	2.31E+82	2.54919	169428	177236	1
Sod1	0.00111641	0.000177259	3.90E+81	15.3189	176705	30760.2	1
Ndufc1	0.0329048	0.00551285	7.69E+77	4.85358	154799	85050.1	1
Rps13	0.114737	0.0218197	5.74E+74	2.71856	140341	137662	1
Rbm8a	0.0923157	0.0215989	1.88E+73	3.04622	150371	131635	1
Tars	0.0470861	0.0283806	1.61E+66	4.17434	191244	122171	1
Sdhb	0.00208721	0.000611622	6.57E+61	12.6691	121592	25593.3	1
Snrpn	0.06576	0.0127242	4.30E+61	3.59199	99621.8	73958.6	1
Mrpl52	0.0271078	0.00462758	2.57E+60	5.24633	94382.5	47973.8	1
Fundc1	0.114803	0.0213031	1.04E+60	2.71772	89441.2	87761	1
Pafah1b3	0.136129	0.03066	1.08E+56	2.47222	84239.8	90865.6	1
Snurf	0.0485164	0.00912443	2.67E+51	4.12024	69503.6	44983.5	1
Runx1t1	0.195693	0.0489293	4.23E+50	1.97958	70222.2	94595.5	1
Gm12504	0.0864787	0.016534	4.01E+45	3.14794	53430.2	45261.5	1
Mrps22	0.108693	0.0217928	2.21E+44	2.79857	50803.6	48409.2	1
Jagn1	0.187148	0.0429421	2.15E+43	2.03809	49347	64566.5	1
Ap1s1	0.137946	0.0276726	6.45E+42	2.45351	46428.4	50462	1
Lyplal1	0.134277	0.0258604	1.57E+40	2.4916	40243.7	43071.3	1
Rbm45	0.194934	0.040643	3.37E+35	1.98465	31374.5	42156.3	1
Acvr2b	0.0550394	0.0114514	1.95E+34	3.89643	32024	21916.8	1
Ecsit	0.0946987	0.0231625	4.49E+33	3.00695	32330.4	28671.7	1
Gins4	0.145091	0.0334664	1.94E+32	2.38278	28156.1	31510.6	1
Tmem179b	0.186573	0.04036	4.93E+31	2.04214	25595.8	33423.5	1

Cdc42ep5	0.0217371	0.0033452	2.01E+30	5.71708	23040.2	10746.8	1
Glcci1	0.171665	0.0351844	1.38E+30	2.15286	22735.7	28161.8	1
Atf7ip	0.125543	0.0237009	3.92E+28	2.58767	20301.8	20921.6	1
Ppp2r5a	0.0858899	0.0456127	3.40E+28	3.15867	34247.5	28913	1
Sumf2	0.122136	0.0287808	9.38E+27	2.62739	21633.1	21956.5	1
Trmt1	0.108075	0.0233801	8.80E+27	2.80706	20902.2	19856.8	1
Slc25a51	0.144753	0.0325503	2.96E+27	2.38602	20129.5	22497.1	1
Serf1	0.0980473	0.0176868	7.21E+26	2.95378	17910.7	16169.7	1
Ptdss2	0.139055	0.0303033	7.16E+25	2.44224	17616.6	19235.4	1
Cby1	0.0621911	0.01046	2.36E+23	3.68605	13566.1	9814.39	1
Zscan12	0.134589	0.0258262	1.34E+22	2.48832	12171.9	13044.3	1
Apopt1	0.104696	0.0191192	8.98E+17	2.85456	8005.9	7478.93	1
Prdx5	0.060705	0.012322	7.48E+17	3.72722	8578.46	6137.53	1
Atat1	0.0218027	0.00491793	7.07E+15	5.71049	7230.5	3376.48	1
Smarcc1	0.202	0.0427462	3.23E+15	1.93835	5996.8	8250.02	1
Polr2i	0.137077	0.0264654	7.21E+14	2.46242	5494.44	5950.19	1
Galt	0.10337	0.0309009	3.18E+14	2.87371	6585.43	6110.97	1
Nudt14	0.165659	0.0364427	2.00E+14	2.20083	5327.93	6455.66	1
Gm5779	0.0844745	0.0174085	2.63E+13	3.18484	4788.11	4009.09	1
Dimt1	0.167054	0.0344366	8.92E+12	2.1895	4222.71	5142.97	1
Dnajc4	0.175074	0.0436233	6.88E+12	2.12653	4557.19	5714.7	1
Kif3a	0.17415	0.0358455	6.22E+12	2.13361	4098.7	5122.72	1
Mpnd	0.0654805	0.0112523	4.52E+12	3.59912	4000.43	2964.01	1
Zgpat	0.0537506	0.00886125	2.88E+12	3.93795	3854.05	2609.85	1
Tenc1	0.0405181	0.00645177	1.38E+12	4.45192	3659.83	2192.21	1
Vapb	0.0867174	0.0157034	3.89E+11	3.14362	3370.6	2859.21	1
Cox20	0.166529	0.0363527	2.84E+11	2.19375	3401.4	4134.65	1
Pogz	0.0810497	0.0141928	2.10E+11	3.25047	3185.6	2613.45	1
2310009B15Rik	0.152022	0.0427863	2.80E+10	2.31811	3256.5	3746.15	1
Krt10	0.141921	0.0298756	7.77E+09	2.41361	2530.04	2795.3	1
Stard3	0.158394	0.0454158	5.81E+09	2.26178	2865.22	3378.13	1
Dopey2	0.113333	0.0209794	1.57E+09	2.73667	2100.04	2046.32	1
Alg11	0.153428	0.0302612	1.31E+09	2.30544	2066.71	2390.53	1
Klhl31	0.0968343	0.0175039	3.07E+08	2.97278	1791.69	1607.2	1
Prickle3	0.166865	0.0361616	2.43E+08	2.19103	1816.51	2210.84	1
Zak	0.199719	0.0424769	4.07E+07	1.95308	1449.66	1979.32	1
Amph	0.22414	0.04864	2.13E+07	1.8051	1337.11	1975.3	1
Zscan21	0.147848	0.0289717	6.91E+06	2.35661	1163.75	1316.86	1
Foxp2	0.00258989	0.000338018	6.49E+06	11.847	1152.82	259.49	1
Nxf1	0.0259939	0.00418038	1.11E+06	5.33384	925.658	462.785	1
Pnma2	0.05574	0.00923318	762988	3.87436	859.19	591.369	1
Gm12669	0.053631	0.00883894	414553	3.94187	783.541	530.064	1
Matk	0.0916504	0.016345	412690	3.05741	782.995	682.928	1
Emc2	0.150474	0.0295178	73990.5	2.33224	589.065	673.532	1
Gm11517	0.0664069	0.0166078	50255.6	3.57561	646.23	481.953	1

Msl1	0.0434682	0.00752141	28959.2	4.3208	508.718	313.965	1
B630005N14Rik	0.035996	0.00591474	12429.4	4.67781	423.281	241.299	1
Ptp4a3	0.110431	0.0203475	6855.14	2.77501	365.383	351.118	1
0610009O20Rik	0.109351	0.0214616	4888.48	2.7896	348.044	332.706	1
Dpysl2	0.128006	0.0243445	2665.43	2.55976	291.903	304.094	1
Smpd1	0.0530114	0.00872609	2227.97	3.96233	278.329	187.317	1
Rbfa	0.201701	0.044498	1405.83	1.94027	252.693	347.296	1
Alg2	0.086984	0.0182279	1360.66	3.1388	262.576	223.08	1
Nfia	0.123605	0.0238963	1020.07	2.6101	227.589	232.521	1
Rad51d	0.0957095	0.0171958	1009.59	2.99066	224.092	199.815	1
Fgf8	0.092801	0.0181193	926.558	3.03812	227.205	199.426	1
Sult5a1	0.0521285	0.00855977	809.889	3.992	210.029	140.3	1
Prex2	0.0697125	0.0119731	766.866	3.49492	207.048	157.98	1
Zfp462	0.0406566	0.00648808	708.483	4.4455	201.86	121.087	1
4921517D22Rik	0.138911	0.0269066	693.23	2.4437	200.92	219.252	1
3000002C10Rik	0.0674075	0.0161326	549.53	3.55067	215.256	161.664	1
Tef	0.0688456	0.0124008	427.124	3.51562	175.723	133.29	1
Lenep	0.0901112	0.0165226	383.661	3.08368	167.991	145.273	1
Mfn2	0.0640353	0.0109411	373.638	3.63663	165.114	121.074	1
Mir692-2a	0.0818877	0.0151918	274.22	3.2341	151.272	124.731	1
Mir692-2b	0.0818877	0.0151918	274.22	3.2341	151.272	124.731	1
Gm6981	0.120428	0.0364766	230.637	2.64783	175.054	176.3	1
Sp8	0.137978	0.0266039	143.442	2.45318	115.617	125.679	1
Hnrnpl	0.0129624	0.00208971	129.482	6.92448	115.288	44.3984	1
Ahdc1	0.14526	0.0288495	100.992	2.38116	100.774	112.858	1
Ggcx	0.160419	0.0318956	88.5792	2.24446	94.1913	111.91	1
Nacc2	0.102572	0.0187101	77.0381	2.8854	88.5133	81.8035	1
Cul2	0.083056	0.0148532	45.69	3.21162	68.9543	57.254	1
Btg1	0.0207721	0.00337126	34.8926	5.8171	60.9272	27.9302	1
Armcx4	0.193618	0.04251	34.7156	1.9935	60.6754	81.1645	1
Qrich1	0.0503516	0.0144146	28.6103	4.05366	66.1683	43.5283	1
Rapgef4	0.219869	0.0472344	27.2092	1.82953	51.1228	74.5149	1
5830416I19Rik	0.19317	0.0401571	24.8518	1.99652	48.3782	64.6166	1
Gm5177	0.115021	0.0308994	23.9531	2.71492	56.2761	55.2758	1
Ggt5	0.153478	0.0302293	23.207	2.30499	46.3299	53.5995	1
Celf2	0.0783659	0.0137239	18.9389	3.30435	40.6304	32.7895	1
Plau	0.166172	0.0332777	18.2735	2.19665	39.5329	47.9917	1
Ncan	0.156159	0.0308398	16.8351	2.28122	37.3333	43.6415	1
Gm17066	0.0998289	0.0184227	16.7256	2.92639	37.4857	34.1587	1
Caprin2	0.0604481	0.0101233	14.4429	3.73446	33.3905	23.8432	1
Spry2	0.0720509	0.0143556	13.2775	3.44065	33.335	25.8362	1
Cd63	0.132665	0.0254963	11.8974	2.50874	28.8139	30.6277	1
Gm10364	0.0692728	0.0241869	11.426	3.50538	38.0037	28.9107	1
Fmr1	0.168846	0.0456749	11.0691	2.17512	31.6068	38.7495	1
Unc13c	0.0251949	0.00381892	10.6361	5.39954	26.1846	12.9318	1

Slitrk2	0.129172	0.0247709	10.3453	2.54679	25.6899	26.8991	1
Sarm1	0.146295	0.0292616	9.92307	2.37128	24.9861	28.0986	1
Ccar1	0.0358507	0.0100988	9.68223	4.68564	30.2563	17.2193	1
Fam227a	0.0707845	0.0124727	9.56594	3.46977	24.1642	18.5712	1
Fgf10	0.202461	0.042629	9.46025	1.9354	23.675	32.6203	1
Atxn1	0.159939	0.031875	9.24299	2.24854	23.2042	27.5192	1
Cstf2t	0.137383	0.0300397	9.13334	2.45928	24.3845	26.4409	1
Mir3063	0.082209	0.0150263	9.07063	3.22788	23.1838	19.153	1
Mapt	0.0572339	0.00959975	8.45349	3.82843	21.4146	14.9162	1
Zfp937	0.123324	0.0238864	7.61305	2.61339	19.5604	19.9592	1
Gtf2a1	0.13817	0.0270003	7.53989	2.45123	19.2546	20.9469	1
C5ar2	0.210917	0.0448075	6.24302	1.88265	15.7122	22.2554	1
Prune2	0.222405	0.0479242	6.02348	1.81496	15.1035	22.1912	1
Acta1	0.193764	0.0440983	5.90403	1.99251	15.4971	20.7405	1
Itgav	0.0546974	0.00907567	5.66173	3.90733	14.0997	9.62275	1
Mc1r	0.220682	0.0477681	5.42856	1.82484	13.4537	19.6601	1
Igsf10	0.128774	0.0264996	5.37072	2.5512	13.7512	14.3736	1
Kcnj2	0.111983	0.0207227	5.05135	2.75436	12.2956	11.9041	1
Gm8300	0.0208734	0.00313643	4.99804	5.80634	12.1651	5.58705	1
D030040B21Rik	0.156747	0.0311509	4.87287	2.27607	11.777	13.7981	1
Vps54	0.130455	0.0423445	4.59156	2.53267	14.2037	14.9552	1
Gm2022	0.0516722	0.00857066	4.50403	4.00758	10.6536	7.08898	1
Zhx2	0.123315	0.0234663	4.44109	2.61349	10.4658	10.6788	1
Tceb3	0.000278062	3.38E-05	4.12731	22.9178	9.41522	1.09553	1
Gm5662	0.0270988	0.00429733	3.99748	5.24702	9.11891	4.63445	1
Pfn2	0.221735	0.049873	3.88401	1.81879	8.8344	12.9528	1
4933415F23Rik	0.144184	0.0293937	3.74196	2.39152	8.35065	9.31141	1
Itpr2	0.140845	0.0272278	3.70846	2.42428	8.04706	8.85163	1
Gm2016	0.0168614	0.0025393	3.57294	6.2911	7.6725	3.25221	1
BC006965	0.116076	0.0217053	3.49341	2.70153	7.34653	7.25172	1
Gas7	0.186038	0.0385018	3.32343	2.04592	6.77663	8.8327	1
Cd59a	0.190415	0.0400814	3.31823	2.01534	6.79897	8.9963	1
Hoxc8	0.0954161	0.0171404	3.2802	2.99536	6.60978	5.88445	1
Bean1	0.12928	0.024579	3.27283	2.5456	6.58793	6.90126	1
Adam23	0.0562313	0.00969613	3.22764	3.85909	6.53061	4.51272	1
Gm4925	0.00473921	0.000836072	3.14532	9.7663	6.6979	1.82885	1
Npas3	0.0814758	0.0144268	2.97019	3.24212	5.578	4.58794	1
4921504A21Rik	0.0798392	0.014087	2.93434	3.2745	5.4533	4.44103	1
Jmjd1c	0.193214	0.049712	2.91933	1.99623	6.03414	8.06072	1
Shank1	0.0910982	0.0163701	2.83747	3.06677	5.11263	4.44562	1
Hmgn5	0.16717	0.041128	2.6779	2.18857	5.04764	6.15031	1
Mbnl1	0.173493	0.0351421	2.56857	2.13866	4.17051	5.20015	1
Exoc8	0.149586	0.0293909	2.25895	2.34043	3.11628	3.55068	1
Piwil1	0.0582056	0.00970613	2.25103	3.79934	3.0842	2.16473	1
Fgf9	0.0111872	0.00162116	2.14457	7.29755	2.74686	1.00375	1

Nrgn	0.191463	0.0396644	2.13901	2.00814	2.70756	3.59545	1
Prlr	0.113069	0.0210876	2.13496	2.74011	2.7036	2.63114	1
Foxn3	0.0954146	0.0182118	2.07605	2.99539	2.56695	2.28524	1
Pnpla8	0.0149736	0.0479899	-2.03134	6.572	8.52527	3.45923	1
Abcb7	0.0131108	0.0305663	-2.05874	6.89619	7.34951	2.84196	1
Ncapg2	0.00504723	0.014633	-2.78209	9.5669	14.6289	4.07764	1
Kdm5b	0.0785463	0.0230603	-2.95941	3.30065	6.94374	5.60999	1
Zwint	0.100153	0.0496276	-4.38652	2.92148	16.8034	15.3378	1
Cnot1	0.139895	0.0389097	-78.5457	2.43378	107.005	117.244	1
Nup153	0.0487988	0.0442872	-93.8953	4.1098	209.647	136.031	1
Wdr82	0.0345712	0.0459202	-93.9051	4.75639	247.435	138.724	1
Plcxd1	2.63E-05	0.0031368	-116.368	43.7162	800.497	48.8299	1
Ttc3	0.0164571	0.0382253	-711.896	6.34786	626.475	263.175	1
Map3k12	0.0986121	0.0397962	-1720.17	2.94503	381.894	345.798	1
Nle1	0.00417059	0.0492306	-6979.27	10.1807	2088.97	547.171	1
Gtpbp6	0.000111698	0.0133196	-200746	29.5448	6182.27	558.002	1
Tfip11	4.02E-05	0.00179274	-219476	39.003	3944.69	269.702	1
Ptges3	0.00347274	0.0300975	-1.24E+06	10.7979	4314.29	1065.47	1
B3galnt2	0.000414672	0.0138117	-3.84E+06	20.457	6701.82	873.612	1
Llgl2	0.00110059	0.0290818	-4.26E+06	15.3843	7151.51	1239.62	1
Gm15645	0.00645119	0.0294896	-1.38E+07	8.81885	4785.02	1446.91	1
Sf3b3	0.00664393	0.049463	-2.03E+07	8.73214	6493.55	1983.03	1
Cse1l	0.0451544	0.0367319	-9.07E+07	4.25073	3198.68	2006.67	1
Sub1	0.0292014	0.0381163	-2.43E+08	5.09336	4337.61	2270.99	1
Sqle	0.000624741	0.0373006	-2.46E+09	18.1758	19201.1	2817.09	1
Itga5	0.00539418	0.0233888	-3.03E+09	9.35975	8023.7	2286.02	1
Fabp5	0.00395384	0.0344306	-4.91E+09	10.3577	11185.3	2879.75	1
Metap1	0.00133591	0.00870679	-8.61E+10	14.516	10868.2	1996.54	1
B2m	0.00822463	0.0468814	-2.50E+12	8.12207	16878.2	5541.52	1
Mdn1	0.00588872	0.0447453	-7.19E+13	9.0916	23010.6	6749.25	1
Chaf1a	0.00332378	0.0271499	-5.57E+14	10.9499	24359.8	5932.45	1
Plekhg2	0.0552302	0.0453887	-6.81E+14	3.89038	11370.5	7793.95	1
Tmem132a	0.0966506	0.0333502	-6.08E+17	2.97568	10648.7	9542.91	1
Exosc6	0.0476933	0.0447066	-2.22E+20	4.15113	22639.9	14543.8	1
Arrb1	0.0297364	0.0258833	-6.83E+26	5.05645	36414.8	19204.4	1
Abhd17a	0.143918	0.0296922	-9.71E+27	2.39409	20035.2	22316.2	1
Cited2	0.0363344	0.0474645	-2.36E+30	4.65971	58512.5	33485.7	1
Mrpl30	0.0682996	0.0355601	-5.10E+31	3.52882	41467.5	31336.3	1
Stk4	0.00726592	0.0449873	-3.53E+34	8.4724	133689	42078.4	1
Bex2	0.0206182	0.0167279	-3.17E+35	5.83359	60310.6	27569.4	1
Cnih1	6.21E-05	0.00187869	-6.99E+37	34.6865	178882	13752.3	1
Rab34	0.010594	0.00705815	-1.23E+40	7.43905	69005.6	24736.3	1
E430025E21Rik	0.00129985	0.0194234	-3.17E+40	14.6361	210382	38331.1	1
Rnps1	0.0730318	0.0246415	-1.14E+45	3.41853	67819.2	52903.3	1
Eif4g2	0.0164317	0.0326221	-3.67E+45	6.35148	147575	61959.1	1

Psmc2	0.0836652	0.0423767	-1.59E+56	3.20005	129410	107840	1
Eftud2	0.0278309	0.0099119	-6.86E+61	5.19188	130879	67222.2	1
Lsm8	0.00701224	0.0220096	-2.60E+64	8.57486	329940	102607	1
Dnajc8	0.016931	0.0167719	-5.77E+64	6.28151	216359	91850.2	1
Larp1	0.122716	0.0346213	-3.80E+65	2.62053	129949	132237	1
Tcfl5	4.97E-05	0.00289014	-4.01E+76	36.8302	901195	65250.5	1
Smad4	0.0108351	0.0123121	-4.45E+88	7.38039	417700	150922	1
Odc1	0.00537638	0.0191304	-1.31E+143	9.36997	1.67E+06	475382	1
Hand1	0.00431322	0.0428105	-3.95E+178	10.0704	4.14E+06	1.09E+06	1
Hmga1-rs1	0.153029	0.0485581	-1.05E+200	2.30902	1.27E+06	1.47E+06	1
Pcbp1	0.0312566	0.0486932	-8.99E+307	4.95592	1.27E+07	6.83E+06	1
Rpl14-ps1	0.167213	0.0461085	-8.99E+307	2.18822	5.13E+06	6.25E+06	1
Hsp90ab1	0.181112	0.0445305	-8.99E+307	2.0814	1.15E+07	1.48E+07	1
