# Supplementary material for: Single-Cell Expression Profiling Reveals a Dynamic State of Cardiac Precursor Cells in the Early Mouse Embryo
Source: PLoS One. 2015 Oct 15;10(10):e0140831. doi: 10.1371/journal.pone.0140831 (PMC4607431; doi:10.1371/journal.pone.0140831)
Supplement: S8 Table — (PDF) [file pone.0140831.s018.pdf]

Table S8. The enriched genes in EHF *Nkx2-5*$^{+}$/*Tbx5*$^{+}$ CPs filtered via ANOVA

ID	p-value (Attribute)	p-value	Fold-Change	F (Attribute)	SS (Attribute)	SS (Error)	F (Error)
H19	0.0569123	0.00950099	8.99E+307	3.83819	3.72E+06	2.58E+06	1
Rpl41	0.155844	0.0366003	8.99E+307	2.28399	6.08E+06	7.10E+06	1
Gm13826	0.144442	0.0338512	5.95E+283	2.38902	2.19E+06	2.45E+06	1
Gata6	0.216915	0.0497295	9.13E+272	1.84677	1.92E+06	2.77E+06	1
Zyx	0.154911	0.0310491	1.04E+233	2.29222	1.36E+06	1.58E+06	1
Serpinh1	0.0320636	0.00504435	4.38E+200	4.90499	1.00E+06	546182	1
D8Ertd738e	0.203561	0.0446916	3.08E+186	1.92839	883468	1.22E+06	1
Myl7	0.16521	0.0351317	3.01E+184	2.2045	871499	1.05E+06	1
Ppib	0.0108637	0.00158591	1.53E+182	7.37352	833016	301264	1
Nisch	0.0698764	0.0182939	1.05E+179	3.49105	954586	729169	1
Taf6	0.123932	0.0234143	1.12E+165	2.60628	677454	693149	1
Calr	0.048646	0.011322	4.21E+160	4.11544	738737	478677	1
Ilkap	0.104594	0.0193229	4.53E+141	2.85602	501033	467814	1
Lman1	0.0879558	0.0163756	8.79E+127	3.12142	415293	354790	1
Cdkn1c	0.100038	0.0188298	8.68E+118	2.92322	357381	326016	1
Actb	0.103432	0.0207487	7.93E+116	2.87281	354398	328968	1
Rpsa	0.136357	0.0485	1.10E+115	2.46986	450230	486107	1
Lphn1	0.0796458	0.014263	5.61E+114	3.27837	330656	268959	1
Smarcd3	0.0597487	0.0102569	4.22E+111	3.75437	312675	222088	1
Ykt6	0.0678679	0.0129048	9.67E+109	3.53935	314431	236903	1
Tuba1a	0.113028	0.0370734	3.67E+109	2.74065	392817	382214	1
Sec61a1	0.0279277	0.0184547	2.03E+89	5.18473	354191	182172	1
Sfrp5	0.193338	0.040145	2.43E+88	1.99539	193970	259224	1
Snhg8	0.1011	0.0190538	2.77E+87	2.90723	193122	177142	1
Men1	0.147069	0.0447022	3.37E+85	2.36394	227822	256997	1
Pth1r	0.170306	0.0348458	7.78E+83	2.16354	176144	217106	1
Csrp2	0.0245826	0.00775224	1.16E+82	5.45167	219701	107466	1
Polr2m	0.0161916	0.00312651	4.97E+81	6.38608	182671	76278.9	1
Smad4	0.0108351	0.0238417	4.91E+76	7.38039	417700	150922	1
Gcn1l1	0.165378	0.0332543	3.02E+75	2.20312	141826	171666	1
Fam53b	0.169297	0.0340881	1.29E+75	2.17152	140158	172116	1
Bex1	0.139263	0.0281682	1.04E+75	2.44013	143004	156280	1
Mgrn1	0.0918325	0.0168775	9.16E+73	3.05434	137596	120132	1
Rps6ka2	0.0221347	0.00332987	6.48E+72	5.67747	131882	61944.2	1
Txndc12	0.000998452	0.000138461	9.46E+69	15.8368	125086	21062.5	1
H2-Ke2	0.161506	0.0376835	4.96E+69	2.23526	130734	155965	1
Crlf3	0.140764	0.0276129	1.55E+69	2.42509	119751	131680	1
Fasn	0.102906	0.0351423	2.85E+68	2.88049	156824	145183	1
Brd4	0.106356	0.0225225	3.52E+66	2.83098	117498	110679	1
Mrpl38	0.173222	0.0380724	6.81E+65	2.14076	112307	139896	1
Myl4	0.123351	0.0238202	7.87E+59	2.61307	90169.5	92018.9	1
Pdlim7	0.0428356	0.00704502	1.19E+59	4.34797	87507.1	53669.2	1

Timm9	0.143646	0.0299035	4.46E+57	2.39673	85396.4	95014.3	1
Gata4	0.0684458	0.0158957	1.65E+57	3.52527	92605.6	70050.8	1
Nelfcd	0.201689	0.0432474	4.08E+55	1.94035	77667.2	106740	1
Usf1	0.204881	0.0474519	3.61E+53	1.92003	74950.9	104097	1
Csnk2a2	0.18407	0.0432005	4.75E+52	2.05996	73941.8	95719.5	1
Dmap1	0.115748	0.0237771	1.32E+50	2.70568	65320.3	64378.5	1
Cpt2	0.0839007	0.0147569	3.90E+47	3.19561	56249.5	46939.1	1
Dctn1	0.0489623	0.00813183	8.09E+45	4.10378	52724.7	34260.9	1
Pomgnt1	0.162542	0.0350205	1.43E+44	2.22657	50349.7	60301.6	1
Fbn2	0.104365	0.0190345	7.85E+43	2.85931	47841.7	44618.5	1
Fbxo21	0.12126	0.0315129	5.70E+43	2.63783	55557.3	56164.6	1
Atad3a	0.068331	0.015993	2.95E+42	3.52806	51194.1	38694.8	1
Myl6	0.0432082	0.00695451	4.27E+41	4.33191	43084.8	26522.4	1
Ror2	0.0543599	0.00897578	2.37E+41	3.91817	42507.2	28930	1
Brms1	0.174918	0.0370125	1.66E+41	2.12773	43128.6	54052.7	1
Igf2r	0.0364375	0.00593618	7.03E+39	4.65424	39941.4	22884.6	1
Pdxp	0.0858889	0.0156073	2.60E+39	3.15869	39062.3	32977.6	1
Arhgef1	0.0263939	0.00444532	1.87E+38	5.30189	37728.2	18976	1
Tollip	0.0672632	0.0116122	1.86E+37	3.55424	34705.7	26038.9	1
Tcof1	0.173305	0.0463178	7.04E+36	2.14012	39025.7	48627.5	1
Ccdc50	0.0705338	0.039466	1.96E+36	3.47561	56458.2	43317.7	1
Dync1h1	0.0152123	0.00290494	5.87E+35	6.53412	34920.9	14251.7	1
Timm50	0.0720226	0.0171887	2.54E+35	3.44129	35807.2	27747.1	1
Prpf19	0.026932	0.00564596	3.69E+34	5.25984	33338.6	16902.2	1
Efhd2	0.0817766	0.0146938	9.53E+33	3.23626	28985.6	23884	1
Zfpl1	0.0350776	0.00587906	4.99E+33	4.72801	28914	16307.9	1
Ap3m1	0.152141	0.0299257	2.78E+33	2.31704	27794.3	31988.3	1
Kansl2	0.0721097	0.0458007	6.45E+32	3.43931	49421.6	38319	1
Kdm1a	0.123163	0.0263624	5.32E+32	2.61527	28258.1	28813.4	1
Aimp1	0.226912	0.0492946	1.49E+31	1.78955	24167.2	36012.4	1
Mdn1	0.00588872	0.000914272	6.43E+29	9.0916	23010.6	6749.25	1
Fam173a	0.0096771	0.00138357	5.98E+29	7.67839	22157.6	7695.21	1
Acsf3	0.224472	0.0499779	7.58E+27	1.80323	19628	29026.5	1
Hbp1	0.0242966	0.00376892	3.54E+27	5.47658	19028	9265.15	1
Trim35	0.194776	0.0436704	3.38E+27	1.98571	19579.2	26293.5	1
Pla2g4b	0.119651	0.0223759	3.08E+27	2.65723	18761.3	18827.9	1
L3mbtl2	0.091025	0.0174585	2.73E+27	3.06802	19302.5	16777.4	1
Fmnl3	0.11395	0.0221105	2.78E+26	2.72867	17731.3	17328.4	1
Kat7	0.155081	0.0309187	1.17E+26	2.29071	16964.6	19748.9	1
Nsun5	0.124916	0.0404738	1.00E+26	2.59488	21920.7	22527.2	1
Aph1a	0.222698	0.0482712	8.28E+25	1.81329	16733.1	24608.2	1
Camsap1	0.0168534	0.00456435	5.70E+25	6.29221	20473.5	8676.75	1
Pdyn	0.0887268	0.0157391	3.97E+25	3.10779	16271.1	13961.6	1
Cyth2	0.120954	0.0279211	1.19E+25	2.6415	17224.6	17388.7	1
Chd2	0.015765	0.00264944	9.30E+24	6.44916	16268.6	6726.89	1

Foxp4	0.0925656	0.0165714	4.69E+24	3.04204	15127.3	13260.7	1
Fam160a2	0.175835	0.0373072	4.51E+24	2.12075	15441.1	19415.9	1
Rrm1	0.195446	0.0412452	4.48E+24	1.98123	15195.7	20453	1
Rab31	0.136957	0.0281667	3.92E+24	2.46366	15518	16796.8	1
Cyb5b	0.199073	0.0429435	5.89E+23	1.95728	14259.9	19428.2	1
Furin	0.0693275	0.0123136	3.33E+23	3.50407	13963.4	10626.4	1
Dgcr14	0.157348	0.0391164	1.57E+23	2.27083	14999.5	17614.1	1
Akap2	0.118105	0.0295858	1.52E+23	2.6762	15340	15285.4	1
Htra2	0.115436	0.0227682	8.22E+22	2.70964	13401.7	13189.2	1
Maged2	0.00696947	0.00096432	3.37E+22	8.59259	12634	3920.89	1
Hs3st6	0.0536731	0.00884676	3.31E+22	3.94049	12592.7	8521.9	1
Nup98	0.108901	0.0200562	1.81E+22	2.79572	12312.1	11743.8	1
Mcat	0.128122	0.0254591	1.63E+22	2.55846	12526.9	13056.6	1
Phrf1	0.103734	0.0200371	1.31E+22	2.86843	12470.1	11592.9	1
Gm11974	0.0786664	0.0147395	1.15E+22	3.2982	12470.1	10082.4	1
Smg5	0.147936	0.031131	6.96E+21	2.35579	12290	13911.8	1
Cenpt	0.0981238	0.0190387	2.35E+21	2.95259	11711.3	10577.2	1
Chd6	0.10605	0.0219201	1.29E+21	2.83531	11694.7	10999.2	1
Ptdss1	0.146959	0.0398495	8.61E+20	2.36498	12847.7	14486.6	1
Gdi1	0.218532	0.0469194	8.37E+20	1.8373	10878.5	15789.1	1
Fabp5	0.00395384	0.000584016	7.67E+20	10.3577	11185.3	2879.75	1
Popdc2	0.170618	0.0343765	7.15E+20	2.16107	10798.5	13324.9	1
Rai1	0.083242	0.0155781	5.33E+20	3.20808	10961.9	9111.95	1
Otulin	0.133811	0.0327621	2.38E+20	2.49654	11625.1	12417.4	1
Ube2s	0.1015	0.0315282	1.02E+20	2.90126	12781.4	11747.9	1
Pbrm1	0.0290049	0.00446367	3.41E+19	5.10713	9480.26	4950.08	1
Kdm3b	0.149463	0.0323105	2.96E+19	2.34156	9888.6	11261.5	1
Mex3a	0.0625485	0.016271	1.68E+19	3.67633	11013.7	7988.9	1
Prpf31	0.108849	0.0265038	1.61E+19	2.79643	10435	9950.81	1
Fbrs	0.15937	0.031733	7.18E+18	2.2534	8844.33	10466.4	1
Dynlt1c	0.20331	0.0444608	3.21E+18	1.92998	8683.85	11998.5	1
Khsrp	0.178178	0.039751	2.67E+18	2.1031	8839.8	11208.6	1
Tube1	0.0684873	0.0117394	2.55E+18	3.52427	8430.96	6379.36	1
Bcr	0.0916581	0.0163489	2.42E+18	3.05728	8392.67	7320.39	1
Mcm8	0.202076	0.044674	6.84E+17	1.93786	8118.43	11171.7	1
Hsdl1	0.0731741	0.0128912	6.57E+17	3.41535	7959.19	6214.45	1
Cdc14b	0.0861904	0.0152293	3.70E+17	3.15318	7666.18	6483.33	1
Itga5	0.00539418	0.000858366	3.33E+17	9.35975	8023.7	2286.02	1
Lrpap1	0.205634	0.0467173	2.90E+17	1.9153	7881.17	10972.9	1
Cyfip1	0.138101	0.0278373	2.76E+17	2.45193	7723.31	8399.72	1
Pmvk	0.183082	0.0437611	2.53E+17	2.06707	8154.64	10520.1	1
Tcerg1l	0.0707471	0.0129546	1.86E+17	3.47064	7614.12	5850.32	1
Creld2	0.0153845	0.00289557	1.74E+17	6.50724	8069.96	3307.07	1
Tnnc1	0.0313215	0.00484844	1.50E+17	4.95177	7324.48	3944.44	1
Zfp414	0.0737234	0.0145517	1.03E+17	3.40315	7611.17	5964.02	1

Abcf3	0.0621138	0.0104419	8.58E+16	3.68817	7119.98	5147.98	1
Llgl2	0.00110059	0.000144987	7.31E+16	15.3843	7151.51	1239.62	1
Rxrb	0.189502	0.043568	4.59E+16	2.02165	7292.89	9619.74	1
Psap	0.132749	0.025552	3.29E+16	2.50785	6800.66	7231.33	1
Igfbp5	0.113124	0.0209336	3.18E+16	2.73939	6762.13	6582.61	1
Zfp512	0.0663948	0.0115294	3.12E+16	3.57592	6818.67	5084.88	1
Pwwp2b	0.116373	0.0267621	2.39E+16	2.69779	7353.38	7268.54	1
Srpk1	0.223752	0.048363	1.95E+16	1.8073	6594.57	9730.27	1
B3galnt2	0.000414672	5.45E-05	1.86E+16	20.457	6701.82	873.612	1
Endov	0.107456	0.0218978	1.52E+16	2.81563	6820.72	6459.87	1
Pacs2	0.211658	0.0454496	1.49E+16	1.87815	6531.38	9273.48	1
Rgp1	0.10668	0.0203127	1.13E+16	2.82645	6511.94	6143.81	1
Pip5k1a	0.114976	0.0218648	1.01E+16	2.7155	6429.61	6313.98	1
Sf3b3	0.00664393	0.000982269	9.42E+15	8.73214	6493.55	1983.03	1
Soga1	0.0812679	0.0143503	5.18E+15	3.24619	6156.2	5057.17	1
1010001N08Rik	0.120935	0.024137	4.49E+15	2.64172	6263.41	6322.56	1
Me2	0.179627	0.0397382	8.49E+14	2.09233	5771.87	7356.23	1
Gm2a	0.0497616	0.00812288	6.94E+14	4.07473	5469.03	3579.15	1
Chd3	0.152455	0.0299756	6.73E+14	2.31419	5461.35	6293.16	1
Tsix	0.217917	0.0497679	6.67E+14	1.84089	5652.01	8187.36	1
Slc12a4	0.0222481	0.00352634	4.14E+14	5.66634	5412.76	2547.32	1
Fads1	0.0788369	0.0143136	7.85E+13	3.29473	4879.69	3949.5	1
Gusb	0.101092	0.0247258	6.55E+13	2.90734	5432.1	4982.42	1
Zfyve19	0.0329959	0.00980463	3.80E+13	4.84811	5868.76	3228.07	1
Med1	0.154872	0.030642	1.90E+13	2.29257	4385.45	5101.06	1
Exosc7	0.0455939	0.0344616	1.00E+13	4.233	8230.82	5185.18	1
Bub1b	0.133133	0.0262602	8.71E+12	2.50374	4221.86	4496.59	1
Rfxap	0.0918525	0.0194427	5.45E+12	3.054	4343.2	3792.36	1
Nudcd3	0.0880386	0.0157298	5.00E+12	3.11994	4018.79	3434.92	1
Patl1	0.0146015	0.00270583	3.66E+12	6.63259	4266.78	1715.48	1
Gars	0.219074	0.0470467	2.89E+12	1.83415	3857.51	5608.44	1
Thap7	0.139388	0.0270591	2.31E+12	2.43888	3808.53	4164.25	1
Elmo1	0.155265	0.0409371	2.23E+12	2.28909	4388.63	5112.52	1
Zbtb18	0.220579	0.0474048	1.96E+12	1.82544	3751.47	5480.29	1
Lmf2	0.0570857	0.0148321	1.53E+12	3.83292	4432.7	3083.95	1
Lamb2	0.177763	0.0363958	1.41E+12	2.1062	3676.09	4654.3	1
Whrn	0.0605851	0.0101493	9.96E+11	3.73059	3574.49	2555.08	1
Fntb	0.0428457	0.00891774	7.07E+11	4.34753	3858.71	2366.84	1
Gga2	0.0136872	0.00203425	6.10E+11	6.79005	3487.57	1369.68	1
Pfdn5	0.187388	0.0446911	5.35E+11	2.0364	3689.92	4831.96	1
Zswim8	0.208781	0.0453327	5.07E+11	1.89573	3447.95	4850.12	1
Sae1	0.132751	0.0254076	4.86E+11	2.50783	3395.59	3610.66	1
Ctsl	0.189241	0.0395901	3.87E+11	2.02345	3356.31	4423.21	1
Hmgb3	0.135674	0.0270593	3.47E+11	2.47695	3369.43	3627.5	1
Ppil6	0.108038	0.0198249	2.49E+11	2.80757	3224.92	3063.06	1

Glyr1	0.21894	0.0496159	2.04E+11	1.83492	3274.1	4758.2	1
Kank1	0.0815071	0.0144861	2.00E+11	3.24151	3191.71	2625.69	1
Isl1	0.179747	0.040225	1.69E+11	2.09145	3284.7	4188.11	1
Cir1	0.143581	0.0325482	1.08E+11	2.39736	3262.61	3629.12	1
Wnt5a	0.177979	0.036237	1.08E+11	2.10459	3022.44	3829.64	1
Pkn3	0.169731	0.0341921	7.82E+10	2.16808	2947.84	3625.74	1
Acap3	0.113725	0.0221038	6.44E+10	2.73159	2965.46	2894.98	1
Atp1b2	0.195818	0.0438519	3.39E+10	1.97875	2861.31	3856.06	1
Nup133	0.221633	0.0477143	3.12E+10	1.81938	2735.3	4009.14	1
Ddb1	0.029974	0.00624913	1.15E+10	5.04032	2812.09	1487.78	1
Mvd	0.0165292	0.00287121	8.71E+09	6.33761	2605.21	1096.19	1
Gnl2	0.157153	0.0421868	7.66E+09	2.27253	2835.98	3327.84	1
Smim11	0.15409	0.0323448	5.57E+09	2.29952	2433.78	2822.37	1
Tnni1	0.16854	0.0346216	4.23E+09	2.17756	2326.68	2849.28	1
Fam207a	0.0929511	0.0307355	2.35E+09	3.03563	2896.16	2544.15	1
Nek9	0.158045	0.0323248	1.78E+09	2.2648	2158.49	2541.5	1
1600014C10Rik	0.0625708	0.0133953	1.18E+09	3.67573	2257.16	1637.52	1
Nle1	0.00417059	0.000633499	9.77E+08	10.1807	2088.97	547.171	1
Radil	0.0922508	0.016475	9.45E+08	3.04731	2000.55	1750.66	1
Mdh1	0.168427	0.0418039	9.34E+08	2.17847	2228.29	2727.66	1
Flnc	0.00974143	0.00150012	8.01E+08	7.66069	2029.69	706.529	1
Asb1	0.0594788	0.00996323	4.94E+08	3.76213	1878.49	1331.5	1
Ndufa9	0.12126	0.0237477	3.73E+08	2.63782	1861.62	1881.97	1
Iscu	0.161983	0.0365463	3.66E+08	2.23125	1939.61	2318.11	1
Vamp2	0.0983454	0.0178434	3.34E+08	2.94915	1807.92	1634.75	1
Smarcc2	0.186264	0.038332	3.21E+08	2.04432	1796.67	2343.63	1
Vgll4	0.198567	0.0415105	2.43E+08	1.9606	1746.28	2375.16	1
Cwc27	0.113443	0.0221633	2.04E+08	2.73525	1757.39	1713.33	1
Midn	0.125761	0.0239122	1.99E+08	2.58517	1715.74	1769.83	1
Kif23	0.10439	0.0210272	1.04E+08	2.85895	1668.53	1556.31	1
Iqgap3	0.0877337	0.0171708	4.85E+07	3.12537	1531.92	1307.08	1
Idua	0.0872613	0.0154371	4.10E+07	3.13382	1438.88	1224.39	1
Prob1	0.180777	0.0369911	3.53E+07	2.08386	1415.86	1811.84	1
Dynlt1a	0.149952	0.0325499	2.67E+07	2.33704	1440.72	1643.93	1
Cdc123	0.0850278	0.0150029	2.04E+07	3.17455	1327.32	1114.97	1
Tbx5	0.228157	0.0494964	1.89E+07	1.78264	1314.92	1967	1
Tmem42	0.181721	0.0371635	1.33E+07	2.07695	1260.41	1618.28	1
Vipas39	0.104922	0.0193438	8.95E+06	2.85132	1205.25	1127.19	1
Gphn	0.189299	0.0391006	8.47E+06	2.02306	1191.68	1570.8	1
Gnl3l	0.123188	0.0235091	6.10E+06	2.61499	1151.07	1173.82	1
Gtpbp2	0.147176	0.0287211	4.44E+06	2.36293	1097.74	1238.85	1
Arhgap31	0.149694	0.0292935	3.98E+06	2.33943	1081.56	1232.85	1
Parn	0.142227	0.0280432	2.28E+06	2.41061	1012.56	1120.12	1
Prpf38a	0.138838	0.0270817	2.00E+06	2.44443	991.702	1081.86	1
Klhl21	0.142181	0.0353185	1.76E+06	2.41105	1095.29	1211.41	1

Plin3	0.0177217	0.00260928	1.71E+06	6.17581	965.441	416.87	1
Bace1	0.0990455	0.0178996	1.56E+06	2.93836	952.749	864.654	1
Ccs	0.123959	0.0244957	996768	2.60597	913.802	935.086	1
Pisd-ps3	0.133962	0.0277814	963179	2.49494	924.124	987.731	1
Aplf	0.129206	0.0246274	593811	2.54641	829.286	868.449	1
Zfp646	0.131072	0.0398947	496477	2.52594	1016.39	1073.02	1
Rpia	0.0480525	0.00784923	494416	4.13757	806.612	519.862	1
Il6st	0.104779	0.019127	382233	2.85336	773.831	723.2	1
Phka2	0.0773992	0.0136492	208317	3.32431	707.067	567.189	1
Nup205	0.123799	0.026447	193574	2.60783	736.502	753.119	1
Pisd-ps1	0.133958	0.0277784	164539	2.49497	702.252	750.578	1
Sema6c	0.18624	0.0383817	112980	2.04449	634.531	827.632	1
Tpx2	0.154956	0.0321997	103761	2.29182	641.162	746.029	1
Gm6307	0.181524	0.0371132	85052.5	2.07839	603.395	774.183	1
Bpnt1	0.163886	0.0394584	64980	2.2154	632.792	761.689	1
Ttc3	0.0164571	0.00324165	58666.4	6.34786	626.475	263.175	1
Smtn	0.00128999	0.000164354	51680.6	14.6697	552.417	100.419	1
Cep89	0.196905	0.0470675	43549	1.97153	574.845	777.527	1
Magt1	0.190334	0.046063	41411.9	2.0159	575.452	761.219	1
Akap13	0.191698	0.0397205	24360	2.00654	477.791	634.979	1
Npr2	0.0603668	0.0102337	23847.1	3.73676	478.156	341.227	1
Rpp21	0.11689	0.0230551	20118.6	2.69131	472.231	467.907	1
Epb4.1l5	0.0838539	0.0147642	18747.4	3.19649	453.656	378.462	1
Alg9	0.193937	0.0407107	18705.6	1.99134	455.543	610.03	1
Ift81	0.045419	0.00737733	18507	4.24003	453.249	285.061	1
Riok2	0.147811	0.0314795	14611	2.35696	449.48	508.543	1
Adck4	0.132165	0.0271905	8045.08	2.51412	392.64	416.465	1
Zc3h7a	0.10913	0.0208018	7178.03	2.7926	375.274	358.35	1
2700038G22Rik	0.139281	0.0281699	6984.32	2.43996	375.502	410.392	1
Trim24	0.158896	0.0320954	6372.57	2.25746	362.692	428.437	1
Cep192	0.135679	0.0354415	5153.24	2.4769	398.243	428.754	1
Abcb10	0.051643	0.0088381	3419.13	4.00859	315.318	209.762	1
Mios	0.161314	0.0327908	2918.98	2.23688	301.376	359.281	1
Fam76a	0.0589052	0.00996748	2753.99	3.77877	295.454	208.501	1
Nsmaf	0.0210637	0.0046108	2520.31	5.78628	329.585	151.893	1
Cd276	0.0710624	0.012183	2347.96	3.46332	282.147	217.246	1
Piezo1	0.123249	0.02318	2346.58	2.61426	282.056	287.71	1
Pou2f1	0.180843	0.0400232	1871.13	2.08338	277.119	354.705	1
Sdsl	0.0680705	0.0115921	1860.1	3.5344	265.421	200.258	1
Gtf3c6	0.208059	0.0440713	1676.49	1.90019	258.301	362.491	1
Ccnyl1	0.0721355	0.0125163	1590.27	3.43873	255.565	198.186	1
Alms1	0.193848	0.0409558	1261.66	1.99194	240.859	322.444	1
Isg20l2	0.154526	0.0319975	1208.39	2.29563	241.732	280.801	1
Ppfia4	0.180586	0.0368754	1189.93	2.08526	234.856	300.337	1
Kbtbd2	0.18957	0.0438528	991.825	2.02118	236.639	312.213	1

Mysm1	0.0234035	0.00353063	805.445	5.55676	209.911	100.735	1
Pak2	0.193586	0.0471515	792.559	1.99371	227.244	303.948	1
Zfp316	0.0591477	0.00998057	731.464	3.77171	204.552	144.622	1
Slc44a2	0.109864	0.0205081	511.915	2.78265	183.403	175.759	1
Sh3bgr	0.0528113	0.00868646	476.412	3.969	178.065	119.637	1
Trappc2l	0.0686756	0.01194	423.335	3.51971	172.685	130.833	1
Fuz	0.0757458	0.0133037	386.349	3.35919	167.219	132.745	1
Gba	0.118322	0.0223014	382.423	2.67351	166.359	165.933	1
Rab11fip3	0.0651174	0.0111171	274.17	3.60845	148.118	109.46	1
Pank4	0.127064	0.0244962	232.649	2.57035	140.303	145.56	1
Neurl1a	0.0284656	0.0043632	186.244	5.14552	127.952	66.311	1
Zscan26	0.205091	0.043247	158.296	1.91871	120.139	166.972	1
Cdip1	0.0202601	0.00307483	82.1721	5.87255	91.735	41.656	1
Vprbp	0.022719	0.00364239	80.2373	5.62084	92.1975	43.7407	1
Ckap2l	0.0330462	0.00516062	48.7338	4.8451	70.8161	38.9761	1
Rnft2	0.0825375	0.0144717	30.6765	3.22155	54.8872	45.4334	1
Jag1	0.159314	0.048513	26.5568	2.25388	62.9749	74.5085	1
Wbp4	0.102493	0.0415927	25.746	2.88655	72.8266	67.279	1
Phf21a	0.000777518	0.000113314	20.4587	17.0504	44.6159	6.9779	1
Igfbp3	0.164188	0.032791	20.0324	2.2129	42.0735	50.7008	1
Ap5s1	0.189372	0.0391194	12.5858	2.02255	30.0368	39.6025	1
Chmp4b	0.140809	0.0281188	11.7597	2.42464	28.9061	31.7915	1
Vcan	0.0202098	0.00302556	10.3192	5.87809	25.5892	11.6089	1
Tm2d1	0.0939432	0.0171859	9.36638	3.01926	23.657	20.8943	1
Lrch4	0.170565	0.0343643	7.84348	2.16149	19.8673	24.5106	1
Alms1-ps2	0.0613496	0.0103644	7.66522	3.70921	19.4787	14.0038	1
Pdia4	0.0145668	0.00225563	6.26334	6.63835	16.1407	6.48382	1
Cdc27	0.110653	0.0223975	6.13431	2.77203	16.0813	15.47	1
Zcchc7	0.0748554	0.0129366	4.1151	3.37836	9.37476	7.39984	1
Irs1	0.0652105	0.0117281	3.67859	3.60605	8.14529	6.02342	1
Hmgcll1	0.161906	0.0359967	3.37037	2.2319	7.30894	8.73269	1
Phospho2	0.188167	0.0404902	3.07888	2.03094	6.0551	7.95049	1
Dact3	0.10015	0.0286682	3.03179	2.92152	7.11994	6.49885	1
Slc25a17	0.0984863	0.0324835	2.84644	2.94697	6.79246	6.14639	1
Lsm14b	0.167513	0.0434859	2.81718	2.1858	5.74109	7.00411	1
Tor1b	0.20102	0.0479822	2.64295	1.94465	4.74383	6.50514	1
1500012F01Rik	0.0834808	0.0158328	2.60458	3.20354	4.43501	3.69176	1
Fam20b	0.119308	0.0224472	2.5627	2.66141	4.15993	4.16815	1
Emd	0.200861	0.0462544	2.51504	1.94568	4.1894	5.74181	1
Gm561	0.147663	0.0289457	2.3458	2.35835	3.41224	3.85833	1
Paqr4	0.128128	0.0247022	2.23327	2.55841	3.04765	3.17661	1
Rcsd1	0.143608	0.0278585	2.12666	2.3971	2.66653	2.96639	1
Cacnb2	0.0520979	0.00855409	2.09105	3.99304	2.54832	1.70184	1
Gm5039	0.105283	0.0378311	-2.06934	2.84617	3.42529	3.20926	1
Wdhd1	0.0839999	0.0489891	-2.21927	3.19374	5.30184	4.42686	1

Ncapg2	0.00504723	0.0393182	-2.25146	9.5669	14.6289	4.07764	1
Csnk1g1	0.0130286	0.0355353	-3.32123	6.91181	21.9459	8.46703	1
Casd1	0.0179322	0.0432418	-3.38898	6.14865	22.3592	9.69718	1
Gm5176	0.0818021	0.0427118	-4.20104	3.23576	16.1661	13.3229	1
Dcbld2	0.00441472	0.0474812	-4.24738	9.99464	53.6828	14.3231	1
Otud6b	0.0188789	0.029799	-14.7801	6.03126	88.3137	39.0471	1
1700071K01Rik	0.0835977	0.0440647	-24.8238	3.20133	81.4314	67.8314	1
Nop10	0.16074	0.0361954	-39.1759	2.24173	67.0948	79.813	1
Plcxd1	2.63E-05	0.00716452	-59.7216	43.7162	800.497	48.8299	1
Greb1l	0.0473493	0.0437199	-115.605	4.16423	230.723	147.749	1
Gm6607	0.0111353	0.00228684	-233.014	7.30956	157.62	57.5029	1
Rab18	0.0198597	0.0470584	-363.499	5.91714	525.555	236.851	1
Cbx3	0.0159775	0.0144873	-363.883	6.41748	324.54	134.857	1
Braf	0.00965753	0.0305996	-11740.3	7.68381	1379.83	478.869	1
Gtpbp6	0.000111698	0.032777	-20842.8	29.5448	6182.27	558.002	1
Tfip11	4.02E-05	0.00349735	-57942.4	39.003	3944.69	269.702	1
Bzw1	0.0222664	0.04721	-786049	5.66455	2671.89	1257.83	1
Gm15645	0.00645119	0.0117548	-5.80E+08	8.81885	4785.02	1446.91	1
Sqle	0.000624741	0.0452539	-8.41E+08	18.1758	19201.1	2817.09	1
Wdr90	0.0226236	0.0483423	-6.68E+09	5.62996	7471.86	3539.09	1
Tdp1	0.00187884	0.0146893	-2.30E+11	13.0861	13101.7	2669.83	1
Slc30a6	0.0244516	0.0317169	-1.54E+14	5.46303	12137	5924.43	1
Supt5	0.0234613	0.0119733	-1.40E+18	5.55145	13013.2	6250.93	1
Lmnb2	0.0109929	0.0469889	-4.80E+19	7.34285	38502.1	13982.6	1
Fam103a1	0.0114063	0.037016	-4.15E+20	7.24766	36758.1	13524.6	1
Cnih1	6.21E-05	0.035686	-9.70E+20	34.6865	178882	13752.3	1
Hmgn2	0.0301027	0.0184188	-1.62E+25	5.03165	27364.2	14502.4	1
Ube2o	0.101122	0.0383925	-7.41E+27	2.9069	27449.4	25180.9	1
Lars	0.166474	0.0457395	-1.82E+35	2.1942	36388	44223.2	1
Sdhb	0.00208721	0.0080371	-5.88E+39	12.6691	121592	25593.3	1
Rrp1	0.177361	0.0365609	-4.78E+43	2.10922	47705.9	60314.1	1
Tcf3	0.0631954	0.046233	-4.46E+54	3.65892	146619	106858	1
Tcfl5	4.97E-05	0.0136362	-1.14E+57	36.8302	901195	65250.5	1
Cenpa	0.000349386	0.0106976	-7.33E+63	21.4819	595682	73945.3	1
Lsm8	0.00701224	0.0209701	-1.35E+65	8.57486	329940	102607	1
Polr2e	0.0147948	0.0333108	-1.34E+67	6.60089	336355	135883	1
Tars	0.0470861	0.0169246	-3.48E+74	4.17434	191244	122171	1
Marcksl1	0.0361474	0.0372194	-2.57E+87	4.66968	426860	243763	1
Nhp2l1	0.0577452	0.0133379	-6.60E+93	3.81305	249916	174779	1
Ctdsp2	0.0540119	0.0408036	-1.49E+109	3.92943	587769	398883	1
Odc1	0.00537638	0.0127354	-1.70E+156	9.36997	1.67E+06	475382	1
Lyar	0.100077	0.0312445	-1.01E+163	2.92263	850654	776154	1
Hand1	0.00431322	0.0412837	-2.11E+180	10.0704	4.14E+06	1.09E+06	1
Eef1b2	0.025648	0.0207405	-8.99E+307	5.36197	5.44E+06	2.71E+06	1
Tpt1	0.0366609	0.0128125	-8.99E+307	4.64244	6.02E+06	3.46E+06	1
