# Supplementary material for: Single-Cell Expression Profiling Reveals a Dynamic State of Cardiac Precursor Cells in the Early Mouse Embryo
Source: PLoS One. 2015 Oct 15;10(10):e0140831. doi: 10.1371/journal.pone.0140831 (PMC4607431; doi:10.1371/journal.pone.0140831)
Supplement: S9 Table — (PDF) [file pone.0140831.s019.pdf]

Table S9. Gene Ontology enrichment analysis on *Nkx2-5*$^{+}$ EB CPs (*P*<0.05)

GO biological process complete	Background frequency	Sample frequency	expected	Fold Enrichment	+/-	P value	
mesenchyme morphogenesis	31	6	0.4	15	+	2.90E-02	Smad4, Lef1, Snai1, Notch1, Ctnnb1, Rbpj
mRNA processing	338	20	4.42	4.53	+	2.31E-04	C1qbp, Zrsr2, Lsm6, Clns1a, Srrm1, Pabpc2, Prpf4, Kin, Celf6, Cpsf2, Eftud2, Zpr1, Tsen15, Pde12, Ppil3, Pabpc1, Strap, Prpf39, Fam103a1, Scaf4
mRNA metabolic process	395	22	5.16	4.26	+	1.35E-04	C1qbp, Zrsr2, Lsm6, Clns1a, Srrm1, , Auh, Pabpc2, Prpf4, Kin, Celf6, Cpsf2, Eftud2, Mex3d, Zpr1, Tsen15, Pde12, Ppil3, Pabpc1, Strap, Prpf39, Fam103a1, Scaf4
RNA processing	590	23	7.71	2.98	+	2.97E-02	C1qbp, Zrsr2, Lsm6, Wdhd1, Clns1a, Srrm1, Pabpc2, Prpf4, Noc4l, Kin, Celf6, Cpsf2, Eftud2, Zpr1, Tsen15, Pde12, Ppil3, Pabpc1, Strap, Prpf39, Fam103a1, Scaf4, Cirh1a
regulation of transcription from RNA polymerase II promoter	1560	46	20.38	2.26	+	1.52E-03	Klf13, Cbx2,Hmg20a, Insig2, Smad4, C1qbp, E2f8, Ctbp2, Evx1, Lhx2, Rfx1, Taf4a, Scap, Lef1, Lef1, Mtf1, Rnf2, Cbfa2t2, Zpr1, Etv2, Sall4, Per1, Itgb1bp1, Snai1, Wdr61, Skil, Rbck1, Triap1, Foxk2, Setdb1, Strap, Ecm1, Gal, Notch1, Irf1, Ctnnb1, Rbpj, Med14, Hexim1, Strn3, Tcf3, Wwc2, Sap130, Dab2ip, Cry1, Crtc3
RNA metabolic process	2532	74	33.08	2.24	+	1.50E-07	Hmg20a,Zrsr2,Smad4,C1qbp,Lsm6,Klf13,Cbx2,Chaf1b,Wdhd1,E2f8,Clns1a,Ctbp2,Prpf4,Setd1b,Kdm4b,Rfx1,Auh,Taf4a,Lhx2,Srrm1,Pabpc2,Med23,Pcgf3,Kin,Celf6,Lef1,Atoh8,Noc4l,Elp4,Mtf1,Rnf2,Cpsf2,Dna2,Sall4,Eftud2,Zpr1,Mex3d,Atg5,Etv2,Tsen15,Per1,Cdca7,Vps36,Wdr61,Setdb1,Itgb1bp1,Snai1,Cdca7l,Skil,Pde12,Adnp2,Foxk2,Ppil3,Strap,Notch1,Irf1,Zkscan1,Chaf1a,Thap1,Pabpc1,Hexim1,Tcf3,Ctnnb1,Rbpj,Med14,Atmin,Prpf39,Scaf4,Cirh1a,Cry1

							,Crtc3,Fam103a1,Sap130,Mier3
positive regulation of nucleobase-containing compound metabolic process	1408	40	18.39	2.17	+	2.56E-02	Klf13,Insig2,Smad4,E2f8,Ctbp2,Evx1,Nif3l1,Tinf2,Lhx2,Taf4a,Scap,Lef1,Mtf1,Cbfa2t2,Ube2v2,Zpr1,Dna2,Etv2,Sall4,Per1,Itgb1bp1,Snai1,Wdr61,Rbck1,Triap1,Foxk2,Gal,Notch1,Irf1,Pabpc1,Ctnnb1,Rbpj,Rnf219,Med14,Strn3,Cdc42,Tcf3,Atmin,Dab2ip,Crtc3
nucleic acid metabolic process	3007	85	39.28	2.16	+	1.57E-08	Hmg20a,Zrsr2,Smad4,C1qbp,Lsm6,Klf13,Cbx2,Chaf1b,Wdhd1,E2f8,Clns1a,Ctbp2,Prpf4,Setd1b,Kdm4b,Rfx1,Auh,Taf4a,Tinf2,Lhx2,Srrm1,Pabpc2,Med23,Pcgf3,Kin,Ung,Celf6,Lef1,Atoh8,Noc4l,Msh6,Ncapg2,Rfc5,Elp4,Mtf1,Rnf2,Ncapd2,Cpsf2,Dna2,Bahcc1,Sall4,Ube2v2,Eftud2,Zpr1,Mex3d,Atg5,Etv2,Tsen15,Per1,Cdca7,Vps36,Wdr61,Setdb1,Itgb1bp1,Snai1,Cdca7l,Skil,Pde12,Adnp2,Foxk2,Exo1,Ppil3,Strap,Notch1,Irf1,Zkscan1,Chaf1a,Thap1,Pabpc1,Hexim1,Tcf3,Ctnnb1,Rbpj,Med14,Atmin,Prpf39,Scaf4,Cirh1a,Cry1,Crtc3,Fam103a1,Smarcad1,Sap130,Mier3,Dtl
gene expression	2679	74	35	2.11	+	2.01E-06	Hmg20a,Zrsr2,Smad4,C1qbp,Lsm6,Mrpl15,Klf13,Cbx2,Chaf1b,Wdhd1,E2f8,Clns1a,Ctbp2,Prpf4,Setd1b,Kdm4b,Rfx1,Taf4a,Lhx2,Srrm1,Pabpc2,Med23,Pcgf3,Kin,Celf6,Lef1,Atoh8,Noc4l,Elp4,Mtf1,Rnf2,Eif3m,Cpsf2,Sall4,Eftud2,Zpr1,Eif1a,Etv2,Tsen15,Per1,Cdca7,Vps36,Wdr61,Setdb1,Eif2d,Itgb1bp1,Snai1,Cdca7l,Skil,Pde12,Adnp2,Foxk2,Ppil3,Strap,Notch1,Irf1,Zkscan1,Chaf1a,Thap1,Eif1a,Pabpc1,Hexim1,Tcf3,Ctnnb1,Rbpj,Med14,Atmin,Prpf39,Scaf4,Cirh1a,Cry1,Crtc3,Fam103a1,Sap130,Mier3,Eif1a
nucleobase-containing compound metabolic process	3714	99	48.52	2.04	+	3.53E-09	Psmc2,C1qbp,Lsm6,Klf13,Cbx2,Chaf1b,Wdhd1,Clns1a,Hmg20a,Zrsr2,Smad4,Dpysl5,Rab34,E2f8,Ctbp2,Atp6v1g1,Prpf4,Setd1b,Kdm4b,Tinf2,Lhx2,Pabpc2,Rfx1,Auh,T

							af4a,Srrm1,Med23,Pcgf3,Celf6,Noc4l,Rab18,Rfc5,Mtf1,Kin,Ung,Lef1,Atoh8,Msh6,Ncapg2,Elp4,Cpsf2,Dna2,Sall4,Ube2v2,Eftud2,Etv2,Rnf2,Ncapd2,Bahcc1,Zpr1,Mex3d,Atg5,Tsen15,Per1,Wdr61,Papss1,Itgb1bp1,Snai1,Adnp2,Foxk2,Cdca7,Vps36,Setdb1,Cdca7l,Skil,Pde12,Exo1,Notch1,Irf1,Nt5c2,Chaf1a,Thap1,Pabpc1,Ppil3,Strap,Zkscan1,Dlg3,Fut8,Hexim1,Cdc42,Ctnnb1,Adal,Tcf3,Rbpj,Med14,Atmin,Prpf39,Cirh1a,Cry1,Fam103a1,Smarcad1,Sap130,Mier3,Dtl,Cenpe,Scaf4,Crtc3,Spast,Rragc
negative regulation of macromolecule metabolic process	1783	47	23.29	2.02	+	2.46E-02	
RNA biosynthetic process	1901	50	24.83	2.01	+	1.21E-02	
cellular nitrogen compound metabolic process	3977	103	51.96	1.98	+	5.60E-09	
transcription, DNA-templated	1894	49	24.74	1.98	+	2.47E-02	
nucleic acid-templated transcription	1894	49	24.74	1.98	+	2.47E-02	
cellular aromatic compound metabolic process	3880	100	50.69	1.97	+	1.98E-08	
heterocycle metabolic process	3848	99	50.27	1.97	+	3.01E-08	
organic cyclic compound metabolic process	4065	103	53.11	1.94	+	2.19E-08	
nitrogen compound metabolic process	4295	108	56.11	1.92	+	7.50E-09	
regulation of transcription, DNA-templated	2798	70	36.55	1.92	+	4.06E-04	
nucleobase-containing compound biosynthetic process	2121	53	27.71	1.91	+	2.52E-02	
regulation of nucleic acid-templated transcription	2811	70	36.72	1.91	+	4.87E-04	
regulation of RNA biosynthetic process	2817	70	36.8	1.9	+	5.29E-04	
regulation of RNA metabolic process	2899	72	37.87	1.9	+	3.29E-04	
regulation of nucleobase-containing compound metabolic process	3156	78	41.23	1.89	+	8.97E-05	
cellular nitrogen compound biosynthetic process	2229	55	29.12	1.89	+	2.31E-02	
organelle organization	2322	57	30.33	1.88	+	1.72E-02	
cellular macromolecule biosynthetic process	2589	63	33.82	1.86	+	5.92E-03	
regulation of cellular macromolecule biosynthetic process	3061	74	39.99	1.85	+	6.02E-04	
macromolecule	2613	63	34.14	1.85	+	8.09E-	

biosynthetic process						03	
cellular macromolecule metabolic process	5071	122	66.25	1.84	+	2.06E-09	
regulation of gene expression	3350	80	43.76	1.83	+	2.47E-04	
regulation of nitrogen compound metabolic process	3377	80	44.12	1.81	+	3.50E-04	
regulation of macromolecule biosynthetic process	3173	74	41.45	1.79	+	2.50E-03	
cellular component organization	3866	90	50.51	1.78	+	7.81E-05	
cellular component organization or biogenesis	3998	93	52.23	1.78	+	4.04E-05	
regulation of cellular biosynthetic process	3287	74	42.94	1.72	+	9.66E-03	
macromolecule metabolic process	5678	126	74.18	1.7	+	2.24E-07	
cellular biosynthetic process	3254	72	42.51	1.69	+	2.64E-02	
regulation of biosynthetic process	3358	74	43.87	1.69	+	2.14E-02	
regulation of macromolecule metabolic process	4530	99	59.18	1.67	+	2.61E-04	
organic substance biosynthetic process	3349	73	43.75	1.67	+	3.78E-02	
biosynthetic process	3415	74	44.61	1.66	+	3.93E-02	
cellular metabolic process	6983	149	91.23	1.63	+	1.15E-08	
primary metabolic process	7178	151	93.77	1.61	+	2.28E-08	
regulation of primary metabolic process	4476	94	58.47	1.61	+	4.59E-03	
regulation of cellular metabolic process	4677	98	61.1	1.6	+	2.54E-03	
regulation of metabolic process	5339	111	69.75	1.59	+	3.63E-04	
organic substance metabolic process	7455	153	97.39	1.57	+	1.12E-07	
metabolic process	8031	160	104.92	1.53	+	2.66E-07	
cellular process	12681	209	165.66	1.26	+	7.68E-04	
biological_process	20617	287	269.34	1.07	+	1.72E-02	
Unclassified	1658	4	21.66	< 0.2	-	0.00E+00	
neurological system process	1822	4	23.8	< 0.2	-	2.49E-03	
sensory perception	1525	2	19.92	< 0.2	-	1.91E-03	
sensory perception of smell	1099	1	14.36	< 0.2	-	4.54E-02	
detection of stimulus involved in sensory perception	1173	1	15.32	< 0.2	-	1.75E-02	
detection of stimulus	1266	1	16.54	< 0.2	-	5.22E-03	
G-protein coupled receptor signaling pathway	1840	1	24.04	< 0.2	-	2.42E-06	
