# Supplementary material for: Single-Cell Expression Profiling Reveals a Dynamic State of Cardiac Precursor Cells in the Early Mouse Embryo
Source: PLoS One. 2015 Oct 15;10(10):e0140831. doi: 10.1371/journal.pone.0140831 (PMC4607431; doi:10.1371/journal.pone.0140831)
Supplement: S10 Table — (PDF) [file pone.0140831.s020.pdf]

Table S10. Gene Ontology enrichment analysis on *Tbx5*$^{+}$ EB CPs (P<0.05)

GO biological process complete	Background frequency	Sample frequency	expected	Fold Enrichment	+/-	P value	
posttranscriptional regulation of gene expression	414	17	4.55	3.73	+	3.15E-02	Ythdf2,Nckap1,Msx1,Ireb2,Zfp36l1,Pcbp1,Stxbp1,Mapkapk5,Ncbp1,Chp1,Eif4ebp2,Hnrnpa0,Ppp1ca,Cnot11,Magoh,Eif4g2,Syncrip
nucleoside metabolic process	613	22	6.74	3.26	+	1.10E-02	Nudt1,Gnai3,Top2a,Cmpk1,Uqcr10,Rap2c,Gtpbp1,Abcb7,Ascc3,Dhx15,Ide,Atp6v0c,Upp1,Abcb6,Rab2b,Hsp90aa1,Ak4,Nras,Aldoa,Dera,Pycrl,Nt5c3
glycosyl compound metabolic process	626	22	6.89	3.2	+	1.53E-02	Nudt1,Gnai3,Top2a,Cmpk1,Uqcr10,Rap2c,Gtpbp1,Abcb7,Ascc3,Dhx15,Ide,Atp6v0c,Upp1,Abcb6,Rab2b,Hsp90aa1,Ak4,Nras,Aldoa,Dera,Pycrl,Nt5c3
ribonucleoside metabolic process	602	21	6.62	3.17	+	2.95E-02	Nudt1,Gnai3,Top2a,Cmpk1,Uqcr10,Rap2c,Gtpbp1,Abcb7,Ascc3,Dhx15,Ide,Atp6v0c,Upp1,Abcb6,Rab2b,Hsp90aa1,Ak4,Nras,Aldoa,Pycrl,Nt5c3
nucleobase-containing compound catabolic process	667	23	7.34	3.14	+	1.22E-02	Nudt1,Gnai3,Top2a,Rap2c,Zfp36l1,Xrn2,Gtpbp1,Itpa,Abcb7,Ascc3,Dhx15,Ide,Ncbp1,Atp6v0c,Upp1,Abcb6,Rab2b,Hsp90aa1,Magoh,Eif3e,Nras,Dera,Pycrl
nucleotide metabolic process	760	26	8.36	3.11	+	2.89E-03	Nudt1,Gnai3,Top2a,Cmpk1,Uqcr10,Rap2c,Itpa,Gtpbp1,Abcb7,Ascc3,Ide,Dhx15,Ugp2,Abcb6,Gnpnat1,Atp6v0c,Upp1,Rab2b,Hsp90aa1,Paics,Aldoa,Mthfd1,Ak4,Nras,Dera,Nt5c3
nucleoside phosphate metabolic process	769	26	8.46	3.07	+	3.59E-03	Nudt1,Gnai3,Top2a,Cmpk1,Uqcr10,Rap2c,Itpa,Gtpbp1,Abcb7,Ascc3,Ide,Dhx15,Ugp2,Abcb6,Gnpnat1,Atp6v0c,Upp1,Rab2b,Hsp90aa1,Paics,Aldoa,Mthfd1,Ak4,Nras,Dera,Nt5c3
nucleobase-containing small molecule metabolic process	817	27	8.99	3	+	3.31E-03	Nudt1,Gnai3,Top2a,Cmpk1,Uqcr10,Rap2c,Itpa,Gtpbp1,Abcb7,Ascc3,Ide,Dhx15,Ugp2,Abcb6,Gnpnat1,Atp6v0c,Upp1,Rab2b,Hsp90aa1,Paics,Aldoa,Mthfd1,Ak4,Nras,Dera,Pycrl,Nt5c3
cellular nitrogen compound catabolic process	699	23	7.69	2.99	+	2.64E-02	Nudt1,Gnai3,Top2a,Rap2c,Zfp36l1,Xrn2,Gtpbp1,Itpa,Abcb7,Ascc3,Dhx15,Ide,Ncbp

							1,Atp6v0c,Upp1,Abcb6,Rab2b,Hsp90aa1,Magoh,Eif3e,Nras,Dera,Pycrl
purine-containing compound metabolic process	674	22	7.41	2.97	+	4.89E-02	Nudt1,Gnai3,Top2a,Uqcr10,Rap2c,Gtpbp1,Itpa,Abcb7,Ascc3,Dhx15,Ide,Lrrc16a,Atp6v0c,Abcb6,Rab2b,Hsp90aa1,Paics,Ak4,Nras,Mthfd1,Aldoa,Nt5c3
heterocycle catabolic process	705	23	7.75	2.97	+	3.03E-02	
aromatic compound catabolic process	709	23	7.8	2.95	+	3.32E-02	
organophosphate metabolic process	1039	29	11.43	2.54	+	3.41E-02	
organic substance catabolic process	1511	39	16.62	2.35	+	4.64E-03	
organonitrogen compound metabolic process	1380	35	15.18	2.31	+	2.75E-02	
cellular catabolic process	1494	37	16.43	2.25	+	2.42E-02	
catabolic process	1711	40	18.82	2.13	+	3.68E-02	
nucleobase-containing compound metabolic process	3714	84	40.85	2.06	+	1.21E-07	
heterocycle metabolic process	3848	87	42.32	2.06	+	4.30E-08	
cellular nitrogen compound metabolic process	3977	89	43.74	2.03	+	3.75E-08	
cellular aromatic compound metabolic process	3880	86	42.68	2.02	+	1.77E-07	
organic cyclic compound metabolic process	4065	90	44.71	2.01	+	4.82E-08	
cellular protein metabolic process	2440	53	26.84	1.97	+	7.39E-03	
nitrogen compound metabolic process	4295	92	47.24	1.95	+	1.58E-07	
nucleic acid metabolic process	3007	60	33.07	1.81	+	1.93E-02	
cellular macromolecule metabolic process	5071	97	55.78	1.74	+	1.97E-05	
cellular metabolic process	6983	133	76.81	1.73	+	6.65E-10	
single-organism metabolic process	3648	68	40.12	1.69	+	3.62E-02	
primary metabolic process	7178	129	78.95	1.63	+	2.25E-07	
organic substance metabolic process	7455	132	82	1.61	+	3.00E-07	
macromolecule metabolic process	5678	99	62.45	1.59	+	1.62E-03	
metabolic process	8031	138	88.33	1.56	+	6.00E-07	
regulation of metabolic process	5339	90	58.72	1.53	+	3.78E-02	
cellular process	12681	176	139.48	1.26	+	7.17E-03	
Unclassified	1658	8	18.24	0.44	-	0.00E+00	
G-protein coupled receptor signaling pathway	1840	3	20.24	< 0.2	-	9.54E-03	
