# Supplementary material for: Single-Cell Expression Profiling Reveals a Dynamic State of Cardiac Precursor Cells in the Early Mouse Embryo
Source: PLoS One. 2015 Oct 15;10(10):e0140831. doi: 10.1371/journal.pone.0140831 (PMC4607431; doi:10.1371/journal.pone.0140831)
Supplement: S11 Table — (PDF) [file pone.0140831.s021.pdf]

**Table S11. Gene Ontology enrichment analysis on *Nkx2-5*$^+$ FHF CPs ($P$<0.05)**

| GO biological process complete | Background frequency | Sample frequency | expected | Fold Enrichment | +/- | P value | |
|---|---|---|---|---|---|---|---|
| growth | 390 | 13 | 2.75 | 4.73 | + | 3.31E-02 | Acvr2b,Itgav,Fgf10,Gins4,Igsf10,Tenc1,Vps54,Foxp2,Mapt,Spry2,Gnas,Plau,Prlr |
| nucleobase-containing compound metabolic process | 3714 | 50 | 26.18 | 1.91 | + | 1.58E-02 | Ccar1,Celf2,Foxn3,Jmjd1c,Rad51d,Mbnl1,Prdx5,Gins4,Gtf2a1,Rps21,Zscan21,Tceb3,Dimt1,Zgpat,Atxn1,Smarcc1,Rbfa,Kif3a,Piwil1,Sp8,Zhx2,Mc1r,Aprt,Polr2i,Foxp2,Hnrnpl,Zscan12,Nfia,Tubb5,Klhl31,Tars,Dpysl2,Npas3,Nacc2,Trmt1,Rbm8a,Tef,Hoxc8,Runx1t1,Atf7ip,Gnas,Nhp2,Gins2,Shfm1,Ndufc2,Galt,Mfn2,Polr2e,Cstf2t,Hmgn5 |
| cellular nitrogen compound metabolic process | 3977 | 53 | 28.03 | 1.89 | + | 9.10E-03 | Ccar1,Celf2,Foxn3,Jmjd1c,Rad51d,Smpd1,Mbnl1,Prdx5,Gins4,Gtf2a1,Rps21,Zscan21,Tceb3,Sod1,Dimt1,Zgpat,Atxn1,Smarcc1,Rbfa,Kif3a,Piwil1,Sp8,Zhx2,Mc1r,Aprt,Polr2i,Foxp2,Hnrnpl,Zscan12,Nfia,Tubb5,Klhl31,Tars,Dpysl2,Npas3,Ggt5,Nacc2,Trmt1,Rbm8a,Tef,Hoxc8,Runx1t1,Atf7ip,Gnas,Nhp2,Gins2,Shfm1,Ndufc2,Galt,Mfn2,Polr2e,Cstf2t,Hmgn5 |
| heterocycle metabolic process | 3848 | 50 | 27.12 | 1.84 | + | 4.40E-02 | Ccar1,Celf2,Foxn3,Jmjd1c,Rad51d,Mbnl1,Prdx5,Gins4,Gtf2a1,Rps21,Zscan21,Tceb3,Dimt1,Zgpat,Atxn1,Smarcc1,Rbfa,Kif3a,Piwil1,Sp8,Zhx2,Mc1r,Aprt,Polr2i,Foxp2,Hnrnpl,Zscan12,Nfia,Tubb5,Klhl31,Tars,Dpysl2,Npas3,Nacc2,Trmt1,Rbm8a,Tef,Hoxc8,Runx1t1,Atf7ip,Gnas,Nhp2,Gins2,Shfm1,Ndufc2,Galt,Mfn2,Polr2e,Cstf2t,Hmgn5 |
| anatomical structure development | 3943 | 51 | 27.79 | 1.84 | + | 3.87E-02 | Acvr2b,Atat1,Fgf10,Itgav,Matk,Rapgef4,Igsf10,Tenc1,Jagn1,Caprin2,Chsy1,Fgf8,Fgf9,Mbnl1,Slitrk2,Gins4,Ecsit,Rbm45,Tpt1,Prex2,Zscan21,Sod1,Gas7,Atxn1,Smarcc1,Kif3a,Piwil1,Apoe,Sp8,Zhx2,Aprt,Foxp2,Nfia,Dpysl2,Nrgn,Fmr1,Mapt,Ncan,Hoxc8,Cby1,Cd63,Shank1,Spry2,Gnas,Plau,Prlr,Mfn2,Sarm1,Acta1,Krt10,Pafah1b3 |
| nitrogen compound metabolic process | 4295 | 55 | 30.27 | 1.82 | + | 1.83E-02 | Ccar1,Celf2,Foxn3,Jmjd1c,Rad51d,Smpd1,Chsy1,Mbnl1,Prdx5,Gins4,Gtf2a1,Rps21,Zscan21,Tceb3,Sod1,Dimt1,Zgpat,Atxn1,Smarcc1,Rbfa,Kif3a,Piwil1,Sp8,Zhx2,Mc1r,Aprt,Polr2i,Foxp2,Hnrnpl,Zscan12,Nfia,Tubb5,Klhl31,Tars,Dpysl2,Npas3,Ggt5,Nacc2,Trmt1,Rbm8a,Tef,Hoxc8,Runx1t1,Atf7ip,Gnas,Nhp2,Gins2,Ptdss2,Shfm1,Ndufc2,Galt,Mfn2,Polr2e,Cstf2t,Hmgn5 |

| | | | | | | | |
|---|---|---|---|---|---|---|---|
| organic cyclic compound metabolic process | 4065 | 52 | 28.65 | 1.81 | + | 4.13E-02 | Ccar1,Celf2,Foxn3,Jmjd1c,Rad51d,Mbnl1,Prdx5,Gins4,Gtf2a1,Rps21,Zscan21,Tceb3,Dimt1,Zgpat,Atxn1,Smarcc1,Rbfa,Kif3a,Piwil1,Sp8,Apoe,Zhx2,Mc1r,Aprt,Polr2i,Foxp2,Hnrnpl,Zscan12,Nfia,Tubb5,Klhl31,Tars,Dpysl2,Npas3,Nacc2,Trmt1,Rbm8a,Tef,Hoxc8,Runx1t1,Atf7ip,Stard3,Gnas,Nhp2,Gins2,Shfm1,Ndufc2,Galt,Mfn2,Polr2e,Cstf2t,Hmgn5 |
| cellular metabolic process | 6983 | 76 | 49.22 | 1.54 | + | 4.29E-02 | Acvr2b,Ccar1,Atat1,Celf2,Foxn3,Jmjd1c,Rad51d,Fgf10,Itgav,Alg2,Sdhc,Matk,Smpd1,Ggcx,Cdc42ep5,Chsy1,Fgf8,Mbnl1,Prdx5,Rps13,Gins4,Gtf2a1,Rps21,Zscan21,Btg1,Tceb3,Sod1,Dimt1,Zgpat,Atxn1,Smarcc1,Rbfa,Kif3a,Piwil1,Apoe,Sp8,Zhx2,Cul2,Mc1r,Aprt,Polr2i,Foxp2,Hnrnpl,Sdhb,Nfia,Zscan12,Tubb5,Klhl31,Dpysl2,Tars,Npas3,Ggt5,Nacc2,Trmt1,Rbm8a,Tef,Rplp2,Lyplal1,Hoxc8,Runx1t1,Atf7ip,Fundc1,Stard3,Gnas,Nhp2,Gins2,Ptdss2,Shfm1,Ndufc2,Galt,Mfn2,Ptp4a3,Msl1,Polr2e,Cstf2t,Hmgn5 |
| metabolic process | 8031 | 85 | 56.6 | 1.5 | + | 2.02E-02 | Acvr2b,Ccar1,Atat1,Celf2,Foxn3,Jmjd1c,Rad51d,Fgf10,Itgav,Alg2,Sdhc,Matk,Tenc1,Smpd1,Ggcx,Cdc42ep5,Chsy1,Fgf8,Mbnl1,Prdx5,Rps13,Gins4,Ecsit,Gtf2a1,Rps21,Zscan21,Btg1,Tceb3,Sod1,Adam23,Dimt1,Zgpat,Atxn1,Smarcc1,Rbfa,Kif3a,Piwil1,Apoe,Sp8,Zhx2,Cul2,Mc1r,Aprt,Polr2i,Alg11,Foxp2,Hnrnpl,Sdhb,Nfia,Zscan12,Tubb5,Klhl31,Dpysl2,Tars,Npas3,Ggt5,Nacc2,Trmt1,Nxf1,Rbm8a,Tef,Rplp2,Lyplal1,Hoxc8,Runx1t1,Atf7ip,Fundc1,Stard3,Gnas,Nhp2,Gins2,Ptdss2,Shfm1,Mpnd,Plau,Ndufc1,Ndufc2,Galt,Mfn2,Ptp4a3,Msl1,Polr2e,Cstf2t,Hmgn5,Pafah1b3 |
| Unclassified | 1658 | 7 | 11.69 | 0.6 | - | 0.00E+00 | Rpl12,Nudt14,Gm2022,Snrpn,Snurf,Glcci1,Dnajc4 |
