# Supplementary material for: Single-Cell Expression Profiling Reveals a Dynamic State of Cardiac Precursor Cells in the Early Mouse Embryo
Source: PLoS One. 2015 Oct 15;10(10):e0140831. doi: 10.1371/journal.pone.0140831 (PMC4607431; doi:10.1371/journal.pone.0140831)
Supplement: S12 Table — (PDF) [file pone.0140831.s022.pdf]

Table S12. Gene Ontology enrichment analysis on *Nkx2-5*$^{+}$/*Tbx5*$^{+}$ FHF CPs (*P*<0.05)

GO biological process complete	Background frequency	Sample frequency	expected	Fold Enrichment	+/-	P value	
cardiac ventricle morphogenesis	69	8	0.89	8.988764045	+	2.78E-02	Jag1, Tnnc1, Gata4, Med1, Isl1, Tbx5, Smarcd3, Tnni1
cardiac chamber morphogenesis	112	10	1.44	6.944444444	+	1.77E-02	Wnt5a, Jag1, Tnnc1, Gata4, Med1, Isl1, Tbx5, Gata6, Smarcd3, Tnni1
cardiac chamber development	129	11	1.66	6.626506024	+	8.66E-03	Smad4, Wnt5a, Jag1, Tnnc1, Gata4, Med1, Isl1, Tbx5, Gata6, Smarcd3, Tnni1
regulation of cell cycle process	381	21	4.89	4.29	+	2.53E-04	Tpx2, Klhl21, Brd4, Csnk2a2, Wnt5a, Bub1b, Calr, Ddb1, Cep192, Kif23, Ilkap, Men1, Crlf3, Pdxp, Med1, Prpf19, Zfyve19, Gata6, Rps6ka2, Smarcd3, Cdc27
chromatin modification	466	20	5.98	3.34	+	2.48E-02	Actb, Kat7, Brd4, Pbrm1, Smarcc2, Hmgb3, Ddb1, Kdm3b, Men1, Dmap1, Chd6, Kdm1a, Mysm1, Phf21a, L3mbtl2, Chd2, Kansl2, Brms1, Vprbp, Smarcd3
regulation of cell cycle	685	29	8.8	3.3	+	1.96E-04	Brd4, Tpx2, Klhl21, Wnt5a, Bub1b, Calr, Ddb1, Csnk2a2, Taf6, Cep192, Kif23, Ilkap, Men1, Bcr, Crlf3, Pdxp, Med1, Sae1, Cdkn1c, Prpf19, Nle1, Fntb, Zfyve19, Npr2, Gata6, Rps6ka2, Smarcd3, Trim35, Cdc27
macromolecular complex subunit organization	1634	48	20.98	2.29	+	5.17E-04	Smad4,Brd4,Kat7,Actb,Tpx2,Vamp2,Nup98,Chd3,Chmp4b,Pbrm1,Calr,Smarcc2,Hmgb3,Ddb1,Prpf31,Zyx,Cep192,Irs1,Kif23,Rrm1,Kdm3b,Men1,Pdxp,Dmap1,Chd6,Serpinh1,Gphn,Prpf19,Kdm1a,Mysm1,Phf21a,L3mbtl2,Chd2,Tor1b,Patl1,Ube2s,Kansl2,Nup205,Brms1,Nup133,Vprbp,Smarcd3,Dync1h1,Rpsa,Akap2,Tube1,Tuba1a,Cenpt
organelle organization	2322	64	29.81	2.15	+	2.45E-05	Actb,Kat7,lhl21,Brd4,Htra2,Tpx2,Vamp2,Nup98,Cep89,Nek9,Chmp4b,Hmgb3,Ddb1,Zyx,Chd3,Pbrm1,Bub1b,Calr,Smarcc2,Kif23,Cep192,Alms1,Rab31,Kdm3b,Men1,Timm50,Bcr,Pdxp,Dmap1,Ykt6,Mysm1,Phf21

							a,L3mbtl2,Chd2,Tor1b,Patl1,Ube2s,Kansl2,Nup205,Brms1,Nup133,Vprbp,Smarcd3,Dync1h1,Rpsa,Akap2,Tube1,Tuba1a,Cenpt,Chd6,Lman1,Camsap1,Kdm1a,Fuz,Phf21a,Mysm1,Pacs2,L3mbtl2,Elmo1,Tor1b,Timm9,Kansl2,Pdlim7,Brms1,Chd2,Patl1,Ube2s,Nup205,Nup133,Vprbp,Smarcd3,Dync1h1,Rpsa,Fmnl3,Cenpt,Idua,Fam160a2,Akap2,Pip5k1a,Cdc27,Sec61a1,Nisch,Ift81
phosphate-containing compound metabolic process	1969	52	25.28	2.06	+	3.71E-03	Adck4,Brd4,Ror2,Pank4,Phka2,Ptdss1,Nek9,Wnt5a,Iqgap3,Bub1b,Csnk2a2,Kif23,Cdc14b,Ilkap,Pmvk,Rrm1,Gars,Fabp5,Rab31,Phospho2,Men1,Timm50,Bcr,Pdxp,Med1,Chd6,Tollip,Fam20b,Gnl2,Gnl3l,Gphn,Npr2,Trim24,B3galnt2,Mdh1,Bpnt1,Igfbp3,Glyr1,Mvd,Tor1b,Rpia,Pkn3,Rps6ka2,Pak2,Gata6,Vprbp,Tube1,Atp1b2,Tuba1a,Srpk1,Pip5k1a,Abcb10
phosphorus metabolic process	2014	53	25.86	2.05	+	3.14E-03	Adck4,Brd4,Ror2,Pank4,Phka2,Ptdss1,Nek9,Wnt5a,Fasn,Iqgap3,Bub1b,Csnk2a2,Kif23,Cdc14b,Ilkap,Pmvk,Rrm1,Fabp5,Gars,Rab31,Phospho2,Men1,Timm50,Bcr,Pdxp,Med1,Chd6,Tollip,Fam20b,Gnl2,Gnl3l,Gphn,Npr2,Trim24,B3galnt2,Mdh1,Bpnt1,Igfbp3,Glyr1,Mvd,Tor1b,Rpia,Pkn3,Rps6ka2,Pak2,Gata6,Vprbp,Tube1,Atp1b2,Tuba1a,Srpk1,Pip5k1a,Abcb1,
RNA metabolic process	2532	59	32.51	1.81	+	3.17E-02	
cellular component organization or biogenesis	3998	93	51.33	1.81	+	1.53E-05	
cellular component organization	3866	87	49.64	1.75	+	3.14E-04	
nucleobase-containing compound metabolic process	3714	82	47.69	1.72	+	2.08E-03	
cellular nitrogen compound metabolic process	3977	86	51.06	1.68	+	2.31E-03	
heterocycle metabolic process	3848	83	49.41	1.68	+	4.57E-03	
negative regulation of cellular process	3498	75	44.91	1.67	+	2.48E-02	
nitrogen compound metabolic process	4295	92	55.15	1.67	+	1.11E-03	

cellular aromatic compound metabolic process	3880	82	49.82	1.65	+	1.26E-02	
single-organism metabolic process	3648	77	46.84	1.64	+	3.14E-02	
cellular macromolecule metabolic process	5071	107	65.11	1.64	+	1.20E-04	
organic cyclic compound metabolic process	4065	85	52.19	1.63	+	1.16E-02	
cellular metabolic process	6983	145	89.66	1.62	+	5.88E-08	
primary metabolic process	7178	142	92.16	1.54	+	5.31E-06	
metabolic process	8031	158	103.11	1.53	+	2.15E-07	
organic substance metabolic process	7455	145	95.72	1.51	+	1.01E-05	
macromolecule metabolic process	5678	110	72.9	1.51	+	6.75E-03	
cellular process	12681	219	162.82	1.35	+	2.16E-08	
single-organism cellular process	10337	170	132.72	1.28	+	4.59E-02	
single-organism process	11811	193	151.65	1.27	+	3.33E-03	
Unclassified	1658	6	21.29	0.28	-	0.00E+00	
G-protein coupled receptor signaling pathway	1840	5	23.62	0.21	-	1.54E-02	
detection of stimulus	1266	1	16.25	< 0.2	-	6.88E-03	
